# Clinical and Economic Outcomes of Dronedarone Versus Amiodarone Among Patients With Atrial Fibrillation

**DOI:** 10.1161/JAHA.125.042178

**Published:** 2025-12-03

**Authors:** Benjamin A. Steinberg, Firas Dabbous, Ron Preblick, Divya Shridharmurthy, David S. McKindley, Jason Rashkin, Samuel Huse, Chris Colby, Jagmeet P. Singh

**Affiliations:** ^1^ Denver Health Medical Center Denver CO USA; ^2^ University of Colorado Anschutz Medical Campus Aurora CO USA; ^3^ University of Utah Salt Lake City UT USA; ^4^ Evidera Bethesda MD USA; ^5^ Sanofi Morristown NJ USA; ^6^ Cardiology Mass General Hospital Boston MA USA

**Keywords:** antiarrhythmic drug, comparative effectiveness, health care resource use, real‐world evidence, safety, Epidemiology, Cardiovascular Disease

## Abstract

**Background:**

This retrospective observational study compared adverse events (AEs) and health care resource use among patients with atrial fibrillation treated with dronedarone versus amiodarone.

**Methods:**

Adults with atrial fibrillation who initiated dronedarone or amiodarone between January 1, 2010 and September 30, 2021 were propensity score matched in Optum’s deidentified Clinformatics Data Mart Database. Outcomes included AEs (described in the dronedarone/amiodarone Food and Drug Administration labels and reported in the Food and Drug Administration AE Reporting System) and all‐cause and cardiovascular‐related health care resource use. Generalized linear models with Poisson distribution were used to compare the risk of AEs between the 2 cohorts. After matching, 12 210 dronedarone‐treated patients were paired 1:1 with amiodarone‐treated patients. For each AE, patients who experienced the AE during baseline were excluded.

**Results:**

During follow‐up, lower event rates of AEs were observed with dronedarone versus amiodarone; the rate ratio for any cardiac and vascular AE was 0.71 (95% CI, 0.69–0.72), any respiratory AE was 0.65 (95% CI, 0.63–0.66), and any gastrointestinal/hepatobiliary AE was 0.81 (95% CI, 0.79–0.84). Compared with amiodarone‐treated patients, dronedarone‐treated patients experienced lower event rates of all‐cause hospitalization (0.69 [95% CI, 0.67–0.71]) and all‐cause outpatient visits (0.87 [95% CI, 0.87–0.87]). Although the incidence of cardiovascular‐related hospitalization was higher with dronedarone versus amiodarone, event rates were not statistically different. Cardiovascular‐related outpatient visits were significantly reduced with dronedarone versus amiodarone with an event rate of 0.95 (95% CI, 0.94–0.96).

**Conclusions:**

In this study, lower event/incidence rates of AEs and health care resource use were observed in patients with atrial fibrillation treated with dronedarone versus amiodarone.

**Nonstandard Abbreviations and Acronyms**
AAD: antiarrhythmic drug
AE: adverse event
FDA: Food and Drug Administration
HCRU: health care resource use
NCO: negative control outcome

**Clinical Perspective**

**What Is New?**

- In a large real‐world evidence study of >24 000 adult patients with atrial fibrillation, dronedarone was found to be associated with lower incidence of commonly reported adverse events.
- The study included a comprehensive list of adverse events that were frequently listed in the Food and Drug Administration Adverse Event Reporting System and those included in the Food and Drug Administration labels for amiodarone and dronedarone.

**What Are the Clinical Implications?**

- The persistent gap between guideline recommendations and amiodarone’s widespread real‐world use highlights the importance of reassessing safety in everyday practice; real‐world comparative safety data support dronedarone as a viable option to reduce adverse event burden and guide more individualized prescribing.

Atrial fibrillation (AF) is the most common sustained cardiac arrhythmia globally^1^, ^2^, ^3^ and is responsible for significant morbidity, resource use, and loss of quality of life. Multiple cardiovascular and cerebrovascular comorbidities are associated with AF, including hypertension, ischemic heart disease, ischemic stroke, and congestive heart failure (HF). In addition, there is an increased risk of dementia and all‐cause mortality in patients with AF compared with individuals without AF.^4^, ^5^

The chronic nature of AF and associated comorbidities impose high costs and health care resource use (HCRU) burden. Patients diagnosed with AF are twice as likely to experience an all‐cause hospitalization and 8 times more likely to experience a cardiovascular‐related hospitalization compared with patients without an AF diagnosis.^4^ Recent data indicate ≈450 000 hospitalizations annually in the United States are due to AF^6^ and the aging population is the most important nonmodifiable risk factor. The annual costs in 2005 US dollars for AF–related hospitalizations were $2.93 billion.^7^

Antiarrhythmic drugs (AADs) are a centerpiece of rhythm control for AF and include dofetilide, dronedarone, flecainide, propafenone, and sotalol.^8^ Given the potential of cardiovascular and noncardiovascular toxicities associated with AADs, current guidelines recommend tailored AAD therapy for AF based on electrocardiogram findings and presence of other comorbidities such as HF and coronary artery disease.^9^, ^10^ Specifically, guidelines recommend not using dronedarone for treatment of AF in patients with New York Heart Association class III and IV HF or in patients who have had an episode of decompensated HF in the past 4 weeks. In addition, because of the toxicity profile of amiodarone, the guidelines recommend using it after consideration of risks and when other agents have failed or are contraindicated. Despite these recommendations, in the United States, amiodarone is the most widely prescribed AAD for AF at discharge.^11^

Few studies have compared the effectiveness and safety of dronedarone with other AADs. There is specifically a lack of head‐to‐head comparative effectiveness studies examining the safety of dronedarone versus amiodarone. This study aimed to fill this gap through understanding incidence and event rates of adverse events (AEs) and HCRU in patients diagnosed with AF and treated with either dronedarone or amiodarone. The focus on amiodarone and dronedarone was deliberate, as dronedarone, a benzofuran derivative, was developed with a similar pharmacological profile to amiodarone in order to mitigate its known toxicities. Thus, a direct comparison of the safety profiles of these 2 agents addresses a clinically important question. Inclusion of additional AADs would have introduced substantial heterogeneity in both patient characteristics and safety profiles and was beyond the scope of the present study.

## METHODS

This study analyzed deidentified data from Optum’s Clinformatics Data Mart Database (CDM)^12^; institutional review board approval and informed consent were not required. The study objectives, prespecified hypotheses, and statistical analysis plan were defined before study initiation as part of a detailed protocol. Adverse events of interest were selected based on a comprehensive literature review and alignment with Food and Drug Administration (FDA) labeling.

Analytical methods and study materials are available in the data supplement.

### Study Design

**Figure 1:**
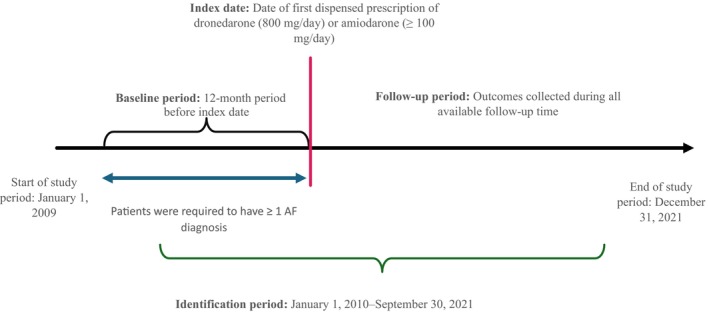
Study design. AF indicates atrial fibrillation.

The study design was an observational study of retrospectively identified, propensity score‐matched cohorts obtained from Optum’s deidentified CDM. CDM is derived from a database of administrative health claims for members of large commercial and Medicare Advantage health plans. CDM uses medical and pharmacy claims to derive patient‐level enrollment information, health care costs, and resource use information. The population is geographically diverse, spanning all 50 states and is statistically deidentified under the Expert Determination method consistent with the Health Insurance Portability and Accountability Act and managed according to Optum customer data use agreements. CDM administrative claims submitted for payment by providers and pharmacies are verified, adjudicated, and deidentified before inclusion. Figure 1 outlines the study design. The design incorporated key principles of the Target trial emulation (TTE) framework. Specifically, time zero (ie, treatment initiation) was clearly defined, consistent eligibility criteria were applied at baseline, follow‐up and outcome assessment periods were prespecified, and a detailed protocol and statistical analysis plan guided the conduct of the study. Propensity score matching was used to address confounding and enhance comparability between treatment groups, aligning with best practices outlined for target trial emulation in observational studies.^13^, ^14^

The study population comprised patients aged ≥18 years with an AF diagnosis who were identified during the study period (January 1, 2010, and September 30, 2021) using *International Classification of Diseases, Ninth Revision* and *Tenth Revision* (*ICD‐9* and *ICD‐10*, respectively) codes. The date of first prescription claim for dronedarone (800 mg/day) or amiodarone (≥100 mg/day) on or after the AF diagnosis date was considered their first AAD prescription and served as the index date (Figure 1). All patients were required to have ≥12 months of continuous enrollment during the baseline period before the index date. Patients were excluded if they had a history of decompensated HF (stage IV), maze procedure for AF, or received any AAD any time before the index date. Additionally, patients with permanent AF during the 12 months before the index date were excluded. Lastly, patients who underwent cardiac surgery or ablation within a 3‐month period before the index date or used digoxin in the 30 days before the index date were also excluded.

An on‐treatment approach based on the index treatment patients received was used and AEs that occurred during the treatment period, along with events occurring during the 9‐month period after treatment discontinuation, were included in the analysis. Treatment discontinuation was defined as a gap >30 days between the run‐out date (ie, fill date plus days supplied) of the current prescription and the fill date of the next AAD prescription, or a switch or addition of a different AAD. Patients were followed from index date until AAD switch, AAD augmentation (defined as a prescription for a different AAD that commenced while on the index therapy, provided that the duration of overlap was ≥30 days), health plan disenrollment, death, or study end (December 31, 2021), whichever came first. Clinical and economic outcomes were assessed in matched cohorts during the follow‐up period time post index.

### Clinical Outcomes

Clinical outcomes included AEs associated with dronedarone and amiodarone, AEs that were frequently listed in the FDA Adverse Event Reporting System,^15^ and all of the AEs included in the FDA labels.^16^, ^17^ These AEs were classified into the following categories: (1) any safety event of any category; (2) diseases of the respiratory system; (3) cardiac and vascular disorders; (4) general disorders, symptoms, signs, abnormal clinical and laboratory investigations; (5) endocrine, nutritional, and metabolic diseases; (6) diseases of the digestive system/gastrointestinal/hepatobiliary system; (7) nervous system and psychiatric disorders; (8) diseases of the musculoskeletal system and connective tissue; (9) diseases of the genitourinary system (renal); (10) diseases of the blood and blood‐forming organs and certain disorders involving the immune mechanism; (11) diseases of the eye and adnexa; and (12) diseases of the skin and subcutaneous tissue. Evidence of the specified condition in inpatient, outpatient, or emergency department (ED) claims or a claim with a Common Procedural Terminology code and a relevant thyroid, hepatic, pulmonary, or visual function test during the specified study period signified an AE (Tables S1 and S2).

### Economic Outcomes

Economic outcomes included all‐cause and cardiovascular‐related HCRU and corresponding costs incurred by the payors. HCRU was categorized into broad categories as follows: inpatient, ED, primary care physician visits, and specialist visits (Table S3). Total medical cost was reported as the sum of costs in each setting and was inflation adjusted to 2021 US dollars using the medical component of the Consumer Price Index.^18^, ^19^, ^20^

### Statistical Analysis

Patients meeting the study criteria were selected for 1:1 propensity score matching. To create similar cohorts of dronedarone and amiodarone users with the same covariate distribution, patients were matched by demographics, baseline comorbidities, medical history, and concomitant medications based on greedy matching with a caliper of 0.1. Specific variables accounted for in the propensity score matching algorithm included age at index, sex, race, geographic region, health plan type, year of AF diagnosis, setting of first AF diagnosis, type of AF (paroxysmal or persistent), atrial flutter, cardiomyopathy or congenital anomalies of the heart, chronic obstructive pulmonary disease, chronic renal disease, congestive HF, diabetes, hypertension, ischemic heart disease, myocardial infarction, peripheral artery disease, stroke or transient ischemic attack, valvular disease, sleep apnea, Charlson Comorbidity Index score (Table S4), CHA_2_DS_2_‐VASc score (Table S5), vitamin K antagonists, warfarin, P2Y12 inhibitors, acetylsalicylic acid, direct acting oral anticoagulants, beta blockers, calcium channel blockers (nondihydropyridine and dihydropyridine), digoxin, statins, ezetimibe, proprotein convertase subtilisin/kexin type 9 inhibitors, sodium‐glucose cotransporter‐2 inhibitors, pulmonary medications, time (in days) between the first diagnosis of AF and the index date, and baseline procedures (cardioversion, catheter ablation, implantable cardioverter‐defibrillator insertion, and pacemaker insertion). Standardized mean differences were calculated for each covariate. The codes used to identify medications during the baseline period are listed in Table S6.

For the analysis of each AE, patients with evidence of such AE at baseline were excluded from the corresponding analysis. For example, patients with existing pulmonary toxicity at baseline were excluded from analysis of pulmonary toxicity risk during the follow‐up period. Generalized linear models with Poisson distribution were used to compare the event rate ratios (RR) and incidence RRs along with corresponding CIs of the AEs between the propensity score‐matched cohorts. HCRU was calculated as the total number of events (hospitalizations, outpatient visits) divided by patient‐years at risk during the follow‐up period. Direct medical costs were adjusted for inflation to the 2023 US dollar using the annual medical care component of the Consumer Price Index. Annualized HCRU and per‐patient‐per‐year costs (including outpatient care, ED inpatient care, and pharmacy) were assessed to account for differential lengths of baseline and follow‐up. The data were processed and analyzed using Python Software Foundation. Python Language Reference, version 3.13. Available at http://www.python.org.

To assess the likelihood of unmeasured/residual confounding in this study, we used the negative control outcome (NCO) technique.^21^ We selected a composite NCO comprising urinary tract infection (UTI), knee replacement surgery, and hemorrhoids (not expected to be different in these groups). To further examine the magnitude of unmeasured confounding, we calculated E values.

### Sensitivity Analyses

Several sensitivity analyses were conducted. First, all outcomes were assessed using an intention‐to‐treat perspective during the period starting from the fill date of the first AAD prescription until health plan disenrollment, death, or study end, whichever occurred first. Event attributions were based on the initial AAD that patients received. A second sensitivity analysis assessed the impact of having ≥2 sequential prescriptions of the same AAD (eg, ≥2 prescriptions of dronedarone without a prescription for amiodarone or another AAD in between) on the study outcomes. A third sensitivity analysis examined outcomes in a subset of patients with and without congestive HF stages I, II, and III. A final sensitivity analysis assessed outcomes in patients who initiated dronedarone or amiodarone within 90 days from index AF diagnosis.

## RESULTS

### Cohort Formation

**Figure 2:**
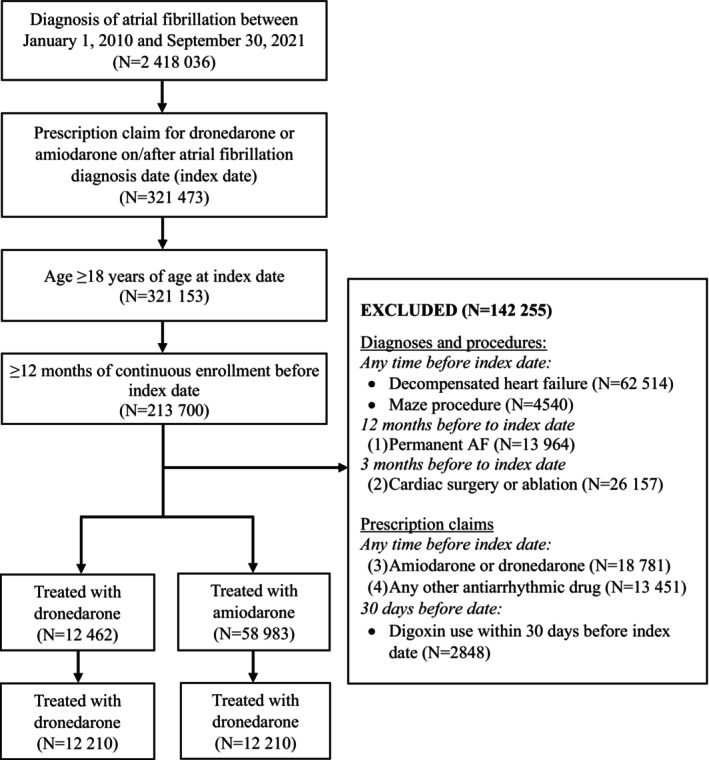
Patient attrition. AF indicates atrial fibrillation.

Among the 2 418 036 patients who received their first‐ever AF diagnosis in any position on the claim in the inpatient or outpatient setting from January 1, 2010 to September 30, 2021, the study eligibility criteria were met by 12 462 patients prescribed dronedarone and 58 983 patients prescribed amiodarone (Figure 2). After propensity score estimation and 1:1 matching, the overall study population included 24 420 patients (12 210 in each cohort).

### Patient Characteristics

In the postmatched cohorts, the treatment groups were well balanced for all demographic and baseline characteristics used as covariates in the propensity scoring model as assessed by standardized differences (standardized differences of adjusted baseline characteristics <0.1; Table 1). Patients had a mean age of 70 years and approximately 45% were female. The majority of patients had a high‐risk CHA_2_DS_2_‐VASc score (scores 2–9: 82.5% and 84.5% for dronedarone and amiodarone, respectively) and more than half had a Charlson Comorbidity Index score≥1.

**Table 1:** Demographic and Baseline Characteristics in the Unmatched and Matched Treatment Cohorts

Characteristics	Unmatched				Standardized difference	Matched				Standardized difference
	Dronedarone (N=12 462)		Amiodarone (N=58 983)			Dronedarone (N=12 210)		Amiodarone (N=12 210)		
	No.	%	No.	%		No.	%	No.	%	
Age (continuous), y, mean±SD	69.6 (10.7)		74.6 (9.8)		0.0473	69.9 (10.5)		70.3 (11.2)		0.0034
Sex, female	5492	44.1	26 504	44.9	0.0161	5409	44.3	5489	45.0	0.0141
Race or ethnicity										
White	9954	79.9	44 762	75.9	0.0965	9733	79.7	9785	80.1	0.0100
Black	872	7.0	5185	8.8	0.0668	864	7.1	871	7.1	0.0000
Hispanic	799	6.4	5001	8.5	0.0800	792	6.5	775	6.3	0.0082
Asian	306	2.5	1225	2.1	0.0267	302	2.5	281	2.3	0.0131
Unknown	531	4.3	2810	4.8	0.0240	519	4.3	498	4.1	0.0100
Geographic region										
Northeast	1200	9.6	4277	7.3	0.0828	1159	9.5	1137	9.3	0.0069
North central	2359	18.9	11 837	20.1	0.0303	2322	19.0	2323	19.0	0.0000
South	6240	50.1	25 137	42.6	0.1508	6073	49.7	5976	48.9	0.0160
West	2652	21.3	17 688	30.0	0.2002	2645	21.7	2763	22.6	0.0217
Unknown	4		10		0.0000	4		3		0.0000
Atrial fibrillation diagnosis setting										
Inpatient	3053	24.5	20 432	34.6	0.2227	3028	24.8	2978	24.4	0.0093
Outpatient	9409	75.5	38 551	65.4	0.2227	9182	75.2	9232	75.6	0.0093
Charlson Comorbidity Index, categorical										
0	5435	43.6	14 124	23.9	0.4260	5226	42.8	4990	40.9	0.0385
1–2	4360	35.0	20 878	35.4	0.0084	4321	35.4	4558	37.3	0.0395
3–4	1958	15.7	15 184	25.7	0.2487	1954	16.0	1926	15.8	0.0055
5+	709	5.7	8797	14.9	0.3062	709	5.8	736	6.0	0.0085
Comorbidities										
Atrial flutter	3028	24.3	15 343	26.0	0.0392	2986	24.5	3011	24.7	0.0046
Obesity	1272	10.2	9370	15.9	0.1698	1270	10.4	1498	12.3	0.0599
Cardiomyopathy/congenital anomaly of heart	2035	16.3	13 545	23.0	0.1692	2026	16.6	2060	16.9	0.0080
Chronic obstructive pulmonary disease	2450	19.7	17 700	30.0	0.2401	2441	20.0	2472	20.2	0.0050
Chronic renal disease	1097	8.8	6995	11.9	0.1019	1093	9.0	1127	9.2	0.0070
Congestive heart failure	1659	13.3	14 727	25.0	0.3007	1656	13.6	1700	13.9	0.0087
Diabetes	2143	17.2	16 586	28.1	0.2626	2139	17.5	2211	18.1	0.0157
Hypertension	10 260	82.3	51 361	87.1	0.1336	10 093	82.7	10 152	83.1	0.0106
Hypercholesterolemia	2476	19.9	7256	12.3	0.2079	2403	19.7	2182	17.9	0.0461
Ischemic heart disease	3265	26.2	13 431	22.8	0.0791	3200	26.2	3205	26.2	0.0000
Myocardial infarction	1213	9.7	10 233	17.3	0.2238	1211	9.9	1209	9.9	0.0000
Peripheral artery disease	960	7.7	8788	14.9	0.2289	957	7.8	977	8.0	0.0074
Stroke or transient ischemic attack	1196	9.6	8435	14.3	0.1453	1190	9.7	1187	9.7	0.0000
Valvular disease	3998	32.1	19 926	33.8	0.0362	3904	32.0	3903	32.0	0.0000
Sleep apnea	2190	17.6	8902	15.1	0.0676	2138	17.5	2070	17.0	0.0132
CHA2DS2‐VASc score, categorical										
Low risk (score: 0)	573	4.6	818	1.4	0.1884	517	4.2	480	3.9	0.0152
Intermediate risk (score: 1)	1725	13.8	3019	5.1	0.3008	1615	13.2	1412	11.6	0.0486
High risk (score: 2–9)	10 164	81.6	55 146	93.5	0.3664	10 078	82.5	10 318	84.5	0.0539
Baseline procedures										
Cardioversion	994	0.1	5645	0.1	0.0565	979	0.1	977	0.1	0.0000
Catheter ablation	25	0.0	149	0.0	0.0200	25	0.0	27	0.0	0.0000
Implantable cardioverter‐defibrillator insertion	13	0.0	567	0.0	0.1219	13	0.0	87	0.01	0.0952
Pacemaker insertion	256	2.1	1518	2.6	0.0330	254	2.1	236	1.9	0.0143
Medications										
Warfarin	2703	21.7	8256	14.0	0.2021	2615	21.4	2595	21.3	0.0024
Aspirin	124	1.0	508	0.90	0.0103	122	1.0	120	1.0	0.0000
Direct oral anticoagulants	3767	30.2	13 714	23.3	0.1564	3687	30.2	3751	30.7	0.0109
Rate controlling	9952	79.9	43 846	74.3	0.1336	9726	79.7	9711	79.5	0.0050
Digoxin	523	4.2	2054	3.5	0.0364	511	4.2	509	4.2	0.0000
Antihypertensives	7627	61.2	39 922	67.7	0.1361	7505	61.5	7969	65.3	0.0789

### Clinical Outcomes

Event rates of each category of AEs were generally lower with dronedarone than with amiodarone during the follow‐up period. For instance, the event RR for any respiratory AEs was 0.65 (95% CI, 0.63–0.66), any cardiac and vascular AEs was 0.71 (95% CI, 0.69–0.72), and any gastrointestinal/hepatobiliary AEs was 0.81 (95% CI, 0.79–0.84) (Table 2). Furthermore, the event RRs for most individual AEs were lower in the dronedarone‐treated cohort compared with the amiodarone‐treated cohort with the exception of pneumonitis (5.01 [95% CI, 2.63–9.57]), gastrointestinal hemorrhage (1.05 [95% CI, 1.00–1.09]), liver injury (5.17 [95% CI, 1.77–15.06]), myalgia (1.33 [95% CI, 1.23–1.44]), and pruritus (1.17 [95% CI, 1.02–1.34]). Although numerically higher rates of liver injury and gastrointestinal hemorrhage were observed with dronedarone, these findings were based on small event counts, were associated with wide CIs, or were not statistically significant. Overall, the gastrointestinal/hepatobiliary composite outcomes favored dronedarone over amiodarone.

**Table 2:** Event Rates of Adverse Events During the Follow‐Up Period

Event	Dronedarone (N=12 210)			Amiodarone (N=12 210)			Dronedarone vs amiodarone
	No.	Patient‐time at risk, y	Rate (per 100 patient‐y) (95% CI)	No.	Patient‐time at risk, y	Rate (per 100 patient‐y) (95% CI)	RR (95% CI)
Any safety event of any category	16 710	9843	169.8 (167.2–172.4)	20 423	9460	215.9 (212.9–218.9)	0.78 (0.77–0.80)
Any respiratory event	16 928	6471	261.61 (257.7–265.6)	23 181	5737	404.0 (398.9–409.3)	0.65 (0.63–0.66)
Any cardiac and vascular event	10 433	3263	319.7 (313.6–325.9)	15 569	3451	451.2 (444.1–458.3)	0.71 (0.69–0.72)
Any general event	16 710	9843	169.8 (167.2–172.4)	20 423	9460	215.9 (212.9–218.9)	0.78 (0.77–0.80)
Any endocrine event	3537	12 186	29.0 (28.1–30.0)	9570	12 419	77.1 (75.5–78.6)	0.38 (0.36–0.39)
Any gastrointestinal/hepatobiliary event	9402	12 427	75.7 (74.1–77.2)	10 894	11 736	92.8 (91.1–94.6)	0.81 (0.79–0.84)
Any neurological event	7400	12 123	61.0 (59.7–62.5)	9739	11 760	82.8 (81.2–84.5)	0.74 (0.71–0.76)
Any musculoskeletal event	10 228	14 718	69.5 (68.2–70.9)	16 404	14 233	115.3 (113.5–117.0)	0.60 (0.59–0.62)
Any renal and urinary disorders	9901	14 124	70.1 (68.7–71.5)	16 631	13 444	123.7 (121.8–125.6)	0.57 (0.55–0.58)
Any blood‐related event	7261	15 110	48.1 (47.0–49.2)	8990	14 484	62.1 (60.8–63.4)	0.77 (0.75–0.80)
Any ocular event	1598	15 700	10.2 (9.7–10.7)	2610	15 487	16.9 (16.2–17.5)	0.60 (0.57–0.64)
Any dermatological event	1381	16 061	8.6 (8.2–9.1)	1310	15 884	8.3 (7.8–8.7)	1.04 (0.97–1.12)

RR indicates rate ratio.

Similarly, the incidence rates of each category of AEs were generally lower with dronedarone than with amiodarone. For instance, the incidence RR was 0.65 (95% CI, 0.61–0.69) for any respiratory AEs, 0.80 (95% CI, 0.75–0.86) for any cardiac and vascular AEs, and 0.90 (95% CI, 0.85–0.95) for any gastrointestinal/hepatobiliary AEs (Table 3). The incidence rates for most individual AEs were lower in the dronedarone‐treated cohort compared with the amiodarone‐treated cohort except for pruritus (1.2; [95% CI, 1.01–1.48]). For a complete list of incidence RR and event RRs of AEs, please refer to Tables S7 through S22.

**Table 3:** Incidence Rate Ratios of Adverse Events During the Follow‐Up Period

Event	Dronedarone (N=12 210)			Amiodarone (N=12 210)			Dronedarone vs Amiodarone
	No.	Patient‐time at risk, y	Rate (per 100 patient‐y) (95% CI)	No.	Patient‐time at risk, y	Rate (per 100 patient‐y) (95% CI)	IRR (95% CI)
Any safety event of any category	463	295	156.9 (143.0–171.9)	560	288	194.7 (178.9–211.5)	0.81 (0.71–0.91)
Any respiratory event	2135	4472	47.8 (45.7–49.8)	2511	3421	73.4 (70.6–76.3)	0.65 (0.61–0.69)
Any cardiac and vascular event	1622	1690	96.0 (91.4–100.8)	1931	1614	119.6 (114.3–125.1)	0.80 (0.75–0.86)
Any general event	2865	7266	39.4 (38.0–40.9)	3370	6549	51.5 (49.7–53.2)	0.77 (0.73–0.81)
Any endocrine event	1115	11 189	10.0 (9.4–10.6)	2054	10 624	19.3 (18.5–20.2)	0.52 (0.48–0.55)
Any gastrointestinal/hepatobiliary event	2422	10 258	23.6 (22.7–24.6)	2536	9677	26.2 (25.2–27.3)	0.90 (0.85–0.95)
Any neurological event	1943	10 499	18.5 (17.7–19.4)	2296	9898	23.2 (22.3–24.2)	0.80 (0.75–0.85)
Any musculoskeletal event	1513	13 415	11.3 (10.7–11.9)	2034	12 504	16.3 (15.6–17.0)	0.69 (0.65–0.74)
Any renal and urinary disorders	1453	12 846	11.3 (10.7–11.9)	2001	11 760	17.0 (16.3–17.8)	0.66 (0.62–0.71)
Any blood‐related event	1337	13 927	9.6 (9.1–10.1)	1666	13 102	12.7 (12.1–13.3)	0.76 (0.70–0.81)
Any ocular event	790	14 951	5.3 (4.9–5.7)	868	14 714	5.9 (5.5–6.3)	0.90 (0.81–0.99)
Any dermatological event	774	15 346	5.0 (4.7–5.4)	709	15 259	4.7 (4.3–5.0)	1.09 (0.98–1.20)

IRR indicates incidence rate ratio.

### Economic Outcomes

All‐cause HCRU was lower in the dronedarone cohort than in the amiodarone cohort. Although the RR of 30‐day readmission was higher in the dronedarone cohort than in the amiodarone cohort (1.29 [95% CI, 1.01–1.66]; *P*=0.045), the incidence of inpatient hospitalization at any point after the index date was significantly lower in the dronedarone cohort than in the amiodarone cohort (0.75 [95% CI, 0.72–0.78]; *P*<0.001). The incidence rates of primary care physician, specialist, and ED visits were significantly lower in the dronedarone cohort than in the amiodarone cohort. Similarly, the event rates of inpatient hospitalization (0.69 [95% CI, 0.67–0.71]; *P*<0.001) and outpatient visits (0.87 [95% CI, 0.87–0.87]; *P*<0.001) were significantly lower in the dronedarone cohort than in the amiodarone cohort (Table 4).

**Table 4:** All‐Cause and Cardiovascular‐Related Health Care Resource Use During the Follow‐Up Period

Variable	Dronedarone (N=12 210)			Amiodarone (N=12 210)			Dronedarone vs amiodarone	
	No.	Patient‐time, y	Rate (per 100 patient‐y) (95% CI)	No.	Patient‐time, y	Rate (per 100 patient‐y) (95% CI)	Rate ratio (95% CI)	*P* value
All‐cause HCRU								
Inpatient hospitalizations	3814	2351	162.2 (157.1–167.5)	4371	2014	217.1 (210.7–223.6)	0.75 (0.72–0.78)	<0.001
30‐day readmission after first AF hospitalization post index	140	5269	2.7 (2.2–3.1)	109	5299	2.1 (1.69–2.5)	1.29 (1.01–1.66)	0.045
Outpatient visits								
PCP	11 113	2335	475.9 (467.1–484.8)	11 174	1836	608.7 (597.4–620.1)	0.78 (0.76–0.81)	<0.001
Specialist	11 668	1033	1129.7 (1109.2–1150.3)	11 558	1017	1136.5 (1115.9–1157.4)	0.99 (0.97–1.02)	0.672
ED	4425	10 723	41.3 (40.1–42.5)	4865	9027	53.9 (52.4–55.4)	0.77 (0.74–0.80)	<0.001
Event rates of all‐cause HCRU								
Inpatient hospitalizations	9837	14 833	66.3 (65.0–67.6)	12 595	13 153	95.8 (94.1–97.5)	0.69 (0.67–0.71)	<0.001
Outpatient visits	425 247	14 833	2866.9 (2858.4–2875.6)	433 619	13 153	3296.8 (3287.0–3306.6)	0.87 (0.87–0.87)	<0.001
PCP	139 563	14 833	940.9 (936.0–945.9)	155 845	13 153	1184.9 (1179.0–1190.8)	0.79 (0.79–0.80)	<0.001
Specialist	224 066	14 833	1510.6 (1504.4–1516.9)	216 907	13 153	1649.1 (1642.2–1656.1)	0.92 (0.91–0.92)	<0.001
ED	13 084	14 833	88.2 (86.7–89.7)	13 998	13 153	106.4 (104.7–108.20)	0.83 (0.81–0.85)	<0.001
Cardiovascular‐related HCRU								
Inpatient hospitalizations								
Any time after index	1625	4249	38.3 (36.4–40.2)	1330	4407	30.2 (28.6–31.9)	1.27 (1.18–1.36)	<0.001
30‐day readmission after first AF hospitalization post‐index	72	5304	1.4 (1.1–1.7)	51	5334	0.96 (0.71–1.3)	1.42 (0.99–2.03)	0.055
Outpatient visits								
PCP	6434	7952	80.9 (78.9–82.9)	6524	6525	99.9 (97.6–102.5)	0.81 (0.78–0.84)	<0.001
Specialist	10 464	2854	366.6 (359.6–373.7)	9812	3109	315.6 (309.4–321.9)	1.16 (1.13–1.19)	<0.001
ED	1359	13 752	9.9 (9.4–10.4)	1332	12 164	10.9 (10.4–11.6)	0.90 (0.84–0.97)	0.008
Event rates of cardiovascular‐related HCRU								
Inpatient hospitalizations	2596	14 833	17.5 (16.8–18.2)	2190	13 153	16.7 (16.0–17.4)	1.05 (0.99–1.11)	0.084
Outpatient visits	111 208	14 833	749.8 (745.4–754.2)	103 539	13 153	787.2 (782.4–792.0)	0.95 (0.94–0.96)	<0.001
PCP	31 363	14 833	211.5 (209.1–213.8)	32 221	13 153	244.97 (242.3–247.7)	0.86 (0.85–0.88)	<0.001
Specialist	71 336	14 832	480.9 (477.4–484.5)	61 386	13 153	466.7 (463.0–470.4)	1.03 (1.02–1.04)	<0.001
ED	1896	14 833	12.8 (12.2–13.4)	1814	13 153	13.8 (13.2–14.4)	0.93 (0.87–0.99)	0.021
Other outpatient	14 790	14 833	99.7 (98.1–101.3)	15 865	13 153	120.6 (118.8–122.5)	0.83 (0.81–0.85)	<0.001

AF indicates atrial fibrillation; ED, emergency department; HCRU, health care resource use; and PCP, primary care physician.

For cardiovascular‐related HCRU, compared with patients in the amiodarone cohort, patients in the dronedarone cohort had higher incidence rates of inpatient hospitalizations at any time (1.27 [95% CI, 1.18–1.36]; *P*<0.001) and 30‐day readmission after index hospitalization (1.42 [95% CI, 0.99–2.03]; *P*=0.055). However, patients in the dronedarone cohort had fewer primary care physician (*P*<0.001) and ED visits (*P*<0.008) but more specialist outpatient visits (*P*<0.001) than those in the amiodarone cohort. There was no statistical difference in event rates of inpatient hospitalization (1.05 [95% CI, 0.99–1.11]; *P*=0.084) but patients in the dronedarone cohort had lower outpatient visits (*P*<0.001) (Table 4).

The mean annualized costs for all‐cause inpatient visits and primary care physician visits were lower in the dronedarone cohort than in the amiodarone cohort (health care costs per patient per year for all‐cause inpatient visits: $11 952 [SD=55 452] versus $15 935 [SD=53 004], respectively). The cardiovascular‐related health care costs of patients assessed during follow‐up were marginally higher in the dronedarone cohort compared with the amiodarone cohort (3126 [SD=16 744] versus 3011 [SD=24 355]) (Table 5).

**Table 5:** Mean Annualized All‐Cause and Cardiovascular‐Related Health Care Costs During the Follow‐Up Period

	Dronedarone	Amiodarone
All‐cause		
Inpatient visits	11 952 (55452)	15 935 (53004)
PCP visits	3804 (14436)	4580 (15732)
Specialist visits	11 487 (25456)	11 237 (27984)
ED visits	708 (2071)	891 (2423)
cardiovascular‐related		
Inpatient visits	3126 (16744)	3011 (24355)
PCP visits	698 (5142)	706 (4120)
Specialist visits	3163 (5869)	2365 (4768)
ED visits	107 (498)	109 (563)

Data are mean (SD) in US dollars. ED indicates emergency department; and PCP, primary care physician.

### Negative Controls Analysis

Our analysis revealed a statistically significant lower risk of the composite NCO in the dronedarone cohort compared with the amiodarone cohort (incidence RR, 0.82 [95% CI, 0.78–0.87], *P*<0.001). This association was primarily driven by a much higher incidence of UTIs compared with those of knee replacement and hemorrhoids in both cohorts (Table 6). Similar findings were observed when we estimated event rates and event rate ratios of composite and individual NCO conditions (Table 7).

**Table 6:** Incidence of Negative Control Outcomes During the Follow‐Up Period

NCO	Dronedarone (N=12 210)			Amiodarone (N=12 210)			Dronedarone vs Amiodarone	
	No.	Patient‐time at risk, y	Rate (per 100 patient‐y) (95% CI)	No.	Patient‐time at risk, y	Rate (per 100 patient‐y) (95% CI)	IRR (95% CI)	*P* value
Composite NCO	2950	11 565	25.5 (24.6–26.5)	3157	10 189	31.0 (29.9–32.1)	0.82 (0.78–0.87)	<0.001
Urinary tract infection	2162	12 386	17.5 (16.7–18.2)	2507	10 721	23.4 (22.5–24.3)	0.75 (0.71–0.79)	<0.001
Knee replacement	162	14 231	1.1 (1.0–1.3)	150	12 713	1.2 (1.0–1.4)	0.97 (0.77–1.21)	0.754
Hemorrhoids	901	13 491	6.7 (6.3–7.1)	760	12 236	6.2 (5.8–6.7)	1.08 (0.98–1.18)	0.139

IRR indicates incidence rate ratio; NCO, negative control outcome; and UTI.

**Table 7:** Event Rates of Negative Control Outcomes During the Follow‐Up Period

NCO	Dronedarone (N=12 210)			Amiodarone (N=12 210)			Dronedarone vs Amiodarone	
	No.	Patient‐time at risk, y	Rate (per 100 patient‐y) (95% CI)	No.	Patient‐time at risk, y	Rate (per 100 patient‐y) (95% CI)	RR (95% CI)	*P* value
Composite NCO	10 978	14 404	76.2 (74.8–77.7)	14 510	12 848	112.9 (111.1–114.8)	0.68 (0.66–0.69)	<0.001
Urinary tract infection	9194	14 404	63.8 (62.5–65.2)	12 790	12 848	99.6 (97.8–101.3)	0.64 (0.62–0.66)	<0.001
Knee replacement	309	14 404	2.2 (1.9–2.4)	278	12 848	2.2 (1.9–2.4)	0.99 (0.84–1.17)	0.920
Hemorrhoids	1475	14 404	10.2 (9.7–10.8)	1442	12 848	11.2 (10.7–11.8)	0.91 (0.85–0.98)	0.014

NCO indicates negative control outcome; and RR, rate ratio.

In an attempt to explain the unexpected observed association between the composite NCO (and primarily UTI) and amiodarone, we ran several iterations of regression models (1) blanking UTIs that occurred during the first 3 months of follow‐up; (2) blanking UTIs that occurred within 30 days after any hospital admission; (3) adjusting for baseline UTI, diuretic use, and any cancer at index; (4) adjusting for baseline UTI, diuretic use, any cancer, and frailty score^22^, ^23^ at index; and (5) adjusting for variables with standardized difference>0.05 (time from index to AAD initiation) at baseline. However, these analyses did not explain the association between the NCO and amiodarone.

### Sensitivity Analyses

We conducted several sensitivity analyses to validate our results. For the most part, patients on dronedarone were less likely to experience AEs compared with those on amiodarone with few exceptions for individual AEs. The first sensitivity analysis included all patients who met the selection criteria with an intention‐to‐treat approach. Findings from this analysis demonstrated that dronedarone‐treated patients experienced fewer AEs than the amiodarone‐treated patients, with the exception for these specific AEs: tachycardia, ventricular tachycardia, liver injury (although the sample size is small), and myalgia (Tables S23 through S38).

In patients with ≥2 consecutive prescriptions of the same study drug, dronedarone‐treated patients experienced lower event rates of all AE categories (Tables S39 through S54). Among patients with congestive HF, the number of patients meeting the selection criteria was too small to allow for meaningful comparisons between the 2 cohorts (Table S55 through S70). In patients without congestive HF, RRs of AEs were consistently lower in the dronedarone cohort compared with the amiodarone cohort, except for ventricular tachycardia, gastrointestinal hemorrhage, thyroid disorder, diarrhea, abdominal pain, myalgia, and anemia (Tables S71 through S86). Lastly, in patients who initiated dronedarone or amiodarone within 90 days from index AF diagnosis, the event rates, for the most part, were higher with amiodarone, with the exception of ventricular tachycardia, gastrointestinal hemorrhage, decreased appetite, and rhabdomyolysis (Tables S87 through S102).

## DISCUSSION

There are several important conclusions from this analysis, believed to be the largest, most rigorous, head‐to‐head comparison of AEs and HCRU between dronedarone and amiodarone using real‐world data. We observed lower event rates and incidence rates of AEs during follow‐up among patients treated with dronedarone compared with amiodarone. In general, incidence and event rates of all‐cause HCRU were lower in the dronedarone cohort compared with the amiodarone cohort, with the exception for 30‐day readmission rates, which were higher for dronedarone compared with amiodarone. As for cardiovascular‐related HCRU, the risk of inpatient hospitalization and 30‐day readmission was higher in the dronedarone cohort compared with the amiodarone cohort. The number of cardiovascular‐related outpatient visits was lower for the dronedarone cohort compared with the amiodarone cohort. As for the event rates, there was no statistical difference in inpatient hospitalization and ED visits, but patients in the dronedarone cohort had fewer outpatient visits (primary care physician, specialist, and other outpatient visits).

A major finding from this study is evidence for lower incidence and event rates of AEs with dronedarone relative to amiodarone. Recent safety studies and clinical trials have demonstrated similar findings concerning the safety profile of dronedarone. A meta‐analysis by Freemantle et al. found a reduced risk of serious AEs with dronedarone compared with amiodarone.^24^ In the DIONYSOS (Efficacy & Safety of Dronedarone Versus Amiodarone for the Maintenance of Sinus Rhythm in Patients With Atrial Fibrillation) trial, dronedarone demonstrated a better safety profile mainly driven by fewer thyroid, neurologic, skin, and ocular events in the dronedarone group compared with amiodarone.^25^ The present data confirm and extend these findings in clinical practice.

We found that when examining each AE category (event rates) separately, the safety profile favored dronedarone. These results are consistent with the previous literature. Several studies have demonstrated the effect of dronedarone in reducing the risk of liver disease compared with sotalol^26^ and stroke and myocardial infarction compared with other AADs.^27^ In another study, dronedarone was found to reduce the risk of stroke and bleeding events, and a 10‐fold decrease in the risk of interstitial liver disease.^28^ Other studies have reported lower risks of pulmonary and hepatic AEs, such as interstitial lung disease and acute liver injury, in patients receiving dronedarone compared with amiodarone.^24^, ^26^, ^27^, ^29^, ^30^ Moreover, no cases of interstitial lung disease or pulmonary toxicity were reported in dronedarone‐treated patients in the ANDROMEDA (European Trial of Dronedarone in Moderate to Severe Congestive Heart Failure), ADONIS (American‐Australian‐African Trial With Dronedarone in Patients With Atrial Fibrillation or Atrial Flutter for the Maintenance of Sinus Rhythm), and EURIDIS (European Trial in Atrial Fibrillation or Flutter Patients Receiving Dronedarone for the Maintenance of Sinus Rhythm) trials.^31^, ^32^ Contrary to previous studies in which gastrointestinal AEs were higher among dronedarone‐treated patients,^25^, ^33^ our study found lower rates of gastrointestinal AEs with dronedarone compared with amiodarone. Contrary to the evidence in the literature, our study found higher incidence rates and RRs for liver injury; however, the number of events was small (11 and 4 patients in the dronedarone and amiodarone cohorts, respectively).

Our study extends the observation that dronedarone‐treated patients experience lower rates of all‐cause hospitalization compared with amiodarone‐treated patients. However, patients in the dronedarone cohort reported higher rates of cardiovascular‐related inpatient hospitalization and 30‐day readmission compared with patients in the amiodarone cohort. This finding contradicts results from previous safety studies and randomized controlled trials, where dronedarone use significantly decreased the risk of cardiovascular‐related hospitalizations.^34^, ^35^, ^36^, ^37^ In real‐world claims data, hospitalizations are identified based on administrative coding rather than clinical adjudication, increasing the potential for misclassification, particularly when multiple diagnoses are present. Admissions for cardioversion procedures, more frequently associated with amiodarone use, were not specifically excluded and may have contributed to the observed differences. Additionally, despite propensity score matching, residual confounding may persist, as amiodarone is typically prescribed to older and higher‐risk patients, whereas dronedarone may be preferentially used in more clinically stable individuals. Differences in study populations, cohort selection criteria, and study designs may also explain these discrepancies. Only one other study in the United States, by Brophy et al., using the Truven Health Analytics MarketScan Research database, has reported similar findings.^38^ However, results from our sensitivity analyses conducted in a subset of patients with ≥2 sequential prescriptions of the same AAD showed that dronedarone‐treated patients had a significantly lower event rate of cardiovascular‐related HCRU compared with amiodarone‐treated patients.

Although our findings align closely with results from randomized controlled trials and real‐world safety studies, differences in research methods advise against direct comparisons. This real‐world comparative analysis used data from CDM, whereas randomized controlled trials typically focus on a select group of patients with specific end points and involve data collection under closely monitored conditions. Unlike many studies that used placebo or alternative AADs as controls, our study directly compared dronedarone and amiodarone. Therefore, any comparisons drawn should be interpreted with caution.

This study included a comprehensive list of AEs reported to the FDA’s FDA Adverse Event Reporting System as AEs of dronedarone and amiodarone. Additionally, all potential AEs included in the FDA labels were included. The study team focused on the top 99% of AEs reported to the FDA Adverse Event Reporting System dashboard, identifying select AEs using *ICD‐9‐Clinical Modification* (*CM*)/*ICD‐10‐CM* diagnosis codes in the medical claims.

One of the benefits of observational studies using large databases is the ability to identify and track cohorts of real‐world patients. Therefore, the population of patients identified is generalizable to adults diagnosed with AF who are insured by commercial payors or retirees with Medicare Advantage insurance. Still, it is important to acknowledge the limitations of this specific study, which include the following. Amiodarone is frequently initially administered in the inpatient setting. Therefore, the time to amiodarone initiation following AF diagnosis may be biased upward. Although propensity score matching accounted for a broad set of clinical covariates and therapeutic classes, we were unable to adjust for specific concomitant medications, dosing regimens, or potential drug–drug interactions. Given amiodarone’s well‐known interaction profile, unmeasured confounding from these factors may have influenced the observed associations. In addition, patients who were administered amiodarone during the hospitalization and then received dronedarone after discharge were misclassified into the dronedarone cohort. Caution is required when interpreting results of real‐world observational studies given the lack of randomization and subsequent biases introduced into an observational design comparing different medications. Medication claims indicate that a medication was dispensed; however, there are no data to determine whether the medication was used.

Although propensity score matching reduced baseline differences between treatment groups, the possibility of residual confounding cannot be fully excluded. In particular, the higher baseline clinical risk profile among amiodarone‐treated patients raises the possibility that some observed differences in adverse event rates reflect differences in underlying patient characteristics rather than treatment effects alone. To further assess residual confounding, an NCO analysis^21^ was conducted, with the NCO defined before the analysis. The NCO analysis using the composite end point revealed a statistically significantly higher risk of the NCO in the amiodarone cohort than in the dronedarone cohort. This association was mostly driven by the higher number of patients with UTI compared with the number of patients with knee replacement or hemorrhoids in the follow‐up period. In an attempt to explain the association between the NCO (and primarily UTI) and amiodarone, we ran several iterations of regression models (1) blanking UTIs that occurred during the first 3 months of follow‐up; (2) blanking UTIs that occurred within 30 days after any hospital admission; (3) adjusting for baseline UTI, diuretic use, and any cancer at index; (4) adjusting for baseline UTI, diuretic use, any cancer, and frailty score^22^, ^23^ at index; and (5) adjusting for variables with standardized difference>0.05 (time from index to AAD initiation) at baseline. However, these analyses did not explain the association between the NCO and amiodarone. This finding was likely due to chance, given the multiple additional analyses conducted to explore this association.

## CONCLUSIONS

In this large, real‐world observational study of patients with AF, dronedarone was in general associated with lower event/incidence rates of AEs and HCRU compared with amiodarone.

## Sources of Funding

This study was funded by Sanofi.

## Disclosures

Benjamin A. Steinberg reports research support from Abbott, Boston Scientific, Biosense‐Webster, Sanofi, and AltaThera; and consulting to Sanofi, Boston Scientific, Element Science, Milestone, and AltaThera. Firas Dabbous, Divya Shridharmurthy, Samuel Huse, and Chris Colby are employees of Evidera. Ron Preblick and David S. McKindley are employees of Sanofi and may hold shares and stock options in the company. Jason Rashkin is a former employee of Sanofi and may hold shares and stock options in the company. Jagmeet P. Singh has received consultation fees from Abbott, Biosense Webster, Biotronik Inc, Boston Scientific, Cardiologs Inc, Carelog, CVRx Inc, EBR Inc, Impulse Dynamics, Implicity Inc, Phillips, iRhythm, Medtronic Inc, Medscape Inc, Microport Inc, Orchestra Biomed, VektorMedical, SmartCardia and Sanofi.

## Supporting information

Tables S1–S102
