# Supplementary material for: Clinical and Economic Outcomes of Dronedarone Versus Amiodarone Among Patients With Atrial Fibrillation
Source: J Am Heart Assoc. 2025 Dec 3;15(1):e042178. doi: 10.1161/JAHA.125.042178 (PMC12909043; doi:10.1161/JAHA.125.042178)
Supplement: Supplementary file 1 — Tables S1–S102 [file JAH3-15-e042178-s001.zip › jah370032-sup-0001-supinfo.pdf]

SUPPLEMENTAL MATERIAL

Table S1. Safety Event Definitions

Safety event category	Specific safety event
1. Any safety event of any category	Any of the safety events outlined below
2. Diseases of the respiratory system	Dyspnea, interstitial lung disease, cough, pulmonary fibrosis, pulmonary toxicity, respiratory failure, lung disorder, wheezing, pleural effusion, acute respiratory distress syndrome, pulmonary oedema, dyspnea exertional, pneumonitis, hypoxia
3. Cardiac and vascular disorders	Bradycardia (heart rate decreased), dizziness, tachycardia (heart rate increased), atrial fibrillation, hypotension, electrocardiogram QT prolonged, ventricular tachycardia, cardiac failure, torsades de pointes, gastrointestinal hemorrhage, cardiac arrest, chest pain, ventricular fibrillation, edema peripheral, cardiac failure congestive, arrhythmia, cardiogenic shock, cardio-respiratory arrest
4. General disorders, symptoms, signs, abnormal clinical and investigations	Asthenia, fatigue, weight decreased, weight increased, malaise, edema
5. Endocrine, nutritional and metabolic diseases	Hyperthyroidism, hypothyroidism, thyroid disorder, decreased appetite
6. Diseases of the digestive system/gastrointestinal/ hepatobiliary system	Nausea, vomiting, diarrhea, abdominal pain, liver function test abnormal (to the extent data are available), alanine aminotransferase increased, aspartate aminotransferase increased, liver injury, hepatic failure
7. Nervous system and psychiatric disorders	Syncope, confusional state, insomnia, depression
8. Diseases of the musculoskeletal system and connective tissue	Gait disturbance, rhabdomyolysis, myalgia
9. Diseases of the genitourinary system (renal)	Acute kidney injury, renal failure
10. Diseases of the blood and blood-forming organs and certain disorders involving the immune mechanism	Anemia, thrombocytopenia
11. Disease of the eye and adnexa	Blindness, visual impairment, vision blurred
12. Diseases of the skin and subcutaneous tissue	Rash, pruritus

Table S2. ICD Codes Used to Identify Safety Events. See Excel file.

Table S3. Healthcare Resource Utilization Event Definitions

Measures	Definition
All-cause HCRU	
Hospitalization	Hospitalizations for any cause
Length of stay	Discharge date – admission date + 1
ER visit	ER visits for any cause
Outpatient office visit	Outpatient visits for any cause
30-day readmission after first AF hospitalization post-index	Hospital stays for any cause within 30-days of first AF hospitalization (primary ICD-9/10 diagnosis code related to AF*) post-index
CV-related HCRU	
Hospitalization	Hospitalizations with a primary ICD-9/10 diagnosis code related to AF, atrial tachyarrhythmias, ischemic stroke/TIA, myocardial infarction, ventricular arrhythmias, bradycardia, or heart failure
Length of stay	Discharge date – admission date + 1
ER visit	ER visits with a primary ICD-9/10 diagnosis code related to AF, atrial tachyarrhythmias, ischemic stroke/TIA, myocardial infarction, ventricular arrhythmias, bradycardia, or heart failure
Outpatient office visit	Outpatient visits with an ICD-9/10 diagnosis code related to AF, atrial tachyarrhythmias, ischemic stroke/TIA, myocardial infarction, ventricular arrhythmias, bradycardia, or heart failure

AF ICD-9 code: 427.31; ICD-10 codes: I48.0, I48.1, I48.2, I48.91, I48.11, I48.19, I48.20.

AAD = antiarrhythmic drug; ACS = acute coronary syndrome; AF = atrial fibrillation; CPT = Current Procedural Terminology; CV = cardiovascular; CVD = cardiovascular disease; ER = emergency room; HCRU = healthcare resource utilization; HF = heart failure; ICD-9/10 = International Classification of Diseases, Ninth/Tenth Revision; IS = ischemic stroke; LOS = length of stay; NA = not applicable; PCS = procedure coding system; TIA = transient ischemic attack.

Table S4. Variables and Weights Used to Calculate the Charlson Comorbidity Index

Comorbidity	Weight	ICD-9	ICD-10
Myocardial infarction	0	410.x, 412.x	I21.x, I22.x, I25.2
Congestive heart failure	2	398.91, 402.01, 402.11, 402.91, 404.01, 404.03, 404.11, 404.13, 404.91, 404.93, 425.4–425.9, 428.x	I09.9, I11.0, I13.0, I13.2, I25.5, I42.0, I42.5–I42.9, I43.x, I50.x, P29.0
Peripheral vascular disease	0	093.0, 437.3, 440.x, 441.x, 443.1–443.9, 47.1, 557.1, 557.9, V43.4	I70.x, I71.x, I73.1, I73.8, I73.9, I77.1
Cerebrovascular disease	0	362.34, 430.x–438.x	G45.x, G46.x, H34.0, I60.x–I69.x
Dementia	2	290.x, 294.1, 331.2	F00.x–F03.x, F05.1, G30.x, G31.1
Chronic pulmonary disease	1	416.8, 416.9, 490.x–505.x, 506.4, 508.1, 508.8	I27.8, I27.9, J40.x–J47.x, J60.x–J67.x, J68.4, J70.1, J70.3
Rheumatic disease	1	446.5, 710.0 - 710.4, 714.0 - 714.2, 714.8, 725.x	M05.x, M06.x, M31.5, M32.x - M34.x, M35.1, M35.3, M36.0
Peptic ulcer disease	0	531.x–534.x	K25.x–K28.x
Mild liver disease	2	070.22, 070.23, 070.32, 070.33, 070.44, 070.54, 070.6, 070.9, 570.x, 571.x, 573.3, 573.4, 573.8, 573.9, V42.7	"B18.x, K70.0–K70.3, K70.9, K71.3–K71.5, K71.7, K73.x, K74.x
Diabetes without chronic complication	0	250.0–250.3, 250.8, 250.9	"E10.0, E10.1, E10.6, E10.8, E10.9
Diabetes with chronic complication	1	250.4–250.7	"E10.2–E10.5, E10.7, E11.2–E11.5
Hemiplegia or paraplegia	2	334.1, 342.x, 343.x, 344.0–344.6, 344.9	"G04.1, G11.4, G80.1, G80.2, G81.x
Renal disease	1	403.01, 403.11, 403.91, 404.02, 404.03, 404.12, 404.13, 404.92, 404.93, 582.x, 583.0–583.7, 585.x, 586.x, 588.0, V42.0, V45.1, V56.x	"I12.0, I13.1, N03.2–N03.7, N05.2
Any malignancy, including lymphoma and leukemia, except malignant neoplasm of skin	2	140.x–172.x, 174.x–195.8	"C00.x–C26.x, C30.x–C34.x, C37.x

Moderate or severe liver disease	4	456.0–456.2, 572.2–572.8	"I85.0, I85.9, I86.4, I98.2, K70.4
Metastatic solid tumor	6	196.x–199.x	C77.x–C80.x
AIDS/HIV	4	042.x–044.x	B20.x–B22.x, B24.x

AIDS = acquired immunodeficiency syndrome; HIV = human immunodeficiency virus; ICD-9/10 = International Classification of Diseases, Ninth/Tenth Revision.

Table S5. CHA2DS2-VASc Scoring Algorithm

Criteria		Points
Age	<65 years old	0
	65–74 years old	+1
	≥75 years old	+2
Sex	Male	0
	Female	+1
Congestive heart failure history		+1
Hypertension history		+1
Stroke/TIA/thromboembolism history		+2
Vascular disease history (prior myocardial infraction, peripheral artery disease, or aortic plaque)		+1
Diabetes mellitus history		+1

TIA = transient ischemic attack.

Table S6. Codes Used to Identify Medications Used During the Baseline Period

**Variable**	**Optum Clinformatics Data Mart Definition**
Aldosterone antagonists	Any outpatient prescription claims with the “C03DA” WHO ATC code prefix
Angiotensin II receptor blockers	Any outpatient prescription claims with any of the following WHO ATC code prefixes: C09C, C09D, C10BX10, C10BX16
AngiotensiN=converting-enzyme inhibitor	Any outpatient prescription claims with any of the following WHO ATC code prefixes: C09A, C09B, C10BX04, C10BX06, C10BX07, C10BX11, C10BX12, C10BX13, C10BX14, C10BX15, C10BX17, C10BX18
Angiotensin Receptor-Neprilysin inhibitor	Any outpatient prescription claims with the “C09DX04” WHO ATC code prefix
Beta-blocking agents	Any outpatient prescription claims with the “C07” WHO ATC code prefix (excluding “C07BA07” or “C07FX02” or prescriptions with the generic name “sotalol HCL”)
Calcium-channel blockers, dihydropyridine	Any outpatient prescription claims with any of the following WHO ATC code prefixes: C07FB, C08CA, C09BB (excluding C09BB10), C09DB, C10BX03, C10BX07, C10BX09, C10BX11, C10BX14, or C10BX18
Calcium-channel blockers, noN=dihydropyridine	Any outpatient prescription claims with any of the following WHO ATC code prefixes: C08 (excluding C08CA) or C09BB10
Cardioversion	The occurrence of a medical claim with any of the following diagnosis or procedure codes in any claim line position from the Medical Services table:<br>● ICD-9-CM procedure: 99.61, 99.62<br>● ICD-10-CM procedure: 5A2204Z<br>● CPT: 92960, 92961
Digoxin	Any outpatient prescription claims with the generic name “digoxin” or the “C01AA05” WHO ATC code prefix. Only tablets and liquid formulations will be considered.
Diuretics	Any outpatient prescription claims with any of the following WHO ATC code prefixes: C02L, C03, C07B, C07C, C07D, C08GA, C09BA, C09DA, C10BX13
Eplerenone	Any outpatient prescription claims with the generic name “eplerenone” or the WHO ATC code “C03DA04”

Ezetimibe	Any outpatient prescription claims with the generic name "ezetimibe"
Oral anticoagulants for thromboprophylaxis	The occurrence of any outpatient prescription claims with the following generic names:<br><br>"Warfarin Sodium", "Apixaban", "Edoxaban Tosylate", "Dabigatran Etexilate Mesylate", "Rivaroxaban"
$P2Y_{12}$ agents	Any outpatient prescription claims with the following generic names: "clopidogrel," "ticlopidine," "ticagrelor," or "prasugrel"
Proarrhythmic medications (any)	Any outpatient prescription claim meeting the following criteria (excluding topical administrations):<br><br>● Generic names: antifungals including "fluconazole," "ketoconazole," "itraconazole," or "miconazole"; antihistamines including "terfenadine" or "astemizole"; antimalarials including "quinine," "chloroquine," or "halofantrine"; "tacrolimus"; or "methadone"<br>● WHO ATC code prefixes: N05A or N06C (antipsychotics) or N06AA (tricyclic antidepressants)<br><br>The occurrence of a medical claim with any of the following procedure codes in any claim line position from the Medical Services table:<br><br>● CPT/HCPCS codes: 80152, 80160, 80173, 80174, 80182, 80197, 84228, C9125, C9255, H0020, J0390, J0401, J0780, J1230, J1631, J1790, J3400, J3486, J7508, Q0161, Q0164, Q0174, S0136, S0163, 80178, 83840, C9204, J0400, J1320, J1450, J1630, J1810, J1835, J2358, J2426, J2680, J2794, J2950, J3080, J3230, J3270, J3280, J7507, J7525, Q0165, Q0171, Q0172, S0029, S0096, S0109, S0166, S0183
Proprotein convertase subtilisin/kexin type 9 inhibitor (any, number of prescriptions)	The occurrence of any outpatient prescription claims with the following generic names: "alirocumab" or "evolocumab"
Pulmonary medications	Any outpatient prescription claims with the "R03" WHO ATC code prefix
Sodium-glucose transport protein 2 inhibitors	Any outpatient prescription claims with the WHO ATC prefix "A10BK"
Spironolactone	Any outpatient prescription claims with the following generic names: "spironolactone," "spironolactone/hydrochlorothiazide," "spironolactone micronized"

Statins	Any outpatient prescription claims with the with the following generic names: “simvastatin,” “lovastatin,” “pravastatin,” “fluvastatin,”, “atorvastatin,” “cerivastatin,” “rosuvastatin,” or “pitavastatin”

Table S7. Incidence Rates of Respiratory Adverse Events in Patients Comprised Primary Cohort

	**Dronedarone (N=12,210)**			**Amiodarone (N=12,210)**			**Dronedarone vs Amiodarone**
	N	Patient-time at risk (years)	Rate (Per 100 patient-years) (95% CI)	N	Patient-time at risk (years)	Rate (Per 100 patient-years) (95% CI)	IRR (95% CI)
Any respiratory event	2135	4471	47.8 (45.7–49.8)	2511	3421	73.4 (70.6–76.3)	0.65 (0.61–0.69)
Dyspnea	2362	8143	29.0 (27.9–30.2)	2912	7061	41.2 (39.8–42.8)	0.70 (0.67–0.74)
Interstitial lung	310	16154	1.9 (1.7–2.1)	471	15745	3.0 (2.7–3.3)	0.64 (0.56–0.74)
Cough	1867	11960	15.6 (14.9–16.3)	2108	11627	18.1 (17.4–18.9)	0.86 (0.81–0.92)
Pulmonary fibrosis	138	16553	0.8 (0.7–1.0)	169	16333	1.0 (0.9–1.2)	0.81 (0.64–1.01)
Pulmonary toxicity	224	16270	1.4 (1.2–1.6)	365	15887	2.3 (2.1–2.6)	0.60 (0.51–0.71)
Respiratory failure	961	15537	6.2 (5.8–6.6)	1242	14343	8.7 (8.2–9.2)	0.71 (0.66–0.78)
Lung disorder	1610	10219	15.8 (15.0–16.5)	2055	9949	20.7 (19.8–21.6)	0.76 (0.71–0.81)
Wheezing	388	16087	2.4 (2.2–2.7)	412	15764	2.6 (2.4–2.9)	0.92 (0.80–1.06)
Pleural effusion	1303	14922	8.7 (8.3–9.2)	1696	13195	12.9 (12.3–13.5)	0.68 (0.63–0.73)
Acute respiratory	205	16491	1.2 (1.1–1.4)	317	16072	2.0 (1.8–2.2)	0.63 (0.53–0.75)
Pulmonary edema	684	15883	4.3 (4.0–4.6)	956	15077	6.3 (6.0–6.8)	0.68 (0.62–0.75)
Dyspnea exertional	2368	7170	33.0 (31.7–34.4)	2969	6048	49.1 (47.3–50.9)	0.67 (0.64–0.71)
Pneumonitis	6	16787	0.0 (0.0–0.1)	7	16528	0.0 (0.0–0.1)	0.84 (0.28–2.51)
Hypoxia	536	16090	3.3 (3.1–3.6)	787	15384	5.1 (4.8–5.5)	0.65 (0.58–0.73)

Table S8. Event Rates of Respiratory Adverse Events in Patients Comprised Primary Cohort

	Dronedarone (N=12,210)			Amiodarone (N=12,210)			Dronedarone vs Amiodarone
	N	Patient-time at risk (years)	Rate (Per 100 patient-years) (95% CI)	N	Patient-time at risk (years)	Rate (Per 100 patient-years) (95% CI)	RR (95% CI)
Any respiratory event	16928	6471	261.6 (257.7–265.6)	23181	5737	404.0 (398.9–409.3)	0.65 (0.63–0.66)
Dyspnea	6843	10186	67.2 (65.6–68.8)	8555	9533	89.7 (87.9–91.7)	0.75 (0.72–0.77)
Interstitial lung	717	16425	4.4 (4.1–4.7)	1363	16165	8.4 (8.0–8.9)	0.52 (0.47–0.57)
Cough	3408	13674	24.9 (24.1–25.8)	3842	13427	28.6 (27.7–29.5)	0.87 (0.83–0.91)
Pulmonary fibrosis	494	16690	3.0 (2.7–3.2)	410	16498	2.5 (2.3–2.7)	1.19 (1.04–1.36)
Pulmonary toxicity	576	16483	3.5 (3.2–3.8)	1044	16252	6.4 (6.0–6.8)	0.54 (0.49–0.60)
Respiratory failure	8464	16122	52.5 (51.4–53.6)	10909	15097	72.3 (70.9–73.6)	0.73 (0.70–0.75)
Lung disorder	6539	11658	56.1 (54.7–57.5)	9235	11753	78.6 (77.0–80.2)	0.71 (0.69–0.74)
Wheezing	564	16466	3.4 (3.2–3.7)	584	16136	3.6 (3.3–3.9)	0.94 (0.84–1.06)
Pleural effusion	5802	15731	36.9 (35.9–37.8)	7066	14533	48.6 (47.5–49.8)	0.76 (0.73–0.78)
Acute respiratory	663	16615	4.0 (3.7–4.3)	963	16316	5.9 (5.5–6.3)	0.68 (0.61–0.75)
Pulmonary edema	1513	16322	9.3 (8.8–9.8)	2208	15775	14.0 (13.4–14.6)	0.66 (0.62–0.71)
Dyspnea exertional	7569	9288	81.5 (79.7–83.3)	9453	8616	109.7 (107.5–111.9)	0.74 (0.72–0.76)
Pneumonitis	56	16811	0.3 (0.3–0.4)	11	16584	0.1 (0.0–0.1)	5.01 (2.63–9.57)
Hypoxia	3782	16413	23.0 (22.3–23.8)	5488	15944	34.4 (33.5–35.3)	0.67 (0.64–0.70)

Table S9. Incidence Rates of Cardiac and Vascular Adverse Events in Patients Comprised Primary Cohort

	**Dronedarone (N=12,210)**			**Amiodarone (N=12,210)**			**Dronedarone vs Amiodarone**
	N	Patient-time at risk (years)	Rate (Per 100 patient-years) (95% CI)	N	Patient-time at risk (years)	Rate (Per 100 patient-years) (95% CI)	IRR (95% CI)
Any Cardiac and Vascular AEs	1622	1690	96.0 (91.4–100.8)	1931	1614	119.6 (114.3–125.1)	0.80 (0.75–0.86)
Bradycardia	2017	11917	16.9 (16.2–17.7)	2428	11705	20.7 (19.9–21.6)	0.82 (0.77–0.87)
Dizziness	1575	11908	13.2 (12.6–13.9)	1822	11870	15.4 (14.7–16.1)	0.86 (0.81–0.92)
Tachycardia	1318	11669	11.3 (10.7–11.9)	1246	11992	10.4 (9.8–11.0)	1.09 (1.01–1.17)
Hypotension	1245	14237	8.7 (8.3–9.2)	1494	13422	11.1 (10.6–11.7)	0.79 (0.73–0.85)
Qt prolonged	172	16592	1.0 (0.9–1.2)	193	16251	1.2 (1.0–1.4)	0.87 (0.71–1.07)
Ventricular tachycardia	1088	13626	8.0 (7.5–8.5)	850	13326	6.4 (6.0–6.8)	1.25 (1.14–1.37)
Cardiac failure	1129	14944	7.6 (7.1–8.0)	1607	14150	11.4 (10.8–11.9)	0.67 (0.62–0.72)
Torsade de pointes	0	16791	--	0	16532	--	--
Cardiac arrest	163	16672	1.0 (0.8–1.1)	256	16228	1.6 (1.4–1.8)	0.62 (0.51–0.75)
Chest pain	1902	7505	25.3 (24.2–26.5)	2252	7208	31.2 (30.0–32.6)	0.81 (0.76–0.86)
Ventricular fibrillation	104	16537	0.6 (0.5–0.8)	135	16170	0.8 (0.7–1.0)	0.75 (0.58–0.97)
Peripheral edema	1937	12732	15.2 (14.5–15.9)	2423	11862	20.4 (19.6–21.3)	0.75 (0.70–0.79)
Congestive heart failure	875	15697	5.6 (5.2–6.0)	1286	15008	8.6 (8.1–9.1)	0.65 (0.60–0.71)
Arrhythmia	2466	8806	28.0 (26.9–29.1)	2553	9235	27.6 (26.6–28.7)	1.01 (0.96–1.07)
Cardiogenic shock	69	16724	0.4 (0.3–0.5)	108	16375	0.7 (0.5–0.8)	0.63 (0.46–0.85)
Cardiorespiratory arrest	180	16650	1.1 (0.9–1.3)	281	16178	1.7 (1.5–2.0)	0.62 (0.52–0.75)
Gastrointestinal hemorrhage	956	15173	6.3 (5.9–6.7)	889	14894	6.0 (5.6–6.4)	1.06 (0.96–1.16)

Table S10. Event Rate of Cardiac and Vascular Adverse Events in Patients Comprised Primary Cohort

	**Dronedarone (N=12,210)**			**Amiodarone (N=12,210)**			**Dronedarone vs Amiodarone**
	N	Patient-time at risk (years)	Rate (Per 100 patient-years) (95% CI)	N	Patient-time at risk (years)	Rate (Per 100 patient-years) (95% CI)	RR (95% CI)
Any Cardiac and Vascular AEs	10433	3263	319.7 (313.6–325.9)	15569	3451	451.2 (444.1–458.3)	0.71 (0.69–0.72)
Bradycardia	4924	13808	35.7 (34.7–36.7)	6233	13878	44.9 (43.8–46.0)	0.79 (0.76–0.82)
Dizziness	3812	13389	28.5 (27.6–29.4)	4160	13511	30.8 (29.9–31.7)	0.92 (0.88–0.96)
Tachycardia	2707	12745	21.2 (20.5–22.1)	2634	12961	20.3 (19.6–21.1)	1.04 (0.99–1.10)
Hypotension	3783	15238	24.8 (24.0–25.6)	5750	14567	39.5 (38.5–40.5)	0.63 (0.60–0.65)
Qt prolonged	265	16734	1.6 (1.4–1.8)	343	16468	2.1 (1.9–2.3)	0.76 (0.65–0.89)
Ventricular tachycardia	2819	14527	19.4 (18.7–20.1)	2367	14057	16.8 (16.2–17.5)	1.15 (1.09–1.21)
Cardiac failure	6627	15813	41.9 (40.9–42.9)	13270	15506	85.6 (84.1–87.1)	0.49 (0.47–0.50)
Torsade de pointes	0	16813	--	0	16584	--	--
Cardiac arrest	536	16745	3.2 (2.9–3.5)	813	16393	5.0 (4.6–5.3)	0.64 (0.58–0.72)
Chest pain	4237	9163	46.2 (44.9–47.7)	5199	9145	56.9 (55.3–58.4)	0.81 (0.78–0.84)
Ventricular fibrillation	275	16621	1.7 (1.5–1.9)	305	16345	1.9 (1.7–2.1)	0.89 (0.75–1.04)
Peripheral edema	5508	14503	38.0 (37.0–39.0)	7157	13945	51.3 (50.1–52.5)	0.74 (0.71–0.77)
Congestive heart failure	7195	16354	44.0 (43.0–45.0)	13680	16056	85.2 (83.8–86.6)	0.52 (0.50–0.53)
Arrhythmia	5633	11099	50.8 (49.4–52.1)	6153	11459	53.7 (52.4–55.1)	0.94 (0.91–0.98)
Cardiogenic shock	275	16782	1.6 (1.5–1.8)	385	16489	2.3 (2.1–2.6)	0.70 (0.60–0.82)
Cardiorespiratory arrest	592	16732	3.5 (3.3–3.8)	850	16363	5.2 (4.9–5.6)	0.68 (0.61–0.76)
Gastrointestinal hemorrhage	4616	16023	28.8 (28.0–29.7)	4311	15671	27.5 (26.7–28.3)	1.05 (1.00–1.09)

Table S11. Incidence Rates of General Adverse Events in Patients Comprised Primary Cohort

	**Dronedarone (N=12,210)**			**Amiodarone (N=12,210)**			**Dronedarone vs Amiodarone**
	N	Patient-time at risk (years)	Rate (Per 100 patient-years) (95% CI)	N	Patient-time at risk (years)	Rate (Per 100 patient-years) (95% CI)	IRR (95% CI)
Any general event	2865	7266	39.4 (38.0–40.9)	3370	6549	51.5 (49.7–53.2)	0.77 (0.73–0.81)
Asthenia	2145	10556	20.3 (19.5–21.2)	2569	10165	25.3 (24.3–26.3)	0.80 (0.76–0.85)
Fatigue	2279	10051	22.7 (21.8–23.6)	2653	9894	26.8 (25.8–27.9)	0.85 (0.80–0.89)
Weight decreased	586	15766	3.7 (3.4–4.0)	692	15463	4.5 (4.2–4.8)	0.83 (0.74–0.93)
Weight increased	145	16504	0.9 (0.7–1.0)	180	16243	1.1 (1.0–1.3)	0.79 (0.64–0.99)
Malaise	1831	11268	16.3 (15.5–17.0)	2210	10739	20.6 (19.7–21.5)	0.79 (0.74–0.84)
Edema	1958	12707	15.4 (14.7–16.1)	2442	11815	20.7 (19.9–21.5)	0.75 (0.70–0.79)

Table S12. Event Rates of General Adverse Events in Patients Comprised Primary Cohort

	**Dronedarone (N=12,210)**			**Amiodarone (N=12,210)**			**Dronedarone vs Amiodarone**
	N	Patient-time at risk (years)	Rate (Per 100 patient-years) (95% CI)	N	Patient-time at risk (years)	Rate (Per 100 patient-years) (95% CI)	RR (95% CI)
Any general event	16710	9843	169.8 (167.2–172.4)	20423	9460	215.9 (212.9–218.9)	0.78 (0.77–0.80)
Asthenia	6389	12394	51.6 (50.3–52.8)	8411	12283	68.5 (67.0–70.0)	0.75 (0.73–0.78)
Fatigue	5371	12059	44.5 (43.4–45.8)	6827	12158	56.2 (54.8–57.5)	0.79 (0.76–0.82)
Weight decreased	1567	16278	9.6 (9.2–10.1)	1845	16032	11.5 (11.0–12.1)	0.84 (0.78–0.89)
Weight increased	193	16638	1.2 (1.0–1.3)	243	16428	1.5 (1.3–1.7)	0.78 (0.65–0.95)
Malaise	5539	12857	43.1 (42.0–44.2)	7274	12616	57.7 (56.3–59.0)	0.75 (0.72–0.77)
Edema	5635	14494	38.9 (37.9–39.9)	7347	13908	52.8 (51.6–54.1)	0.73 (0.71–0.76)

Table S13. Incidence Rates of Endocrine and Metabolic Adverse Events in Patients Comprised Primary Cohort

	**Dronedarone (N=12,210)**			**Amiodarone (N=12,210)**			**Dronedarone vs Amiodarone**
	N	Patient-time at risk (years)	Rate (Per 100 patient-years) (95% CI)	N	Patient-time at risk (years)	Rate (Per 100 patient-years) (95% CI)	IRR (95% CI)
Any endocrine and metabolic event	1115	11189	10.0 (9.4–10.6)	2054	10624	19.3 (18.5–20.2)	0.52 (0.48–0.55)
Hyperthyroidism	165	16240	1.0 (0.9–1.2)	305	16038	1.9 (1.7–2.1)	0.53 (0.44–0.65)
Hypothyroidism	827	12108	6.8 (6.4–7.3)	1747	11474	15.2 (14.5–16.0)	0.45 (0.41–0.49)
Thyroid disorder	534	15356	3.5 (3.2–3.8)	596	15266	3.9 (3.6–4.2)	0.89 (0.79–1.00)
Decreased appetite	233	16485	1.4 (1.2–1.6)	339	16129	2.1 (1.9–2.3)	0.67 (0.57–0.79)

Table S14. Event Rates of Endocrine and Metabolic Adverse Events in Patients Comprised Primary Cohort

	**Dronedarone (N=12,210)**			**Amiodarone (N=12,210)**			**Dronedarone vs Amiodarone**
	N	Patient-time at risk (years)	Rate (Per 100 patient-years) (95% CI)	N	Patient-time at risk (years)	Rate (Per 100 patient-years) (95% CI)	RR (95% CI)
Any endocrine and metabolic event	3537	12186	29.0 (28.1–30.0)	9570	12419	77.1 (75.5–78.6)	0.38 (0.36–0.39)
Hyperthyroidism	437	16402	2.7 (2.4–2.9)	840	16295	5.2 (4.8–5.5)	0.52 (0.46–0.58)
Hypothyroidism	2553	12890	19.8 (19.0–20.6)	8073	13067	61.8 (60.4–63.2)	0.32 (0.31–0.33)
Thyroid disorder	1288	15835	8.1 (7.7–8.6)	1424	15792	9.0 (8.6–9.5)	0.90 (0.84–0.97)
Decreased appetite	572	16649	3.4 (3.2–3.7)	738	16390	4.5 (4.2–4.8)	0.76 (0.68–0.85)

Table S15. Incidence Rates of Gastrointestinal and Hepatobiliary Adverse Events in Patients Comprised Primary Cohort

	Dronedarone (N=12,210)			Amiodarone (N=12,210)			Dronedarone vs Amiodarone
	N	Patient-time at risk (years)	Rate (Per 100 patient-years) (95% CI)	N	Patient-time at risk (years)	Rate (Per 100 patient-years) (95% CI)	IRR (95% CI)
Any gastrointestinal and hepatobiliary event	2422	10258	23.6 (22.7–24.6)	2536	9677	26.2 (25.2–27.3)	0.90 (0.85–0.95)
Nausea	1211	14224	8.5 (8.0–9.0)	1556	13428	11.6 (11.0–12.2)	0.73 (0.68–0.79)
Vomiting	816	15211	5.4 (5.0–5.8)	1102	14403	7.7 (7.2–8.1)	0.70 (0.64–0.77)
Diarrhea	1006	14739	6.8 (6.4–7.3)	927	14524	6.4 (6.0–6.8)	1.07 (0.98–1.17)
Abdominal pain	1962	12013	16.3 (15.6–17.1)	2062	11462	18.0 (17.2–18.8)	0.91 (0.85–0.97)
Liver injury	11	16775	0.1 (0.0–0.1)	4	16517	0.0 (0.0–0.1)	2.71 (0.86–8.51)
Hepatic failure	100	16644	0.6 (0.5–0.7)	159	16362	1.0 (0.8–1.1)	0.62 (0.48–0.79)

Table S16. Event Rates of Gastrointestinal and Hepatobiliary Adverse Events in Patients Comprised Primary Cohort

	**Dronedarone (N=12,210)**			**Amiodarone (N=12,210)**			**Dronedarone vs Amiodarone**
	N	Patient-time at risk (years)	Rate (Per 100 patient-years) (95% CI)	N	Patient-time at risk (years)	Rate (Per 100 patient-years) (95% CI)	RR (95% CI)
Any gastrointestinal and hepatobiliary event	9402	12427	75.7 (74.1–77.2)	10894	11736	92.8 (91.1–94.6)	0.81 (0.79–0.84)
Nausea	3027	15309	19.8 (19.1–20.5)	4270	14701	29.1 (28.2–29.9)	0.68 (0.65–0.71)
Vomiting	1921	15917	12.1 (11.5–12.6)	3000	15302	19.6 (18.9–20.3)	0.61 (0.58–0.65)
Diarrhea	2637	15670	16.8 (16.2–17.5)	2812	15256	18.4 (17.8–19.1)	0.91 (0.86–0.96)
Abdominal pain	4873	13744	35.5 (34.5–36.5)	5232	13103	39.9 (38.9–41.0)	0.89 (0.85–0.92)
Liver injury	21	16803	0.1 (0.1–0.2)	4	16572	0.0 (0.0–0.1)	5.17 (1.77–15.06)
Hepatic failure	416	16720	2.5 (2.3–2.7)	722	16491	4.4 (4.1–4.7)	0.57 (0.50–0.64)

Table S17. Incidence Rates of Nervous Psychiatric Adverse Events in Patients Comprised Primary Cohort

	**Dronedarone (N=12,210)**			**Amiodarone (N=12,210)**			**Dronedarone vs Amiodarone**
	N	Patient-time at risk (years)	Rate (Per 100 patient-years) (95% CI)	N	Patient-time at risk (years)	Rate (Per 100 patient-years) (95% CI)	IRR (95% CI)
Any neurological event	1943	10499	18.5 (17.7–19.4)	2296	9898	23.2 (22.3–24.2)	0.80 (0.75–0.85)
Syncope	1055	13628	7.7 (7.3–8.2)	1226	13389	9.2 (8.7–9.7)	0.85 (0.78–0.92)
Confusional state	576	15900	3.6 (3.3–3.9)	772	15167	5.1 (4.7–5.5)	0.71 (0.64–0.79)
Insomnia	747	15087	5.0 (4.6–5.3)	927	14759	6.3 (5.9–6.7)	0.79 (0.72–0.87)
Depression	997	14281	7.0 (6.6–7.4)	1104	13735	8.0 (7.6–8.5)	0.87 (0.80–0.95)

Table S18. Event Rates of Nervous Psychiatric Adverse Events in Patients Comprised Primary Cohort

	**Dronedarone (N=12,210)**			**Amiodarone (N=12,210)**			**Dronedarone vs Amiodarone**
	N	Patient-time at risk (years)	Rate (Per 100 patient-years) (95% CI)	N	Patient-time at risk (years)	Rate (Per 100 patient-years) (95% CI)	RR (95% CI)
Any neurological event	7400	12123	61.0 (59.7–62.5)	9739	11760	82.8 (81.2–84.5)	0.74 (0.71–0.76)
Syncope	3279	14467	22.7 (21.9–23.5)	4436	14422	30.8 (29.9–31.7)	0.74 (0.70–0.77)
Confusional state	1701	16274	10.5 (10.0–11.0)	2571	15724	16.4 (15.7–17.0)	0.64 (0.60–0.68)
Insomnia	1612	15797	10.2 (9.7–10.7)	2195	15552	14.1 (13.5–14.7)	0.72 (0.68–0.77)
Depression	4006	15079	26.6 (25.8–27.4)	5209	14615	35.6 (34.7–36.6)	0.74 (0.71–0.78)

Table S19. Incidence Rates of Musculoskeletal Adverse Events in Patients Comprised Primary Cohort

	**Dronedarone (N=12,210)**			**Amiodarone (N=12,210)**			**Dronedarone vs Amiodarone**
	**N**	**Patient-time at risk (years)**	**Rate (Per 100 patient-years) (95% CI)**	**N**	**Patient-time at risk (years)**	**Rate (Per 100 patient-years) (95% CI)**	**IRR (95% CI)**
Any musculoskeletal event	1513	13415	11.3 (10.7–11.9)	2034	12503	16.3 (15.6–17.0)	0.69 (0.65–0.74)
Gait disturbance	1174	14414	8.1 (7.7–8.6)	1743	13413	13.0 (12.4–13.6)	0.63 (0.58–0.68)
Rhabdomyolysis	105	16620	0.6 (0.5–0.8)	164	16279	1.0 (0.9–1.2)	0.63 (0.49–0.80)
Myalgia	455	15630	2.9 (2.7–3.2)	474	15417	3.1 (2.8–3.4)	0.95 (0.83–1.08)

Table S20. Event Rate of Musculoskeletal Adverse Events in Patients Comprised Primary Cohort

	**Dronedarone (N=12,210)**			**Amiodarone (N=12,210)**			**Dronedarone vs Amiodarone**
	**N**	**Patient-time at risk (years)**	**Rate (Per 100 patient-years) (95% CI)**	**N**	**Patient-time at risk (years)**	**Rate (Per 100 patient-years) (95% CI)**	**RR (95% CI)**
Any musculoskeletal event	10228	14718	69.5 (68.2–70.9)	16404	14233	115.3 (113.5–117.0)	0.60 (0.59–0.62)
Gait disturbance	8794	15424	57.0 (55.8–58.2)	15480	14884	104.0 (102.4–105.7)	0.55 (0.53–0.56)
Rhabdomyolysis	570	16707	3.4 (3.1–3.7)	942	16432	5.7 (5.4–6.1)	0.59 (0.54–0.66)
Myalgia	1401	16095	8.7 (8.3–9.2)	1039	15880	6.5 (6.2–7.0)	1.33 (1.23–1.44)

Table S21. Incidence Rates of Other Adverse Events in Patients Comprised Primary Cohort

	Dronedarone (N=12,210)			Amiodarone (N=12,210)			Dronedarone vs Amiodarone
	N	Patient-time at risk (years)	Rate (Per 100 patient-years) (95% CI)	N	Patient-time at risk (years)	Rate (Per 100 patient-years) (95% CI)	IRR (95% CI)
Renal and Urinary disorders							
Any renal event	1453	12846	11.3 (10.7–11.9)	2001	11760	17.0 (16.3–17.8)	0.66 (0.62–0.71)
Acute kidney	1231	14546	8.5 (8.0–9.0)	1639	13443	12.2 (11.6–12.8)	0.69 (0.64–0.75)
Renal failure	845	13981	6.0 (5.6–6.5)	1286	13473	9.5 (9.0–10.1)	0.63 (0.58–0.69)
Blood Disorders							
Any blood related event	1337	13927	9.6 (9.1–10.1)	1666	13102	12.7 (12.1–13.3)	0.76 (0.70–0.81)
Anemia	1136	14444	7.9 (7.4–8.3)	1404	13821	10.2 (9.6–10.7)	0.77 (0.72–0.84)
Thrombocytopenia	406	16016	2.5 (2.3–2.8)	510	15532	3.3 (3.0–3.6)	0.77 (0.68–0.88)
Any ocular event			0.0 (0.0–0.0)				
Eye disorders	790	14951	5.3 (4.9–5.7)	868	14714	5.9 (5.5–6.3)	0.90 (0.81–0.99)
Blindness	84	16620	0.5 (0.4–0.6)	115	16328	0.7 (0.6–0.9)	0.72 (0.54–0.95)
Visual impairment	202	16281	1.2 (1.1–1.4)	287	15924	1.8 (1.6–2.0)	0.69 (0.58–0.82)
Vision blurred	591	15472	3.8 (3.5–4.1)	597	15375	3.9 (3.6–4.2)	0.98 (0.88–1.10)
Skin and subcutaneous tissue disorders							
Rash	619	15637	4.0 (3.7–4.3)	598	15487	3.9 (3.6–4.2)	1.03 (0.92–1.15)
Pruritus	234	16366	1.4 (1.3–1.6)	189	16193	1.2 (1.0–1.4)	1.23 (1.01–1.48)
Overall							
Any safety event of any cate	463	295	156.9 (143.0–171.9)	560	288	194.7 (178.9–211.5)	0.81 (0.71–0.91)

Table S22. Event Rates of Other Adverse Events in Patients Comprised Primary Cohort

	**Dronedarone (N=12,210)**			**Amiodarone (N=12,210)**			**Dronedarone vs Amiodarone**
	N	Patient-time at risk (years)	Rate (Per 100 patient-years) (95% CI)	N	Patient-time at risk (years)	Rate (Per 100 patient-years) (95% CI)	RR (95% CI)
Renal and Urinary disorders							
Any renal event	9901	14124	70.1 (68.7–71.5)	16631	13444	123.7 (121.8–125.6)	0.57 (0.55–0.58)
Acute kidney	7987	15493	51.6 (50.4–52.7)	10989	14655	75.0 (73.6–76.4)	0.69 (0.67–0.71)
Renal failure	4211	14866	28.3 (27.5–29.2)	8487	14685	57.8 (56.6–59.0)	0.49 (0.47–0.51)
Blood Disorders							
Any blood related event	7261	15110	48.1 (47.0–49.2)	8990	14484	62.1 (60.8–63.4)	0.77 (0.75–0.80)
Anemia	6356	15467	41.1 (40.1–42.1)	7788	15008	51.9 (50.8–53.1)	0.79 (0.76–0.82)
Thrombocytopenia	1247	16319	7.6 (7.2–8.1)	2088	15936	13.1 (12.6–13.7)	0.58 (0.54–0.62)
Any ocular event							
Eye disorders	1598	15700	10.2 (9.7–10.7)	2610	15487	16.9 (16.2–17.5)	0.60 (0.57–0.64)
Blindness	230	16705	1.4 (1.2–1.6)	574	16461	3.5 (3.2–3.8)	0.39 (0.34–0.46)
Visual impairment	424	16471	2.6 (2.3–2.8)	1187	16204	7.3 (6.9–7.8)	0.35 (0.31–0.39)
Vision blurred	1024	16081	6.4 (6.0–6.8)	1058	15929	6.6 (6.3–7.1)	0.96 (0.88–1.04)
Skin and subcutaneous tissue disorders							
Rash	984	16241	6.1 (5.7–6.5)	986	16027	6.2 (5.8–6.6)	0.98 (0.90–1.07)
Pruritus	436	16589	2.6 (2.4–2.9)	368	16391	2.3 (2.0–2.5)	1.17 (1.02–1.34)
Overall							
Any safety event of any cate	4778	738	647.6 (629.3–666.2)	9774	833	1,173.3 (1,150.1–1,196.8)	0.55 (0.53–0.91)

Table S23. Incidence Rates of Respiratory Adverse Events With Intent-to-Treat Analysis

	**Dronedarone (N=12,210)**			**Amiodarone (N=12,210)**			**Dronedarone vs Amiodarone**
	**N**	**Patient-time at risk (years)**	**Rate (Per 100 patient-years) (95% CI)**	**N**	**Patient-time at risk (years)**	**Rate (Per 100 patient-years) (95% CI)**	**IRR (95% CI)**
Any Respiratory event	3617	9821	36.8 (35.6–38.1)	3841	7537	51.0 (49.4–52.6)	0.72 (0.69–0.76)
Dyspnea	4376	21145	20.7 (20.1–21.3)	5057	18836	26.9 (26.1–27.6)	0.77 (0.74–0.80)
Interstitial lung	781	52026	1.5 (1.4–1.6)	960	52926	1.8 (1.7–1.9)	0.83 (0.75–0.91)
Cough	4134	32550	12.7 (12.3–13.1)	4575	32719	14.0 (13.6–14.4)	0.91 (0.87–0.95)
Pulmonary fibrosis	575	53931	1.1 (1.0–1.2)	545	55898	1.0 (0.9–1.1)	1.09 (0.97–1.23)
Pulmonary toxicity	475	52654	0.9 (0.8–1.0)	664	53598	1.2 (1.2–1.3)	0.73 (0.65–0.82)
Respiratory failure	2705	47993	5.6 (5.4–5.9)	3360	46544	7.2 (7.0–7.5)	0.78 (0.74–0.82)
Lung disorder	3238	28121	11.5 (11.1–11.9)	4007	28253	14.2 (13.8–14.6)	0.81 (0.78–0.85)
Wheezing	996	51444	1.9 (1.8–2.1)	1125	52662	2.1 (2.0–2.3)	0.91 (0.83–0.99)
Pleural effusion	3080	44780	6.9 (6.6–7.1)	3780	40991	9.2 (8.9–9.5)	0.75 (0.71–0.78)
Acute respiratory	603	53859	1.1 (1.0–1.2)	876	54891	1.6 (1.5–1.7)	0.70 (0.63–0.78)
Pulmonary edema	1792	49638	3.6 (3.4–3.8)	2269	49306	4.6 (4.4–4.8)	0.78 (0.74–0.83)
Dyspnea exertional	4211	18135	23.2 (22.5–23.9)	4814	15673	30.7 (29.9–31.6)	0.76 (0.73–0.79)
Pneumonitis	22	55372	0.0 (0.0–0.1)	34	57168	0.1 (0.0–0.1)	0.67 (0.39–1.14)
Hypoxia	2225	50515	4.4 (4.2–4.6)	2868	50737	5.7 (5.5–5.9)	0.78 (0.74–0.82)

Table S24. Event Rates of Respiratory Adverse Events With Intent-to-Treat Analysis

	**Dronedarone (N=12,210)**			**Amiodarone (N=12,210)**			**Dronedarone vs Amiodarone**
	**N**	**Patient-time at risk (years)**	**Rate (Per 100 patient-years) (95% CI)**	**N**	**Patient-time at risk (years)**	**Rate (Per 100 patient-years) (95% CI)**	**RR (95% CI)**
Any Respiratory event	68073	25356	268.5 (266.5–270.5)	84851	23764	357.1 (354.7–359.5)	0.75 (0.74–0.76)
Dyspnea	22527	40004	56.3 (55.6–57.1)	27548	39037	70.6 (69.7–71.4)	0.80 (0.78–0.81)
Interstitial lung	3174	62845	5.1 (4.9–5.2)	4373	64370	6.8 (6.6–7.0)	0.74 (0.71–0.78)
Cough	10896	52714	20.7 (20.3–21.1)	12400	53888	23.0 (22.6–23.4)	0.90 (0.87–0.92)
Pulmonary fibrosis	4039	63862	6.3 (6.1–6.5)	2425	65739	3.7 (3.5–3.8)	1.71 (1.63–1.80)
Pulmonary toxicity	1619	63042	2.6 (2.4–2.7)	2804	64633	4.3 (4.2–4.5)	0.59 (0.56–0.63)
Respiratory failure	33196	61570	53.9 (53.3–54.5)	39766	60664	65.6 (64.9–66.2)	0.82 (0.81–0.83)
Lung disorder	32738	45070	72.6 (71.9–73.4)	44786	47865	93.6 (92.7–94.4)	0.77 (0.76–0.79)
Wheezing	1612	62949	2.6 (2.4–2.7)	1894	64160	3.0 (2.8–3.1)	0.87 (0.81–0.93)
Pleural effusion	17820	60348	29.5 (29.1–30.0)	21662	58100	37.3 (36.8–37.8)	0.79 (0.77–0.81)
Acute respiratory	1707	63600	2.7 (2.6–2.8)	2341	65031	3.6 (3.5–3.8)	0.74 (0.70–0.79)
Pulmonary edema	5092	62469	8.2 (7.9–8.4)	6726	62762	10.7 (10.5–11.0)	0.76 (0.73–0.79)
Dyspnea exertional	23245	36372	63.9 (63.1–64.7)	27997	35299	79.3 (78.4–80.3)	0.80 (0.79–0.82)
Pneumonitis	160	64247	0.3 (0.2–0.3)	184	65983	0.3 (0.2–0.3)	0.89 (0.72–1.10)
Hypoxia	27097	62969	43.0 (42.5–43.6)	32177	63983	50.3 (49.7–50.8)	0.85 (0.84–0.87)

Table S25. Event Rates of Cardiac and Vascular Adverse Events With Intent-to-Treat Analysis

	Dronedarone (N=12,210)			Amiodarone (N=12,210)			Dronedarone vs Amiodarone
	N	Patient-time at risk (years)	Rate (Per 100 patient-years) (95% CI)	N	Patient-time at risk (years)	Rate (Per 100 patient-years) (95% CI)	IRR (95% CI)
Any CV event	2296	3293	69.7 (66.9–72.6)	2709	3254	83.3 (80.2–86.5)	0.84 (0.79–0.89)
Bradycardia	3479	34305	10.1 (9.8–10.5)	4078	35008	11.7 (11.3–12.0)	0.87 (0.83–0.91)
Dizziness	3310	33729	9.8 (9.5–10.2)	3765	34879	10.8 (10.5–11.1)	0.91 (0.87–0.95)
Tachycardia	2943	35252	8.4 (8.1–8.7)	2983	38329	7.8 (7.5–8.1)	1.07 (1.02–1.13)
Hypotension	2918	43021	6.8 (6.5–7.0)	3534	41831	8.5 (8.2–8.7)	0.80 (0.76–0.84)
Qt prolonged	406	54202	0.8 (0.7–0.8)	456	55877	0.8 (0.7–0.9)	0.92 (0.80–1.05)
Ventricular tachycardia	2185	42046	5.2 (5.0–5.4)	1988	43936	4.5 (4.3–4.7)	1.15 (1.08–1.22)
Cardiac failure	3424	44301	7.7 (7.5–8.0)	4381	43840	10.0 (9.7–10.3)	0.77 (0.74–0.81)
Torsade de pointes	0	55420	--	0	57234	--	--
Cardiac arrest	616	54687	1.1 (1.0–1.2)	970	55842	1.7 (1.6–1.9)	0.65 (0.59–0.72)
Chest pain	3630	19411	18.7 (18.1–19.3)	3995	19065	21.0 (20.3–21.6)	0.89 (0.85–0.93)
Ventricular fibrillation	272	54327	0.5 (0.4–0.6)	330	55567	0.6 (0.5–0.7)	0.84 (0.72–0.99)
Peripheral edema	4034	35908	11.2 (10.9–11.6)	4704	34444	13.7 (13.3–14.1)	0.82 (0.79–0.86)
Congestive heart failure	2849	47861	6.0 (5.7–6.2)	3733	47797	7.8 (7.6–8.1)	0.76 (0.73–0.80)
Arrhythmia	3974	23461	16.9 (16.4–17.5)	4344	25871	16.8 (16.3–17.3)	1.01 (0.97–1.05)
Cardiogenic shock	245	54952	0.5 (0.4–0.5)	351	56576	0.6 (0.6–0.7)	0.72 (0.61–0.85)
Cardiorespiratory arrest	641	54581	1.2 (1.1–1.3)	1015	55676	1.8 (1.7–1.9)	0.64 (0.58–0.71)
Gastrointestinal hemorrhage	2108	46799	4.5 (4.3–4.7)	2275	47861	4.8 (4.6–5.0)	0.95 (0.89–1.01)

Table S26. Event Rates of Cardiac and Vascular Adverse Events With Intent-to-Treat Analysis

	**Dronedarone (N=12,210)**			**Amiodarone (N=12,210)**			**Dronedarone vs Amiodarone**
	**N**	**Patient-time at risk (years)**	**Rate (Per 100 patient-years) (95% CI)**	**N**	**Patient-time at risk (years)**	**Rate (Per 100 patient-years) (95% CI)**	**RR (95% CI)**
Any CV event	44627	12961	344.3 (341.1–347.5)	59915	14278	419.6 (416.3–423.0)	0.81 (0.80–0.82)
Bradycardia	11666	52614	22.2 (21.8–22.6)	14169	54949	25.8 (25.4–26.2)	0.86 (0.84–0.88)
Dizziness	10985	51043	21.5 (21.1–21.9)	11804	53913	21.9 (21.5–22.3)	0.98 (0.96–1.01)
Tachycardia	8637	49597	17.4 (17.1–17.8)	8044	52756	15.3 (14.9–15.6)	1.14 (1.10–1.17)
Hypotension	12769	58595	21.8 (21.4–22.2)	16810	58247	28.9 (28.4–29.3)	0.75 (0.74–0.77)
Qt prolonged	770	63965	1.2 (1.1–1.3)	952	65603	1.5 (1.4–1.6)	0.83 (0.75–0.91)
Ventricular tachycardia	8776	55754	15.7 (15.4–16.1)	8492	56948	14.9 (14.6–15.2)	1.05 (1.02–1.08)
Cardiac failure	54582	60969	89.5 (88.8–90.3)	75195	62486	120.3 (119.5–121.2)	0.74 (0.73–0.75)
Torsade de pointes	0	64252	--	0	65984	--	--
Cardiac arrest	1913	63992	3.0 (2.9–3.1)	2711	65362	4.2 (4.0–4.3)	0.72 (0.68–0.76)
Chest pain	11986	35511	33.8 (33.2–34.4)	13447	35857	37.5 (36.9–38.1)	0.90 (0.88–0.92)
Ventricular fibrillation	1016	63580	1.6 (1.5–1.7)	991	65046	1.5 (1.4–1.6)	1.05 (0.96–1.14)
Peripheral edema	18367	55878	32.9 (32.4–33.4)	22975	56104	41.0 (40.4–41.5)	0.80 (0.79–0.82)
Congestive heart failure	54819	62816	87.3 (86.5–88.0)	75017	64378	116.5 (115.7–117.4)	0.75 (0.74–0.76)
Arrhythmia	12402	42375	29.3 (28.8–29.8)	14695	45762	32.1 (31.6–32.6)	0.91 (0.89–0.93)
Cardiogenic shock	1036	64116	1.6 (1.5–1.7)	1410	65728	2.2 (2.0–2.3)	0.75 (0.69–0.81)
Cardiorespiratory arrest	1989	63939	3.1 (3.0–3.3)	2806	65266	4.3 (4.1–4.5)	0.72 (0.68–0.76)
Gastrointestinal hemorrhage	11849	61420	19.3 (19.0–19.6)	13142	62467	21.0 (20.7–21.4)	0.91 (0.89–0.94)

Table S27. Incidence Rates of General Adverse Events With Intent-to-Treat Analysis

	**Dronedarone (N=12,210)**			**Amiodarone (N=12,210)**			**Dronedarone vs Amiodarone**
	**N**	**Patient-time at risk (years)**	**Rate (Per 100 patient-years) (95% CI)**	**N**	**Patient-time at risk (years)**	**Rate (Per 100 patient-years) (95% CI)**	**IRR (95% CI)**
Any general event	5008	17237	29.1 (28.3–29.9)	5667	15681	36.1 (35.2–37.1)	0.80 (0.77–0.84)
Asthenia	4032	28018	14.4 (14.0–14.8)	4905	27693	17.7 (17.2–18.2)	0.81 (0.78–0.85)
Fatigue	4161	26425	15.8 (15.3–16.2)	4890	26932	18.2 (17.7–18.7)	0.87 (0.83–0.90)
Weight decreased	1529	49950	3.1 (2.9–3.2)	1768	50888	3.5 (3.3–3.6)	0.88 (0.82–0.94)
Weight increased	378	53831	0.7 (0.6–0.8)	446	55551	0.8 (0.7–0.9)	0.87 (0.76–1.00)
Malaise	3240	30415	10.7 (10.3–11.0)	3906	29898	13.1 (12.7–13.5)	0.82 (0.78–0.85)
Edema	4074	35821	11.4 (11.0–11.7)	4762	34310	13.9 (13.5–14.3)	0.82 (0.79–0.85)

Table S28. Event Rates of General Adverse Events With Intent-to-Treat Analysis

	**Dronedarone (N=12,210)**			**Amiodarone (N=12,210)**			**Dronedarone vs Amiodarone**
	**N**	**Patient-time at risk (years)**	**Rate (Per 100 patient-years) (95% CI)**	**N**	**Patient-time at risk (years)**	**Rate (Per 100 patient-years) (95% CI)**	**RR (95% CI)**
Any general event	52358	38722	135.2 (134.1–136.4)	63576	38485	165.2 (163.9–166.5)	0.82 (0.81–0.83)
Asthenia	18979	47833	39.7 (39.1–40.3)	26433	48710	54.3 (53.6–54.9)	0.73 (0.72–0.74)
Fatigue	14582	46728	31.2 (30.7–31.7)	17992	48322	37.2 (36.7–37.8)	0.84 (0.82–0.85)
Weight decreased	5041	62381	8.1 (7.9–8.3)	5646	63869	8.8 (8.6–9.1)	0.91 (0.88–0.95)
Weight increased	728	63648	1.1 (1.1–1.2)	744	65437	1.1 (1.1–1.2)	1.00 (0.91–1.11)
Malaise	14606	49227	29.7 (29.2–30.2)	18489	49611	37.3 (36.7–37.8)	0.79 (0.78–0.81)
Edema	19079	55852	34.2 (33.7–34.7)	24249	56009	43.3 (42.8–43.8)	0.79 (0.77–0.80)

Table S29. Incidence Rates of Endocrine and Metabolic Adverse Events With Intent-to-Treat Analysis

	**Dronedarone (N=12,210)**			**Amiodarone (N=12,210)**			**Dronedarone vs Amiodarone**
	**N**	**Patient-time at risk (years)**	**Rate (Per 100 patient-years) (95% CI)**	**N**	**Patient-time at risk (years)**	**Rate (Per 100 patient-years) (95% CI)**	**IRR (95% CI)**
Any endocrine and metabolic event	2445	32604	7.5 (7.2–7.8)	3844	31028	12.4 (12.0–12.8)	0.61 (0.58–0.64)
Hyperthyroidism	501	52542	1.0 (0.9–1.0)	756	53927	1.4 (1.3–1.5)	0.68 (0.61–0.76)
Hypothyroidism	1838	36353	5.1 (4.8–5.3)	3091	35101	8.8 (8.5–9.1)	0.57 (0.54–0.61)
Thyroid disorder	1323	48286	2.7 (2.6–2.9)	1594	50144	3.2 (3.0–3.3)	0.86 (0.80–0.93)
Decreased appetite	689	53551	1.3 (1.2–1.4)	899	54798	1.6 (1.5–1.8)	0.78 (0.71–0.87)

Table S30. Event Rates of Endocrine and Metabolic Adverse Events With Intent-to-Treat Analysis

	Dronedarone (N=12,210)			Amiodarone (N=12,210)			Dronedarone vs Amiodarone
	**N**	**Patient-time at risk (years)**	**Rate (Per 100 patient-years) (95% CI)**	**N**	**Patient-time at risk (years)**	**Rate (Per 100 patient-years) (95% CI)**	**RR (95% CI)**
Any endocrine and metabolic event	19968	46568	42.9 (42.3–43.5)	39921	49633	80.4 (79.6–81.2)	0.53 (0.52–0.54)
Hyperthyroidism	2331	62581	3.7 (3.6–3.9)	4036	64711	6.2 (6.1–6.4)	0.60 (0.57–0.63)
Hypothyroidism	15808	49161	32.2 (31.7–32.7)	34456	52099	66.1 (65.4–66.8)	0.49 (0.48–0.49)
Thyroid disorder	5088	60583	8.4 (8.2–8.6)	5358	62935	8.5 (8.3–8.7)	0.98 (0.95–1.02)
Decreased appetite	1658	63700	2.6 (2.5–2.7)	2037	65318	3.1 (3.0–3.3)	0.83 (0.78–0.89)

Table S31. Incidence Rates of Gastrointestinal and Hepatobiliary Adverse Events With Intent-to-Treat Analysis

	**Dronedarone (N=12,210)**			**Amiodarone (N=12,210)**			**Dronedarone vs Amiodarone**
	**N**	**Patient-time at risk (years)**	**Rate (Per 100 patient-years) (95% CI)**	**N**	**Patient-time at risk (years)**	**Rate (Per 100 patient-years) (95% CI)**	**IRR (95% CI)**
Any gastrointestinal and hepatobiliary event	4908	26227	18.7 (18.2–19.2)	5138	25716	20.0 (19.4–20.5)	0.94 (0.90–0.97)
Nausea	2818	42431	6.6 (6.4–6.9)	3266	41529	7.9 (7.6–8.1)	0.84 (0.80–0.89)
Vomiting	1976	47082	4.2 (4.0–4.4)	2468	46125	5.4 (5.1–5.6)	0.78 (0.74–0.83)
Diarrhea	2423	44743	5.4 (5.2–5.6)	2405	46401	5.2 (5.0–5.4)	1.04 (0.99–1.11)
Abdominal pain	4244	32627	13.0 (12.6–13.4)	4477	32204	13.9 (13.5–14.3)	0.94 (0.90–0.98)
Liver injury	11	55364	0.0 (0.0–0.0)	8	57114	0.0 (0.0–0.0)	1.42 (0.57–3.53)
Hepatic failure	382	54497	0.7 (0.6–0.8)	509	56153	0.9 (0.8–1.0)	0.77 (0.68–0.88)

Table S32. Event Rates of Gastrointestinal and Hepatobiliary Adverse Events With Intent-to-Treat Analysis

	**Dronedarone (N=12,210)**			**Amiodarone (N=12,210)**			**Dronedarone vs Amiodarone**
	**N**	**Patient-time at risk (years)**	**Rate (Per 100 patient-years) (95% CI)**	**N**	**Patient-time at risk (years)**	**Rate (Per 100 patient-years) (95% CI)**	**RR (95% CI)**
Any gastrointestinal and hepatobiliary event	30188	47617	63.4 (62.7–64.1)	34932	47110	74.2 (73.4–74.9)	0.85 (0.84–0.87)
Nausea	9360	58519	16.0 (15.7–16.3)	11088	58613	18.9 (18.6–19.3)	0.84 (0.82–0.87)
Vomiting	6049	60845	9.9 (9.7–10.2)	7410	61077	12.1 (11.9–12.4)	0.82 (0.79–0.85)
Diarrhea	7773	60020	13.0 (12.7–13.2)	8220	60943	13.5 (13.2–13.8)	0.96 (0.93–0.99)
Abdominal pain	15419	52438	29.4 (28.9–29.9)	16281	52212	31.2 (30.7–31.7)	0.94 (0.92–0.96)
Liver injury	21	64238	0.0 (0.0–0.1)	8	65902	0.0 (0.0–0.0)	2.69 (1.19–6.07)
Hepatic failure	2834	63930	4.4 (4.3–4.6)	4189	65671	6.4 (6.2–6.6)	0.69 (0.66–0.73)

Table S33. Incidence Rates of Neurological Adverse Events With Intent-to-Treat Analysis

	Dronedarone (N=12,210)			Amiodarone (N=12,210)			Dronedarone vs Amiodarone
	**N**	**Patient-time at risk (years)**	**Rate (Per 100 patient-years) (95% CI)**	**N**	**Patient-time at risk (years)**	**Rate (Per 100 patient-years) (95% CI)**	**IRR (95% CI)**
Any neurological event	4070	28588	14.2 (13.8–14.7)	4753	28249	16.8 (16.4–17.3)	0.85 (0.81–0.88)
Syncope	2389	40938	5.8 (5.6–6.1)	2780	42569	6.5 (6.3–6.8)	0.89 (0.85–0.94)
Confusional state	1653	50103	3.3 (3.1–3.5)	2066	50185	4.1 (3.9–4.3)	0.80 (0.75–0.85)
Insomnia	1822	46786	3.9 (3.7–4.1)	2194	47591	4.6 (4.4–4.8)	0.84 (0.79–0.90)
Depression	2410	43405	5.6 (5.3–5.8)	2682	44262	6.1 (5.8–6.3)	0.92 (0.87–0.97)

Table S34. Event Rates of Neurological Adverse Events With Intent-to-Treat Analysis

	Dronedarone (N=12,210)			Amiodarone (N=12,210)			Dronedarone vs Amiodarone
	**N**	**Patient-time at risk (years)**	**Rate (Per 100 patient-years) (95% CI)**	**N**	**Patient-time at risk (years)**	**Rate (Per 100 patient-years) (95% CI)**	**RR (95% CI)**
Any neurological event	29795	46782	63.7 (63.0–64.4)	39834	47979	83.0 (82.2–83.8)	0.77 (0.75–0.78)
Syncope	9742	55469	17.6 (17.2–17.9)	13189	57826	22.8 (22.4–23.2)	0.77 (0.75–0.79)
Confusional state	5165	62334	8.3 (8.1–8.5)	6790	62905	10.8 (10.5–11.1)	0.77 (0.74–0.79)
Insomnia	6333	60268	10.5 (10.3–10.8)	8665	61943	14.0 (13.7–14.3)	0.75 (0.73–0.77)
Depression	20699	57886	35.8 (35.3–36.3)	26461	59087	44.8 (44.2–45.3)	0.80 (0.78–0.81)

Table S35. Incidence Rates of Musculoskeletal Adverse Events With Intent-to Treat Analysis

	**Dronedarone (N=12,210)**			**Amiodarone (N=12,210)**			**Dronedarone vs Amiodarone**
	**N**	**Patient-time at risk (years)**	**Rate (Per 100 patient-years) (95% CI)**	**N**	**Patient-time at risk (years)**	**Rate (Per 100 patient-years) (95% CI)**	**IRR (95% CI)**
Any musculoskeletal event	3582	37900	9.5 (9.1–9.8)	4313	36541	11.8 (11.5–12.2)	0.80 (0.77–0.84)
Gait disturbance	2986	42653	7.0 (6.8–7.3)	3794	40731	9.3 (9.0–9.6)	0.75 (0.72–0.79)
Rhabdomyolysis	287	54385	0.5 (0.5–0.6)	419	55795	0.8 (0.7–0.8)	0.70 (0.60–0.82)
Myalgia	1213	48799	2.5 (2.4–2.6)	1181	50962	2.3 (2.2–2.5)	1.07 (0.99–1.16)

Table S36. Event Rate of Musculoskeletal Adverse Events With Intent-to Treat Analysis

	**Dronedarone (N=12,210)**			**Amiodarone (N=12,210)**			**Dronedarone vs Amiodarone**
	**N**	**Patient-time at risk (years)**	**Rate (Per 100 patient-years) (95% CI)**	**N**	**Patient-time at risk (years)**	**Rate (Per 100 patient-years) (95% CI)**	**RR (95% CI)**
Any musculoskeletal event	36423	56371	64.6 (64.0–65.3)	44052	56803	77.6 (76.8–78.3)	0.83 (0.82–0.84)
Gait disturbance	32915	59335	55.5 (54.9–56.1)	41575	59549	69.8 (69.2–70.5)	0.79 (0.78–0.80)
Rhabdomyolysis	1755	63867	2.8 (2.6–2.9)	2445	65422	3.7 (3.6–3.9)	0.73 (0.69–0.78)
Myalgia	4225	61188	6.9 (6.7–7.1)	3250	63049	5.2 (5.0–5.3)	1.34 (1.28–1.40)

Table S37. Incidence Rates of Other Adverse Events With Intent-to Treat Analysis

	**Dronedarone (N=12,210)**			**Amiodarone (N=12,210)**			**Dronedarone vs Amiodarone**
	N	Patient-time at risk (years)	Rate (Per 100 patient-years) (95% CI)	N	Patient-time at risk (years)	Rate (Per 100 patient-years) (95% CI)	IRR (95% CI)
Renal and Urinary disorders							
Any renal event	3124	37090	8.4 (8.1–8.7)	4079	34879	11.7 (11.3–12.1)	0.72 (0.69–0.75)
Acute kidney	3100	44085	7.0 (6.8–7.3)	3893	42739	9.1 (8.8–9.4)	0.77 (0.74–0.81)
Renal failure	1623	42127	3.9 (3.7–4.0)	2353	41164	5.7 (5.5–6.0)	0.67 (0.63–0.72)
Blood Disorders			0.0 (0.0–0.0)				
Any blood related event	3065	41113	7.5 (7.2–7.7)	3684	40338	9.1 (8.8–9.4)	0.82 (0.78–0.86)
Anemia	2569	43812	5.9 (5.6–6.1)	3054	43558	7.0 (6.8–7.3)	0.84 (0.79–0.88)
Thrombocytopenia	1137	51051	2.2 (2.1–2.4)	1458	51904	2.8 (2.7–3.0)	0.79 (0.73–0.86)
Eye disorders							
Any ocular event	2024	44961	4.5 (4.3–4.7)	2223	45582	4.9 (4.7–5.1)	0.92 (0.87–0.98)
Blindness	303	54356	0.6 (0.5–0.6)	372	55938	0.7 (0.6–0.7)	0.84 (0.72–0.98)
Visual impairment	477	52696	0.9 (0.8–1.0)	656	53651	1.2 (1.1–1.3)	0.74 (0.66–0.83)
Vision blurred	1573	47585	3.3 (3.1–3.5)	1620	48922	3.3 (3.2–3.5)	1.00 (0.93–1.07)
Skin and subcutaneous tissue disorders							
Any dermatological event	1915	47216	4.1 (3.9–4.2)	1823	48850	3.7 (3.6–3.9)	1.09 (1.02–1.16)
Rash	1523	48875	3.1 (3.0–3.3)	1498	50367	3.0 (2.8–3.1)	1.05 (0.98–1.13)
Pruritus	671	52849	1.3 (1.2–1.4)	617	54578	1.1 (1.0–1.2)	1.12 (1.01–1.25)
Overall							
Any adverse event	623	492	126.5 (116.8–136.9)	724	475	152.4 (141.5–164.0)	0.83 (0.75–0.92)

Table S38. Event Rates of Other Adverse Events With Intent-to Treat Analysis

	**Dronedarone (N=12,210)**			**Amiodarone (N=12,210)**			**Dronedarone vs Amiodarone**
	**N**	**Patient-time at risk (years)**	**Rate (Per 100 patient-years) (95% CI)**	**N**	**Patient-time at risk (years)**	**Rate (Per 100 patient-years) (95% CI)**	**RR (95% CI)**
Renal and Urinary disorders							
Any renal event	33399	53718	62.2 (61.5–62.9)	49769	53696	92.7 (91.9–93.5)	0.67 (0.66–0.68)
Acute kidney	27342	59381	46.1 (45.5–46.6)	34589	59095	58.5 (57.9–59.2)	0.79 (0.77–0.80)
Renal failure	12707	56456	22.5 (22.1–22.9)	23423	57945	40.4 (39.9–40.9)	0.56 (0.54–0.57)
Blood Disorders							
Any blood related event	27275	57744	47.2 (46.7–47.8)	32621	58024	56.2 (55.6–56.8)	0.84 (0.82–0.85)
Anemia	22529	59100	38.1 (37.6–38.6)	27650	59985	46.1 (45.6–46.6)	0.83 (0.81–0.84)
Thrombocytopenia	5799	62470	9.3 (9.1–9.5)	7515	63540	11.8 (11.6–12.1)	0.78 (0.76–0.81)
Eye disorders							
Any ocular event	6374	59972	10.6 (10.4–10.9)	9475	61427	15.4 (15.1–15.7)	0.69 (0.67–0.71)
Blindness	1613	63839	2.5 (2.4–2.7)	1690	65521	2.6 (2.5–2.7)	0.98 (0.91–1.05)
Visual impairment	2137	63033	3.4 (3.3–3.5)	4350	64534	6.7 (6.5–6.9)	0.50 (0.48–0.53)
Vision blurred	3046	61338	5.0 (4.8–5.2)	3835	63044	6.1 (5.9–6.3)	0.81 (0.78–0.85)
Skin and subcutaneous tissue disorders							
Any dermatological event	4072	61347	6.6 (6.4–6.8)	4257	62952	6.8 (6.6–7.0)	0.98 (0.94–1.02)
Rash	2774	62009	4.5 (4.3–4.6)	3006	63524	4.7 (4.6–4.9)	0.94 (0.90–0.99)
Pruritus	1402	63387	2.2 (2.1–2.3)	1371	65095	2.1 (2.0–2.2)	1.05 (0.97–1.13)
Overall							
Any adverse event	20811	3014	690.5 (681.2–700.0)	37266	3624	1,028.4 (1,018.0–1,038.9)	0.66 (0.65–0.67)

Table S39. Incidence Rates of Respiratory Adverse Events in Patients with ≥2 Sequential Prescriptions of the Study Drug

	Dronedarone (N=6,683)			Amiodarone (N=6,683)			Dronedarone vs Amiodarone
	**N**	**Patient-time at risk (years)**	**Rate (Per 100 patient-years) (95% CI)**	**N**	**Patient-time at risk (years)**	**Rate (Per 100 patient-years) (95% CI)**	**IRR (95% CI)**
Any Respiratory event	540	1297	41.6 (38.2–45.3)	768	1192	64.4 (60.0–69.2)	0.65 (0.58–0.72)
Dyspnea	584	2354	24.8 (22.8–26.9)	876	2272	38.6 (36.0–41.2)	0.64 (0.58–0.71)
Interstitial lung	46	4557	1.0 (0.7–1.4)	138	4971	2.8 (2.3–3.3)	0.36 (0.26–0.51)
Cough	488	3322	14.7 (13.4–16.1)	627	3670	17.1 (15.8–18.5)	0.86 (0.76–0.97)
Pulmonary fibrosis	38	4680	0.8 (0.6–1.1)	47	5135	0.9 (0.7–1.2)	0.89 (0.58–1.36)
Pulmonary toxicity	38	4581	0.8 (0.6–1.1)	98	5015	2.0 (1.6–2.4)	0.42 (0.29–0.62)
Respiratory failure	161	4496	3.6 (3.1–4.2)	309	4606	6.7 (6.0–7.5)	0.53 (0.44–0.65)
Lung disorder	380	3000	12.7 (11.4–14.0)	589	3211	18.3 (16.9–19.9)	0.69 (0.61–0.79)
Wheezing	100	4531	2.2 (1.8–2.7)	128	4920	2.6 (2.2–3.1)	0.85 (0.65–1.10)
Pleural effusion	241	4321	5.6 (4.9–6.3)	453	4266	10.6 (9.7–11.6)	0.53 (0.45–0.61)
Acute respiratory	32	4679	0.7 (0.5–1.0)	102	5065	2.0 (1.6–2.4)	0.34 (0.23–0.51)
Pulmonary edema	113	4572	2.5 (2.0–3.0)	276	4801	5.8 (5.1–6.5)	0.43 (0.35–0.54)
Dyspnea exertional	588	2052	28.7 (26.4–31.1)	896	1983	45.2 (42.3–48.2)	0.63 (0.57–0.70)
Pneumonitis	--	4746	--	--	--	--	--
Hypoxia	111	4563	2.4 (2.0–2.9)	217	4877	4.5 (3.9–5.1)	0.55 (0.44–0.69)

Table S40. Event Rates of Respiratory Adverse Events in Patients with ≥2 Sequential Prescriptions of the Study Drug

	Dronedarone (N=6,683)			Amiodarone (N=6,683)			Dronedarone vs Amiodarone
	N	Patient-time at risk (years)	Rate (Per 100 patient-years) (95% CI)	N	Patient-time at risk (years)	Rate (Per 100 patient-years) (95% CI)	IRR (95% CI)
Any Respiratory event	3294	1871	176.0 (170.1–182.1)	7300	1944	375.6 (367.0–384.3)	0.47 (0.45–0.49)
Dyspnea	1466	2954	49.6 (47.1–52.2)	2737	3076	89.0 (85.7–92.4)	0.56 (0.52–0.59)
Interstitial lung	69	4618	1.5 (1.2–1.9)	368	5101	7.2 (6.5–8.0)	0.21 (0.16–0.27)
Cough	910	3858	23.6 (22.1–25.2)	1185	4222	28.1 (26.5–29.7)	0.84 (0.77–0.91)
Pulmonary fibrosis	80	4720	1.7 (1.3–2.1)	93	5181	1.8 (1.5–2.2)	0.94 (0.70–1.27)
Pulmonary toxicity	62	4631	1.3 (1.0–1.7)	374	5123	7.3 (6.6–8.1)	0.18 (0.14–0.24)
Respiratory failure	1153	4605	25.0 (23.6–26.5)	2819	4823	58.5 (56.3–60.6)	0.43 (0.40–0.46)
Lung disorder	1417	3433	41.3 (39.2–43.5)	2877	3791	75.9 (73.1–78.7)	0.54 (0.51–0.58)
Wheezing	149	4654	3.2 (2.7–3.8)	190	5050	3.8 (3.3–4.3)	0.85 (0.69–1.05)
Pleural effusion	1053	4502	23.4 (22.0–24.9)	1809	4653	38.9 (37.1–40.7)	0.60 (0.56–0.65)
Acute respiratory	59	4698	1.3 (1.0–1.6)	326	5141	6.3 (5.7–7.1)	0.20 (0.15–0.26)
Pulmonary edema	200	4663	4.3 (3.7–4.9)	639	5020	12.7 (11.8–13.8)	0.34 (0.29–0.39)
Dyspnea exertional	1611	2671	60.3 (57.4–63.3)	3016	2817	107.1 (103.3–111.0)	0.56 (0.53–0.60)
Pneumonitis	--	--	--	--	--	--	--
Hypoxia	588	4645	12.7 (11.7–13.7)	1626	5055	32.2 (30.6–33.8)	0.39 (0.36–0.43)

Table S41. Incidence Rates of Cardiac and Vascular Adverse Events in Patients with ≥2 Sequential Prescriptions of the Study Drug

	**Dronedarone (N=6,683)**			**Amiodarone (N=6,683)**			**Dronedarone vs Amiodarone**
	**N**	**Patient-time at risk (years)**	**Rate (Per 100 patient-years) (95% CI)**	**N**	**Patient-time at risk (years)**	**Rate (Per 100 patient-years) (95% CI)**	**IRR (95% CI)**
Any Cardiac and Vascular	374	473	79.1 (71.3–87.5)	627	539	116.4 (107.5–125.9)	0.68 (0.60–0.77)
Bradycardia	497	3354	14.8 (13.5–16.2)	745	3650	20.4 (19.0–21.9)	0.73 (0.65–0.81)
Dizziness	380	3357	11.3 (10.2–12.5)	530	3706	14.3 (13.1–15.6)	0.79 (0.69–0.90)
Tachycardia	333	3262	10.2 (9.1–11.4)	369	3774	9.8 (8.8–10.8)	1.04 (0.90–1.21)
Hypotension	267	4072	6.6 (5.8–7.4)	378	4302	8.8 (7.9–9.7)	0.75 (0.64–0.87)
Qt prolonged	36	4690	0.8 (0.5–1.1)	65	5109	1.3 (1.0–1.6)	0.60 (0.40–0.91)
Ventricular tachycardia	275	3820	7.2 (6.4–8.1)	251	4212	6.0 (5.2–6.7)	1.21 (1.02–1.43)
Cardiac failure	235	4346	5.4 (4.7–6.1)	482	4439	10.9 (9.9–11.9)	0.50 (0.43–0.58)
Torsade de pointes	0	4746	--	0	5190	--	--
Cardiac arrest	26	4715	0.6 (0.4–0.8)	62	5106	1.2 (0.9–1.6)	0.45 (0.29–0.72)
Chest pain	449	2062	21.8 (19.8–23.9)	650	2350	27.7 (25.6–29.9)	0.79 (0.70–0.89)
Ventricular fibrillation	21	4688	0.5 (0.3–0.7)	42	5070	0.8 (0.6–1.1)	0.54 (0.32–0.91)
Peripheral edema	459	3597	12.8 (11.6–14.0)	699	3764	18.6 (17.2–20.0)	0.69 (0.61–0.77)
Congestive heart failure	171	4511	3.8 (3.2–4.4)	420	4687	9.0 (8.1–9.9)	0.42 (0.35–0.51)
Arrhythmia	622	2456	25.3 (23.4–27.4)	748	2927	25.6 (23.8–27.5)	0.99 (0.89–1.10)
Cardiogenic shock	14	4734	0.3 (0.2–0.5)	26	5143	0.5 (0.3–0.7)	0.59 (0.31–1.12)
Cardiorespiratory arrest	32	4711	0.7 (0.5–1.0)	65	5098	1.3 (1.0–1.6)	0.53 (0.35–0.81)
Gastrointestinal hemorrhage	200	4360	4.6 (4.0–5.3)	253	4709	5.4 (4.7–6.1)	0.85 (0.71–1.03)

Table S42. Event Rates of Cardiac and Vascular Adverse Events in Patients with ≥2 Sequential Prescriptions of the Study Drug

	Dronedarone (N=6,683)			Amiodarone (N=6,683)			Dronedarone vs Amiodarone
	N	Patient-time at risk (years)	Rate (Per 100 patient-years) (95% CI)	N	Patient-time at risk (years)	Rate (Per 100 patient-years) (95% CI)	RR (95% CI)
Any Cardiac and Vascular	2639	915	288.3 (277.4–299.5)	4963	1166	425.7 (414.0–437.7)	0.67 (0.64–0.71)
Bradycardia	1127	3919	28.8 (27.1–30.5)	1880	4347	43.2 (41.3–45.2)	0.66 (0.62–0.71)
Dizziness	851	3775	22.5 (21.1–24.1)	1196	4229	28.3 (26.7–29.9)	0.80 (0.73–0.87)
Tachycardia	634	3557	17.8 (16.5–19.3)	722	4085	17.7 (16.4–19.0)	1.01 (0.90–1.12)
Hypotension	799	4357	18.3 (17.1–19.7)	1412	4641	30.4 (28.9–32.1)	0.60 (0.55–0.66)
Qt prolonged	69	4731	1.5 (1.1–1.9)	105	5180	2.0 (1.7–2.5)	0.72 (0.53–0.97)
Ventricular tachycardia	708	4100	17.3 (16.0–18.6)	615	4425	13.9 (12.8–15.0)	1.24 (1.11–1.38)
Cardiac failure	1489	4553	32.7 (31.1–34.4)	4060	4889	83.0 (80.5–85.6)	0.39 (0.37–0.42)
Torsade de pointes	0	4752	na	0	5208	na	na
Cardiac arrest	75	4735	1.6 (1.3–2.0)	271	5154	5.3 (4.7–5.9)	0.30 (0.23–0.39)
Chest pain	973	2503	38.9 (36.5–41.4)	1551	2953	52.5 (49.9–55.2)	0.74 (0.68–0.80)
Ventricular fibrillation	58	4711	1.2 (0.9–1.6)	97	5130	1.9 (1.5–2.3)	0.65 (0.47–0.90)
Peripheral edema	1378	4136	33.3 (31.6–35.1)	2197	4438	49.5 (47.5–51.6)	0.67 (0.63–0.72)
Congestive heart failure	1414	4658	30.4 (28.8–32.0)	4586	5061	90.6 (88.0–93.3)	0.33 (0.31–0.35)
Arrhythmia	1402	3132	44.8 (42.5–47.2)	1570	3597	43.7 (41.5–45.9)	1.02 (0.95–1.10)
Cardiogenic shock	86	4747	1.8 (1.5–2.2)	74	5181	1.4 (1.1–1.8)	1.27 (0.93–1.73)
Cardiorespiratory arrest	93	4735	2.0 (1.6–2.4)	276	5149	5.4 (4.8–6.0)	0.37 (0.29–0.46)
Gastrointestinal hemorrhage	753	4565	16.5 (15.3–17.7)	1168	4965	23.5 (22.2–24.9)	0.70 (0.64–0.77)

Table S43. Incidence Rates of General Adverse Events in Patients with ≥2 Sequential Prescriptions of the Study Drug

	**Dronedarone (N=6,683)**			**Amiodarone (N=6,683)**			**Dronedarone vs Amiodarone**
	**N**	**Patient-time at risk (years)**	**Rate (Per 100 patient-years) (95% CI)**	**N**	**Patient-time at risk (years)**	**Rate (Per 100 patient-years) (95% CI)**	**IRR (95% CI)**
Any general event	685	2083	32.9 (30.5–35.5)	995	2129	46.7 (43.9–49.7)	0.70 (0.64–0.78)
Asthenia	469	3030	15.5 (14.1–16.9)	720	3292	21.9 (20.3–23.5)	0.71 (0.63–0.80)
Fatigue	536	2843	18.9 (17.3–20.5)	743	3099	24.0 (22.3–25.8)	0.79 (0.70–0.88)
Weight decreased	139	4446	3.1 (2.6–3.7)	205	4881	4.2 (3.6–4.8)	0.74 (0.60–0.92)
Weight increased	47	4621	1.0 (0.8–1.4)	55	5107	1.1 (0.8–1.4)	0.94 (0.64–1.39)
Malaise	407	3240	12.6 (11.4–13.8)	606	3450	17.6 (16.2–19.0)	0.72 (0.63–0.81)
Edema	466	3592	13.0 (11.8–14.2)	697	3759	18.5 (17.2–20.0)	0.70 (0.62–0.79)

Table S44. Event Rates of General Adverse Events in Patients with ≥2 Sequential Prescriptions of the Study Drug

	**Dronedarone (N=6,683)**			**Amiodarone (N=6,683)**			**Dronedarone vs Amiodarone**
	**N**	**Patient-time at risk (years)**	**Rate (Per 100 patient-years) (95% CI)**	**N**	**Patient-time at risk (years)**	**Rate (Per 100 patient-years) (95% CI)**	**RR (95% CI)**
**Any general event**	3804	2825	134.7 (130.4–139.0)	5661	3078	183.9 (179.2–188.8)	0.73 (0.70–0.76)
Asthenia	1422	3528	40.3 (38.2–42.5)	2184	3937	55.5 (53.2–57.8)	0.73 (0.68–0.78)
Fatigue	1219	3412	35.7 (33.8–37.8)	1871	3817	49.0 (46.8–51.3)	0.73 (0.68–0.78)
Weight decreased	260	4601	5.7 (5.0–6.4)	514	5057	10.2 (9.3–11.1)	0.55 (0.48–0.64)
Weight increased	58	4667	1.2 (0.9–1.6)	87	5163	1.7 (1.4–2.1)	0.74 (0.53–1.03)
Malaise	1129	3661	30.8 (29.1–32.7)	2031	4004	50.7 (48.5–53.0)	0.61 (0.56–0.65)
Edema	1419	4136	34.3 (32.6–36.1)	2209	4431	49.9 (47.8–52.0)	0.69 (0.64–0.73)

Table S45. Incidence Rates of Endocrine and Metabolic Adverse Events in Patients with ≥2 Sequential Prescriptions of the Study Drug

	Dronedarone (N=6,683)			Amiodarone (N=6,683)			Dronedarone vs Amiodarone
	**N**	**Patient-time at risk (years)**	**Rate (Per 100 patient-years) (95% CI)**	**N**	**Patient-time at risk (years)**	**Rate (Per 100 patient-years) (95% CI)**	**IRR (95% CI)**
Any endocrine and metabolic event	293	3098.84	9.5 (8.4–10.6)	607	3337.09	18.2 (16.8–19.7)	0.52 (0.45–0.60)
Hyperthyroidism	47	4604	1.0 (0.8–1.4)	86	5067	1.7 (1.4–2.1)	0.60 (0.42–0.86)
Hypothyroidism	216	3368	6.4 (5.6–7.3)	514	3607	14.3 (13.0–15.5)	0.45 (0.38–0.53)
Thyroid disorder	145	4316	3.4 (2.8–4.0)	196	4812	4.1 (3.5–4.7)	0.83 (0.67–1.02)
Decreased appetite	45	4678	1.0 (0.7–1.3)	88	5075	1.7 (1.4–2.1)	0.55 (0.39–0.79)

Table S46. Event Rates of Endocrine and Metabolic Adverse Events in Patients with ≥2 Sequential Prescriptions of the Study Drug

	Dronedarone (N=6,683)			Amiodarone (N=6,683)			Dronedarone vs Amiodarone
	N	Patient-time at risk (years)	Rate (Per 100 patient-years) (95% CI)	N	Patient-time at risk (years)	Rate (Per 100 patient-years) (95% CI)	RR (95% CI)
Any endocrine and metabolic event	914	3409.1	26.8 (25.1–28.6)	2882	3924.41	73.4 (70.8–76.2)	0.36 (0.34–0.39)
Hyperthyroidism	139	4656	3.0 (2.5–3.5)	231	5140	4.5 (3.9–5.1)	0.66 (0.54–0.82)
Hypothyroidism	573	3605	15.9 (14.6–17.3)	2433	4117	59.1 (56.8–61.5)	0.27 (0.24–0.29)
Thyroid disorder	448	4466	10.0 (9.1–11.0)	474	4987	9.5 (8.7–10.4)	1.05 (0.93–1.20)
Decreased appetite	84	4712	1.8 (1.4–2.2)	188	5150	3.7 (3.2–4.2)	0.49 (0.38–0.63)

Table S47. Incidence Rates of Gastrointestinal and Hepatobiliary Adverse Events in Patients with ≥2 Sequential Prescriptions of the Study Drug

	**Dronedarone (N=6,683)**			**Amiodarone (N=6,683)**			**Dronedarone vs Amiodarone**
	**N**	**Patient-time at risk (years)**	**Rate (Per 100 patient-years) (95% CI)**	**N**	**Patient-time at risk (years)**	**Rate (Per 100 patient-years) (95% CI)**	**IRR (95% CI)**
Any gastrointestinal and hepatobiliary event	602	2932.78	20.5 (18.9–22.2)	733	3092.32	23.7 (22.0–25.5)	0.87 (0.78–0.96)
Nausea	294	4057	7.3 (6.4–8.1)	426	4245	10.0 (9.1–11.0)	0.72 (0.62–0.84)
Vomiting	181	4338	4.2 (3.6–4.8)	290	4541	6.4 (5.7–7.2)	0.65 (0.54–0.79)
Diarrhea	248	4171	6.0 (5.2–6.7)	249	4610	5.4 (4.8–6.1)	1.10 (0.92–1.31)
Abdominal pain	478	3380	14.1 (12.9–15.5)	593	3617	16.4 (15.1–17.8)	0.86 (0.76–0.97)
Liver injury	4	4738	0.1 (0.0–0.2)	1	5188	0.0 (0.0–0.1)	4.38 (0.49–39.19)
Hepatic failure	26	4717	0.6 (0.4–0.8)	52	5146	1.0 (0.8–1.3)	0.55 (0.34–0.87)

Table S48. Event Rates of Gastrointestinal and Hepatobiliary Adverse Events in Patients with ≥2 Sequential Prescriptions of the Study Drug

	**Dronedarone (N=6,683)**			**Amiodarone (N=6,683)**			**Dronedarone vs Amiodarone**
	**N**	**Patient-time at risk (years)**	**Rate (Per 100 patient-years) (95% CI)**	**N**	**Patient-time at risk (years)**	**Rate (Per 100 patient-years) (95% CI)**	**RR (95% CI)**
Any gastrointestinal and hepatobiliary event	2095	3522.99	59.5 (57.0–62.1)	2701	3773.56	71.6 (68.9–74.3)	0.83 (0.78–0.88)
Nausea	621	4351	14.3 (13.2–15.4)	928	4662	19.9 (18.7–21.2)	0.72 (0.65–0.79)
Vomiting	372	4518	8.2 (7.4–9.1)	647	4812	13.4 (12.4–14.5)	0.61 (0.54–0.69)
Diarrhea	558	4434	12.6 (11.6–13.7)	900	4850	18.6 (17.4–19.8)	0.68 (0.61–0.75)
Abdominal pain	1247	3880	32.1 (30.4–34.0)	1449	4154	34.9 (33.1–36.7)	0.92 (0.85–0.99)
Liver injury	13	4746	0.3 (0.2–0.5)	1	5206	0.0 (0.0–0.1)	14.23 (1.86–108.81)
Hepatic failure	128	4738	2.7 (2.3–3.2)	177	5185	3.4 (2.9–4.0)	0.79 (0.63–0.99)

Table S49. Incidence Rates of Neurological Adverse Events in Patients with ≥2 Sequential Prescriptions of the Study Drug

	Dronedarone (N=6,683)			Amiodarone (N=6,683)			Dronedarone vs Amiodarone
	**N**	**Patient-time at risk (years)**	**Rate (Per 100 patient-years) (95% CI)**	**N**	**Patient-time at risk (years)**	**Rate (Per 100 patient-years) (95% CI)**	**IRR (95% CI)**
Any neurological event	458	2997	15.3 (13.9–16.8)	665	3139	21.2 (19.6–22.9)	0.72 (0.64–0.81)
Syncope	225	3864	5.8 (5.1–6.6)	371	4270	8.7 (7.8–9.6)	0.67 (0.57–0.79)
Confusional state	126	4527	2.8 (2.3–3.3)	190	4802	4.0 (3.4–4.6)	0.70 (0.56–0.88)
Insomnia	197	4229	4.7 (4.0–5.4)	289	4629	6.2 (5.5–7.0)	0.75 (0.62–0.89)
Depression	248	4044	6.1 (5.4–7.0)	318	4278	7.4 (6.6–8.3)	0.83 (0.70–0.97)

Table S50. Event Rates of Neurological Adverse Events in Patients with ≥2 Sequential Prescriptions of the Study Drug

	**Dronedarone (N=6,683)**			**Amiodarone (N=6,683)**			**Dronedarone vs Amiodarone**
	**N**	**Patient-time at risk (years)**	**Rate (Per 100 patient-years) (95% CI)**	**N**	**Patient-time at risk (years)**	**Rate (Per 100 patient-years) (95% CI)**	**RR (95% CI)**
Any neurological event	2058	3492	58.9 (56.4–61.5)	3171	3755	84.5 (81.5–87.4)	0.70 (0.66–0.74)
Syncope	665	4070	16.3 (15.1–17.6)	1336	4603	29.0 (27.5–30.6)	0.56 (0.51–0.62)
Confusional state	397	4620	8.6 (7.8–9.5)	757	4966	15.3 (14.2–16.4)	0.56 (0.50–0.64)
Insomnia	466	4468	10.4 (9.5–11.4)	791	4910	16.1 (15.0–17.3)	0.65 (0.58–0.72)
Depression	1247	4296	29.0 (27.4–30.7)	1674	4571	36.6 (34.9–38.4)	0.79 (0.74–0.85)

Table S51. Incidence Rates of Musculoskeletal Adverse Events in Patients with ≥2 Sequential Prescriptions of the Study Drug

	**Dronedarone (N=6,683)**			**Amiodarone (N=6,683)**			**Dronedarone vs Amiodarone**
	**N**	**Patient-time at risk (years)**	**Rate (Per 100 patient-years) (95% CI)**	**N**	**Patient-time at risk (years)**	**Rate (Per 100 patient-years) (95% CI)**	**IRR (95% CI)**
Any musculoskeletal event	371	3831	9.7 (8.7–10.7)	526	3982	13.2 (12.1–14.4)	0.73 (0.64–0.84)
Gait disturbance	272	4126	6.6 (5.8–7.4)	441	4274	10.3 (9.4–11.3)	0.64 (0.55–0.74)
Rhabdomyolysis	22	4708	0.5 (0.3–0.7)	56	5101	1.1 (0.8–1.4)	0.43 (0.26–0.70)
Myalgia	123	4408	2.8 (2.3–3.3)	128	4834	2.7 (2.2–3.2)	1.05 (0.82–1.35)

Table S52. Event Rates of Musculoskeletal Adverse Events in Patients with ≥2 Sequential Prescriptions of the Study Drug

	**Dronedarone (N=6,683)**			**Amiodarone (N=6,683)**			**Dronedarone vs Amiodarone**
	**N**	**Patient-time at risk (years)**	**Rate (Per 100 patient-years) (95% CI)**	**N**	**Patient-time at risk (years)**	**Rate (Per 100 patient-years) (95% CI)**	**RR (95% CI)**
Any musculoskeletal event	2363	4195	56.3 (54.1–58.6)	5028	4493	111.9 (108.8–115.1)	0.50 (0.48–0.53)
Gait disturbance	2090	4396	47.6 (45.5–49.6)	4620	4708	98.1 (95.3–101.0)	0.48 (0.46–0.51)
Rhabdomyolysis	37	4731	0.8 (0.6–1.1)	279	5154	5.4 (4.8–6.1)	0.14 (0.10–0.20)
Myalgia	324	4550	7.1 (6.4–7.9)	310	4975	6.2 (5.6–7.0)	1.14 (0.98–1.33)

Table S53. Incidence Rates of Other Adverse Events in Patients with ≥2 Sequential Prescriptions of the Study Drug

	Dronedarone (N=6,683)			Amiodarone (N=6,683)			Dronedarone vs Amiodarone
	N	Patient-time at risk (years)	Rate (Per 100 patient-years) (95% CI)	N	Patient-time at risk (years)	Rate (Per 100 patient-years) (95% CI)	IRR (95% CI)
Renal and Urinary disorders							
Any renal event	316	3770	8.4 (7.5–9.4)	540	3904	13.8 (12.7–15.1)	0.61 (0.53–0.70)
Acute kidney	275	4217	6.5 (5.8–7.3)	452	4374	10.3 (9.4–11.3)	0.63 (0.54–0.73)
Renal failure	169	4043	4.2 (3.6–4.9)	332	4347	7.6 (6.8–8.5)	0.55 (0.45–0.66)
Blood Disorders			0.0 (0.0–0.0)			0.0 (0.0–0.0)	
Any blood related event	336	3992	8.4 (7.5–9.4)	474	4161	11.4 (10.4–12.5)	0.74 (0.64–0.85)
Anemia	284	4153	6.8 (6.1–7.7)	397	4359	9.1 (8.2–10.1)	0.75 (0.64–0.87)
Thrombocytopenia	113	4515	2.5 (2.1–3.0)	128	4923	2.6 (2.2–3.1)	0.96 (0.75–1.24)
Eye disorders			0.0 (0.0–0.0)			0.0 (0.0–0.0)	
Any ocular event	195	4223	4.6 (4.0–5.3)	265	4661	5.7 (5.0–6.4)	0.81 (0.68–0.98)
Blindness	20	4712	0.4 (0.3–0.7)	43	5135	0.8 (0.6–1.1)	0.51 (0.30–0.86)
Visual impairment	41	4628	0.9 (0.6–1.2)	90	5000	1.8 (1.5–2.2)	0.49 (0.34–0.71)
Vision blurred	152	4335	3.5 (3.0–4.1)	170	4882	3.5 (3.0–4.1)	1.01 (0.81–1.25)
Any dermatological event	211	4342	4.9 (4.2–5.6)	198	4830	4.1 (3.6–4.7)	1.19 (0.98–1.44)
Rash	173	4418	3.9 (3.4–4.5)	171	4890	3.5 (3.0–4.1)	1.12 (0.91–1.38)
Pruritus	54	4635	1.2 (0.9–1.5)	43	5114	0.8 (0.6–1.1)	1.39 (0.93–2.07)
Overall							
Any adverse event	126	98	129.0 (107.4–153.6)	188	117	161.2 (139.0–185.9)	0.80 (0.64–1.00)

Table S54. Event Rates of Other Adverse Events in Patients with ≥2 Sequential Prescriptions of the Study Drug

	**Dronedarone (N=6,683)**			**Amiodarone (N=6,683)**			**Dronedarone vs Amiodarone**
	**N**	**Patient-time at risk (years)**	**Rate (Per 100 patient-years) (95% CI)**	**N**	**Patient-time at risk (years)**	**Rate (Per 100 patient-years) (95% CI)**	**RR (95% CI)**
Any renal event	2010	4103	49.0 (46.9–51.2)	4420	4398	100.5 (97.6–103.5)	0.49 (0.46–0.51)
Acute kidney	1480	4461	33.2 (31.5–34.9)	2651	4705	56.3 (54.2–58.5)	0.59 (0.55–0.63)
Renal failure	884	4261	20.8 (19.4–22.2)	2416	4731	51.1 (49.1–53.1)	0.41 (0.38–0.44)
Blood Disorders							
Any blood related event	1360	4315	31.5 (29.9–33.2)	2035	4598	44.3 (42.4–46.2)	0.71 (0.66–0.76)
Anemia	1109	4419	25.1 (23.6–26.6)	1785	4741	37.7 (35.9–39.4)	0.67 (0.62–0.72)
Thrombocytopenia	329	4618	7.1 (6.4–7.9)	388	5037	7.7 (7.0–8.5)	0.92 (0.80–1.07)
Eye disorders							
Any ocular event	337	4459	7.6 (6.8–8.4)	980	4912	20.0 (18.7–21.2)	0.38 (0.33–0.43)
Blindness	43	4739	0.9 (0.7–1.2)	247	5185	4.8 (4.2–5.4)	0.19 (0.14–0.26)
Visual impairment	86	4680	1.8 (1.5–2.3)	518	5084	10.2 (9.3–11.1)	0.18 (0.14–0.23)
Vision blurred	224	4531	4.9 (4.3–5.6)	360	5054	7.1 (6.4–7.9)	0.69 (0.59–0.82)
Any dermatological event	351	4538	7.7 (7.0–8.6)	397	5012	7.9 (7.2–8.7)	0.97 (0.84–1.13)
Rash	281	4587	6.1 (5.4–6.9)	269	5051	5.3 (4.7–6.0)	1.15 (0.97–1.36)
Pruritus	79	4692	1.7 (1.3–2.1)	134	5164	2.6 (2.2–3.1)	0.65 (0.49–0.86)
Overall							
Any adverse event	1310	239	548.3 (519.0–578.8)	3858	320	1,207.3 (1,169.5–1,246.0)	0.45 (0.42–0.48)

Table S55. Incidence Rates of Respiratory Related Adverse Events in patients with Congestive Heart Failure (Stages I, II and III)

	Dronedarone (N=353)			Amiodarone (N=353)			Dronedarone vs Amiodarone
	**N**	**Patient-time at risk (years)**	**Rate (Per 100 patient-years) (95% CI)**	**N**	**Patient-time at risk (years)**	**Rate (Per 100 patient-years) (95% CI)**	**IRR (95% CI)**
Any respiratory event	20	19	104.1 (63.6–160.7)	30	23	129.7 (87.5–185.1)	0.80 (0.46–1.41)
Dyspnea	25	46	54.4 (35.2–80.2)	47	58	81.0 (59.5–107.7)	0.67 (0.41–1.09)
Interstitial lung	5	170	2.9 (1.0–6.9)	7	206	3.4 (1.4–7.0)	0.86 (0.27–2.73)
Cough	32	114	28.1 (19.2–39.7)	53	150	35.2 (26.4–46.1)	0.80 (0.51–1.24)
Pulmonary fibrosis	8	168	4.8 (2.1–9.4)	11	205	5.4 (2.7–9.6)	0.89 (0.36–2.21)
Pulmonary toxicity	<5	176	--	--	--	--	--
Respiratory failure	30	131	22.9 (15.4–32.7)	29	148	19.6 (13.2–28.2)	1.16 (0.70–1.94)
Lung disorder	22	68	32.6 (20.4–49.3)	30	93	32.1 (21.7–45.8)	1.01 (0.59–1.76)
Wheezing	9	159	5.7 (2.6–10.7)	12	199	6.0 (3.1–10.6)	0.93 (0.39–2.22)
Pleural effusion	34	130	26.1 (18.1–36.4)	30	143	21.0 (14.2–30.0)	1.24 (0.76–2.03)
Acute respiratory	12	171	7.0 (3.6–12.3)	<5	203	2.0 (0.5–5.1)	3.57 (1.15–11.06)
Pulmonary edema	19	145	13.1 (7.9–20.4)	26	179	14.6 (9.5–21.3)	0.90 (0.50–1.62)
Dyspnea exertional	25	45	55.3 (35.8–81.7)	47	58	81.0 (59.5–107.7)	0.68 (0.42–1.11)
Pneumonitis	<5	176	--	0	216	--	--
Hypoxia	24	120	20.0 (12.8–29.8)	36	149	24.2 (16.9–33.5)	0.83 (0.49–1.39)

Table S56. Event Rates of Respiratory Related Adverse Events in patients with Congestive Heart Failure (Stages I, II and III)

	Dronedarone (N=353)			Amiodarone (N=353)			Dronedarone vs Amiodarone
	N	Patient-time at risk (years)	Rate (Per 100 patient-years) (95% CI)	N	Patient-time at risk (years)	Rate (Per 100 patient-years) (95% CI)	RR (95% CI)
Any respiratory event	589	34	1,735.9 (1,598.5–1,881.9)	409	53	769.4 (696.6–847.7)	2.26 (1.99–2.56)
Dyspnea	83	60	137.7 (109.7–170.7)	168	102	164.2 (140.3–191.0)	0.84 (0.64–1.09)
Interstitial lung	11	173	6.3 (3.2–11.4)	9	210	4.3 (2.0–8.1)	1.48 (0.61–3.58)
Cough	64	130	49.4 (38.0–63.0)	95	185	51.5 (41.7–62.9)	0.96 (0.70–1.32)
Pulmonary fibrosis	16	172	9.3 (5.3–15.1)	15	212	7.1 (4.0–11.7)	1.32 (0.65–2.66)
Pulmonary toxicity	--	--	--	--	--	--	--
Respiratory failure	696	142	489.4 (453.7–527.1)	133	164	81.3 (68.0–96.3)	6.02 (5.00–7.25)
Lung disorder	326	83	393.8 (352.2–438.9)	150	110	136.4 (115.5–160.1)	2.89 (2.38–3.50)
Wheezing	24	165	14.6 (9.3–21.7)	16	208	7.7 (4.4–12.5)	1.89 (1.01–3.57)
Pleural effusion	118	147	80.3 (66.5–96.2)	86	166	51.7 (41.3–63.8)	1.55 (1.18–2.05)
Acute respiratory	52	175	29.7 (22.2–39.0)	6	206	2.9 (1.1–6.3)	10.21 (4.38–23.77)
Pulmonary edema	25	158	15.8 (10.3–23.4)	80	195	41.1 (32.6–51.2)	0.39 (0.25–0.60)
Dyspnea exertional	123	60	204.1 (169.6–243.5)	169	102	165.1 (141.2–192.0)	1.24 (0.98–1.56)
Pneumonitis	<5	176	--	<5	216	--	--
Hypoxia	158	130	121.6 (103.3–142.1)	164	167	98.3 (83.9–114.6)	1.24 (0.99–1.54)

Table S57. Incidence Rates of Cardiac and Vascular Related Adverse Events in patients with Congestive Heart Failure (Stages I, II and III)

	**Dronedarone (N=353)**			**Amiodarone (N=353)**			**Dronedarone vs Amiodarone**
	**N**	**Patient-time at risk (years)**	**Rate (Per 100 patient-years) (95% CI)**	**N**	**Patient-time at risk (years)**	**Rate (Per 100 patient-years) (95% CI)**	**IRR (95% CI)**
Any cardiac and vascular event	6	2	363.8 (133.5–791.8)	9	2	384.2 (175.7–729.4)	0.95 (0.34–2.66)
Bradycardia	9	140	6.5 (3.0–12.2)	25	153	16.3 (10.6–24.1)	0.40 (0.18–0.85)
Dizziness	22	117	18.9 (11.8–28.5)	19	143	13.3 (8.0–20.7)	1.42 (0.77–2.63)
Tachycardia	24	93	25.8 (16.5–38.4)	28	104	27.0 (17.9–39.0)	0.96 (0.55–1.65)
Hypotension	26	128	20.3 (13.2–29.7)	34	149	22.8 (15.8–31.8)	0.89 (0.53–1.48)
Qt prolonged	<5	170	2.4 (0.6–6.0)	6	212	2.8 (1.0–6.2)	0.83 (0.23–2.94)
Ventricular tachycardia	13	139	9.3 (5.0–16.0)	11	137	8.0 (4.0–14.3)	1.17 (0.52–2.60)
Cardiac failure	29	29	101.3 (67.8–145.4)	34	26	133.2 (92.2–186.1)	0.76 (0.46–1.25)
Torsade de pointes	--	--	--	--	--	--	--
Cardiac arrest	<5	172	1.2 (0.1–4.2)	8	202	4.0 (1.7–7.8)	0.29 (0.06–1.38)
Chest pain	29	68	42.7 (28.6–61.3)	30	95	31.7 (21.4–45.3)	1.34 (0.81–2.24)
Ventricular fibrillation	<5	174	1.2 (0.1–4.2)	10	192	5.2 (2.5–9.6)	0.22 (0.05–1.01)
Peripheral edema	32	107	30.0 (20.5–42.3)	37	136	27.2 (19.1–37.5)	1.10 (0.69–1.77)
Arrhythmia	29	99	29.4 (19.7–42.2)	21	136	15.5 (9.6–23.6)	1.90 (1.08–3.33)
Cardiogenic shock	<5	175	--	6	205	2.9 (1.1–6.4)	--
Cardiorespiratory arrest	<5	172	1.2 (0.1–4.2)	8	202	4.0 (1.7–7.8)	0.29 (0.06–1.38)
Gastrointestinal hemorrhage	23	144	15.9 (10.1–23.9)	20	184	10.9 (6.6–16.8)	1.47 (0.81–2.67)

Table S58. Event Rates of Cardiac and Vascular Related Adverse Events in patients with Congestive Heart Failure (Stages I, II and III)

	Dronedarone (N=353)			Amiodarone (N=353)			Dronedarone vs Amiodarone
	**N**	**Patient-time at risk (years)**	**Rate (Per 100 patient-years) (95% CI)**	**N**	**Patient-time at risk (years)**	**Rate (Per 100 patient-years) (95% CI)**	**RR (95% CI)**
Any cardiac and vascular event	49	6	442.8 (623.5–1114.3)	202	9	2295.5 (1989.8–2637.8)	0.37 (0.27–0.50)
Bradycardia	11	144	7.7 (3.8–13.7)	59	172	34.4 (26.2–44.3)	0.22 (0.12–0.42)
Dizziness	142	134	106.0 (89.3–125.0)	21	155	13.6 (8.4–20.7)	7.82 (4.95–12.37)
Tachycardia	38	104	36.4 (25.8–50.0)	80	125	64.0 (50.8–79.7)	0.57 (0.39–0.84)
Hypotension	112	138	81.2 (66.9–97.7)	129	176	73.2 (61.1–87.0)	1.11 (0.86–1.43)
Qt prolonged	6	172	3.5 (1.3–7.6)	6	216	2.8 (1.0–6.0)	1.26 (0.41–3.90)
Ventricular tachycardia	35	147	23.8 (16.6–33.0)	114	148	76.9 (63.5–92.4)	0.31 (0.21–0.45)
Cardiac failure	139	42	331.2 (278.5–391.1)	376	53	715.4 (644.9–791.4)	0.46 (0.38–0.56)
Torsade de pointes	--	--	--	--	--	--	--
Cardiac arrest	92	173	53.2 (42.9–65.3)	26	205	12.7 (8.3–18.6)	4.20 (2.72–6.50)
Chest pain	45	83	54.5 (39.8–73.0)	77	120	64.2 (50.7–80.3)	0.85 (0.59–1.23)
Ventricular fibrillation	42	175	24.0 (17.3–32.5)	24	202	11.9 (7.6–17.7)	2.03 (1.23–3.34)
Peripheral edema	77	128	60.1 (47.4–75.1)	95	163	58.4 (47.2–71.3)	1.03 (0.76–1.39)
Arrhythmia	103	121	85.3 (69.7–103.5)	37	150	24.6 (17.3–33.9)	3.47 (2.38–5.05)
Cardiogenic shock	<5	175	--	16	208	7.7 (4.4–12.5)	--
Cardiorespiratory arrest	92	173	53.2 (42.9–65.3)	26	205	12.7 (8.3–18.6)	4.20 (2.72–6.50)
Gastrointestinal hemorrhage	133	156	85.3 (71.4–101.0)	78	201	38.9 (30.7–48.5)	2.19 (1.66–2.90)

Table S59. Incidence Rates of General Adverse Events in patients with Congestive Heart Failure (Stages I, II and III)

	**Dronedarone (N=353)**			**Amiodarone (N=353)**			**Dronedarone vs Amiodarone**
	**N**	**Patient-time at risk (years)**	**Rate (Per 100 patient-years) (95% CI)**	**N**	**Patient-time at risk (years)**	**Rate (Per 100 patient-years) (95% CI)**	**IRR (95% CI)**
Any general event	26	49	53.0 (34.6–77.7)	39	65	59.6 (42.4–81.5)	0.89 (0.54–1.46)
Asthenia	37	107	34.6 (24.4–47.7)	36	137	26.3 (18.4–36.4)	1.32 (0.83–2.08)
Fatigue	29	115	25.3 (17.0–36.4)	35	136	25.7 (17.9–35.7)	0.99 (0.60–1.61)
Weight decreased	10	170	5.9 (2.8–10.8)	10	209	4.8 (2.3–8.8)	1.23 (0.51–2.95)
Weight increased	<5	172	2.3 (0.6–5.9)	<5	214	0.9 (0.1–3.4)	2.48 (0.45–13.56)
Malaise	16	157	10.2 (5.8–16.5)	24	162	14.8 (9.5–22.0)	0.69 (0.37–1.29)
Edema	32	107	30.0 (20.5–42.3)	39	132	29.5 (21.0–40.3)	1.02 (0.64–1.62)

Table S60. Event Rates of General Adverse Events in patients with Congestive Heart Failure (Stages I, II and III)

	**Dronedarone (N=353)**			**Amiodarone (N=353)**			**Dronedarone vs Amiodarone**
	**N**	**Patient-time at risk (years)**	**Rate (Per 100 patient-years) (95% CI)**	**N**	**Patient-time at risk (years)**	**Rate (Per 100 patient-years) (95% CI)**	**RR (95% CI)**
Any general event	107	64	168.1 (137.8–203.2)	183	98	186.0 (160.1–215.0)	0.90 (0.71–1.15)
Asthenia	61	126	48.3 (36.9–62.0)	175	162	108.1 (92.6–125.3)	0.45 (0.33–0.60)
Fatigue	49	128	38.3 (28.3–50.6)	76	163	46.7 (36.8–58.4)	0.82 (0.57–1.17)
Weight decreased	32	172	18.6 (12.7–26.2)	14	215	6.5 (3.6–10.9)	2.85 (1.52–5.34)
Weight increased	<5	173	2.3 (0.6–5.9)	<5	215	0.9 (0.1–3.4)	2.48 (0.45–13.55)
Malaise	74	162	45.6 (35.8–57.2)	64	180	35.5 (27.3–45.3)	1.29 (0.92–1.80)
Edema	79	128	61.7 (48.8–76.9)	99	160	61.7 (50.2–75.2)	1.00 (0.74–1.34)

Table S61. Incidence Rates of Endocrine Adverse Events in patients with Congestive Heart Failure (Stages I, II and III)

	**Dronedarone (N=353)**			**Amiodarone (N=353)**			**Dronedarone vs Amiodarone**
	**N**	**Patient-time at risk (years)**	**Rate (Per 100 patient-years) (95% CI)**	**N**	**Patient-time at risk (years)**	**Rate (Per 100 patient-years) (95% CI)**	**IRR (95% CI)**
Any endocrine event	20	109	18.4 (11.2–28.3)	29	131	22.1 (14.8–31.8)	0.83 (0.47–1.47)
Hyperthyroidism	--	164	--	--	--	--	--
Hypothyroidism	18	115	15.7 (9.3–24.8)	23	143	16.1 (10.2–24.1)	0.98 (0.53–1.81)
Thyroid disorder	8	166	4.8 (2.1–9.5)	12	189	6.4 (3.3–11.1)	0.76 (0.31–1.85)
Decreased appetite	<5	175	2.3 (0.6–5.8)	6	207	2.9 (1.1–6.3)	0.79 (0.22–2.79)

Table S62. Event Rates of Endocrine Adverse Events in patients with Congestive Heart Failure (Stages I, II and III)

	**Dronedarone (N=353)**			**Amiodarone (N=353)**			**Dronedarone vs Amiodarone**
	**N**	**Patient-time at risk (years)**	**Rate (Per 100 patient-years) (95% CI)**	**N**	**Patient-time at risk (years)**	**Rate (Per 100 patient-years) (95% CI)**	**RR (95% CI)**
Any endocrine event	42	115	36.6 (26.4–49.5)	106	154	68.9 (56.4–83.3)	0.53 (0.37–0.76)
Hyperthyroidism	--	--	--	--	--	--	--
Hypothyroidism	36	121	29.9 (20.9–41.3)	96	161	59.6 (48.3–72.8)	0.50 (0.34–0.73)
Thyroid disorder	10	168	6.0 (2.9–11.0)	12	197	6.1 (3.1–10.6)	0.98 (0.42–2.27)
Decreased appetite	6	176	3.4 (1.3–7.4)	6	210	2.9 (1.1–6.2)	1.19 (0.38–3.69)

Table S63. Incidence Rates of Gastrointestinal Adverse Events in patients with Congestive Heart Failure (Stages I, II and III)

	**Dronedarone (N=353)**			**Amiodarone (N=353)**			**Dronedarone vs Amiodarone**
	**N**	**Patient-time at risk (years)**	**Rate (Per 100 patient-years) (95% CI)**	**N**	**Patient-time at risk (years)**	**Rate (Per 100 patient-years) (95% CI)**	**IRR (95% CI)**
Any gastrointestinal event	18	104	17.2 (10.2–27.3)	29	108	26.9 (18.0–38.6)	0.64 (0.36–1.16)
Nausea	15	140	10.7 (6.0–17.7)	19	156	12.1 (7.3–19.0)	0.88 (0.45–1.74)
Vomiting	17	150	11.4 (6.6–18.2)	7	170	4.1 (1.7–8.5)	2.75 (1.14–6.63)
Diarrhea	12	156	7.7 (4.0–13.4)	19	168	11.3 (6.8–17.7)	0.68 (0.33–1.40)
Abdominal pain	13	125	10.4 (5.6–17.8)	25	146	17.1 (11.1–25.3)	0.61 (0.31–1.19)
Liver injury	--	176	--	--	--	--	--
Hepatic failure	--	176	--	--	--	--	--

Table S64. Event Rates of Gastrointestinal Adverse Events in patients with Congestive Heart Failure (Stages I, II and III)

	**Dronedarone (N=353)**			**Amiodarone (N=353)**			**Dronedarone vs Amiodarone**
	**N**	**Patient-time at risk (years)**	**Rate (Per 100 patient-years) (95% CI)**	**N**	**Patient-time at risk (years)**	**Rate (Per 100 patient-years) (95% CI)**	**RR (95% CI)**
Any gastrointestinal event	121	117	103.6 (85.9–123.7)	203	131	155.0 (134.4–177.9)	0.67 (0.53–0.84)
Nausea	48	149	32.1 (23.7–42.6)	75	172	43.6 (34.3–54.6)	0.74 (0.51–1.06)
Vomiting	48	159	30.2 (22.3–40.0)	39	178	21.9 (15.6–30.0)	1.38 (0.90–2.10)
Diarrhea	46	166	27.8 (20.3–37.0)	87	178	49.0 (39.3–60.5)	0.57 (0.40–0.81)
Abdominal pain	57	131	43.4 (32.9–56.3)	37	163	22.7 (16.0–31.2)	1.92 (1.27–2.90)
Liver injury	--	--	--	--	--	--	--
Hepatic failure	--	--	--	--	--	--	--

Table S65. Incidence Rates of Neurological Adverse Events in patients with Congestive Heart Failure (Stages I, II and III)

	**Dronedarone (N=353)**			**Amiodarone (N=353)**			**Dronedarone vs Amiodarone**
	**N**	**Patient-time at risk (years)**	**Rate (Per 100 patient-years) (95% CI)**	**N**	**Patient-time at risk (years)**	**Rate (Per 100 patient-years) (95% CI)**	**IRR (95% CI)**
Any neurological event	30	86	35.1 (23.7–50.0)	32	82	39.0 (26.7–55.1)	0.90 (0.55–1.48)
Syncope	21	136	15.4 (9.5–23.6)	17	151	11.3 (6.6–18.1)	1.37 (0.72–2.59)
Confusional state	6	167	3.6 (1.3–7.8)	17	196	8.7 (5.0–13.9)	0.41 (0.16–1.05)
Insomnia	13	141	9.2 (4.9–15.7)	22	171	12.8 (8.0–19.4)	0.72 (0.36–1.42)
Depression	21	121	17.3 (10.7–26.5)	21	147	14.3 (8.8–21.8)	1.21 (0.66–2.22)

Table S66. Event Rates of Neurological Adverse Events in patients with Congestive Heart Failure (Stages I, II and III)

	Dronedarone (N=353)			Amiodarone (N=353)			Dronedarone vs Amiodarone
	**N**	**Patient-time at risk (years)**	**Rate (Per 100 patient-years) (95% CI)**	**N**	**Patient-time at risk (years)**	**Rate (Per 100 patient-years) (95% CI)**	**RR (95% CI)**
Any neurological event	132	99	133.0 (111.3–157.7)	308	104	295.3 (263.2–330.2)	0.45 (0.37–0.55)
Syncope	57	143	39.8 (30.2–51.6)	36	163	22.1 (15.5–30.6)	1.80 (1.19–2.74)
Confusional state	12	169	7.1 (3.7–12.4)	37	208	17.8 (12.5–24.5)	0.40 (0.21–0.77)
Insomnia	17	148	11.5 (6.7–18.4)	44	185	23.8 (17.3–32.0)	0.48 (0.28–0.85)
Depression	128	134	95.8 (79.9–113.9)	289	161	179.4 (159.3–201.3)	0.53 (0.43–0.66)

Table S67. Incidence Rates of Musculoskeletal Adverse Events in patients with Congestive Heart Failure (Stages I, II and III)

	**Dronedarone (N=353)**			**Amiodarone (N=353)**			**Dronedarone vs Amiodarone**
	**N**	**Patient-time at risk (years)**	**Rate (Per 100 patient-years) (95% CI)**	**N**	**Patient-time at risk (years)**	**Rate (Per 100 patient-years) (95% CI)**	**IRR (95% CI)**
Any musculoskeletal event	28	126	22.2 (14.8–32.1)	27	154	17.6 (11.6–25.6)	1.26 (0.74–2.14)
Gait disturbance	24	137	17.5 (11.2–26.0)	19	164	11.6 (7.0–18.1)	1.50 (0.82–2.75)
Rhabdomyolysis	<5	175	1.1 (0.1–4.1)	8	212	3.8 (1.6–7.5)	0.30 (0.06–1.42)
Myalgia	<5	165	2.4 (0.7–6.2)	<5	208	1.9 (0.5–4.9)	1.26 (0.32–5.04)

Table S68. Event Rates of Musculoskeletal Adverse Events in patients with Congestive Heart Failure (Stages I, II and III)

	**Dronedarone (N=353)**			**Amiodarone (N=353)**			**Dronedarone vs Amiodarone**
	**N**	**Patient-time at risk (years)**	**Rate (Per 100 patient-years) (95% CI)**	**N**	**Patient-time at risk (years)**	**Rate (Per 100 patient-years) (95% CI)**	**RR (95% CI)**
Any musculoskeletal event	126	146	86.3 (71.9–102.7)	117	170	68.8 (56.9–82.5)	1.25 (0.97–1.61)
Gait disturbance	112	151	74.0 (60.9–89.0)	89	176	50.7 (40.7–62.3)	1.46 (1.11–1.93)
Rhabdomyolysis	<5	176	1.1 (0.1–4.1)	22	215	10.2 (6.4–15.5)	0.11 (0.03–0.47)
Myalgia	12	171	7.0 (3.6–12.3)	6	211	2.9 (1.0–6.2)	2.46 (0.92–6.56)

Table S69. Incidence Rates of Other Adverse Events in patients with Congestive Heart Failure (Stages I, II and III)

	Dronedarone (N=353)			Amiodarone (N=353)			Dronedarone vs Amiodarone
	N	Patient-time at risk (years)	Rate (Per 100 patient-years) (95% CI)	N	Patient-time at risk (years)	Rate (Per 100 patient-years) (95% CI)	IRR (95% CI)
Renal and Urinary disorders							
Any renal event	29	106	27.3 (18.3–39.3)	37	122	30.3 (21.4–41.8)	0.90 (0.55–1.47)
Acute kidney	29	110	26.5 (17.7–38.0)	35	129	27.2 (18.9–37.8)	0.97 (0.60–1.59)
Renal failure	15	163	9.2 (5.2–15.2)	13	191	6.8 (3.6–11.6)	1.36 (0.65–2.85)
Blood Disorders							
Any blood related event	36	118	30.4 (21.3–42.1)	49	127	38.6 (28.6–51.1)	0.79 (0.51–1.21)
Anemia	28	127	22.0 (14.6–31.8)	47	139	33.8 (24.8–44.9)	0.65 (0.41–1.04)
Thrombocytopenia	14	164	8.5 (4.7–14.3)	12	190	6.3 (3.3–11.0)	1.35 (0.63–2.92)
Eye disorders			0.0 (0.0–0.0)			0.0 (0.0–0.0)	
Any ocular event	13	149	8.8 (4.7–15.0)	10	193	5.2 (2.5–9.5)	1.69 (0.74–3.86)
Blindness	<5	170	1.2 (0.1–4.2)	<5	211	1.0 (0.1–3.4)	1.24 (0.17–8.78)
Visual impairment	<5	171	0.6 (0.0–3.3)	<5	206	1.9 (0.5–5.0)	0.30 (0.03–2.70)
Vision blurred	10	161	6.2 (3.0–11.5)	<5	208	1.9 (0.5–4.9)	3.23 (1.01–10.31)
Skin and subcutaneous tissue disorders							
Any dermatological event	9	142	6.4 (2.9–12.1)	8	187	4.3 (1.9–8.4)	1.48 (0.57–3.85)
Rash	5	147	3.4 (1.1–8.0)	10	197	5.1 (2.4–9.3)	0.67 (0.23–1.97)
Pruritus	<5	168	2.4 (0.7–6.1)	<5	202	2.0 (0.5–5.1)	1.20 (0.30–4.79)
Overall							
Any adverse event	<5	0	688.7 (83.4–2,487.8)	<5	0	--	--

Table S70. Event Rates of Other Adverse Events in patients with Congestive Heart Failure (Stages I, II and III)

	**Dronedarone (N=353)**			**Amiodarone (N=353)**			**Dronedarone vs Amiodarone**
	**N**	**Patient-time at risk (years)**	**Rate (Per 100 patient-years) (95% CI)**	**N**	**Patient-time at risk (years)**	**Rate (Per 100 patient-years) (95% CI)**	**RR (95% CI)**
Renal and Urinary disorders							
Any renal event	628	120	521.4 (481.4–563.8)	196	147	133.2 (115.2–153.3)	3.91 (3.33–4.59)
Acute kidney	528	124	426.0 (390.5–464.0)	188	153	123.2 (106.2–142.1)	3.46 (2.93–4.08)
Renal failure	103	167	61.5 (50.2–74.6)	15	202	7.4 (4.2–12.2)	8.30 (4.83–14.26)
Blood Disorders			0.0 (0.0–0.0)				
Any blood related event	290	138	210.0 (186.5–235.6)	213	162	131.7 (114.6–150.7)	1.59 (1.34–1.90)
Anemia	274	144	189.8 (168.0–213.6)	197	174	113.5 (98.2–130.6)	1.67 (1.39–2.01)
Thrombocytopenia	26	168	15.5 (10.1–22.7)	38	195	19.5 (13.8–26.7)	0.79 (0.48–1.31)
Eye disorders							
Any ocular event	27	158	17.1 (11.3–24.8)	74	199	37.2 (29.2–46.7)	0.46 (0.30–0.71)
Blindness	<5	171	1.2 (0.1–4.2)	66	213	31.0 (24.0–39.4)	0.04 (0.01–0.15)
Visual impairment	7	172	4.1 (1.6–8.4)	<5	209	1.9 (0.5–4.9)	2.12 (0.62–7.26)
Vision blurred	18	168	10.7 (6.4–16.9)	<5	208	1.9 (0.5–4.9)	5.57 (1.89–16.46)
Skin and subcutaneous tissue disorders							
Any dermatological event	9	146	6.2 (2.8–11.7)	56	190	29.6 (22.3–38.4)	0.21 (0.10–0.42)
Rash	5	149	3.4 (1.1–7.8)	12	203	5.9 (3.1–10.3)	0.57 (0.20–1.61)
Pruritus	<5	170	2.4 (0.6–6.0)	48	203	23.6 (17.4–31.4)	0.10 (0.04–0.28)
Overall							
Any adverse event	8	2	486.7 (210.1–958.9)	56	2	2,911.7 (2,199.5–3,781.1)	0.17 (0.08–0.35)

Table S71. Incidence Rates of Respiratory Adverse Events in Patients Without Congestive Heart Failure (Stages I, II and III)

	**Dronedarone (N=9,207)**			**Amiodarone (N=9,207)**			**Dronedarone vs Amiodarone**
	**N**	**Patient-time at risk (years)**	**Rate (Per 100 patient-years) (95% CI)**	**N**	**Patient-time at risk (years)**	**Rate (Per 100 patient-years) (95% CI)**	**IRR (95% CI)**
Any Respiratory event	656	1634	40.1 (37.1–43.3)	751	1261	59.6 (55.4–64.0)	0.67 (0.61–0.75)
Dyspnea	696	2700	25.8 (23.9–27.8)	759	2254	33.7 (31.3–36.2)	0.77 (0.69–0.85)
Interstitial lung	57	5000	1.1 (0.9–1.5)	70	4706	1.5 (1.2–1.9)	0.77 (0.54–1.09)
Cough	520	3770	13.8 (12.6–15.0)	526	3559	14.8 (13.5–16.1)	0.93 (0.83–1.05)
Pulmonary fibrosis	39	5085	0.8 (0.6–1.1)	34	4783	0.7 (0.5–1.0)	1.08 (0.68–1.71)
Pulmonary toxicity	35	5032	0.7 (0.5–1.0)	46	4742	1.0 (0.7–1.3)	0.72 (0.46–1.11)
Respiratory failure	133	4921	2.7 (2.3–3.2)	189	4409	4.3 (3.7–4.9)	0.63 (0.51–0.79)
Lung disorder	429	3519	12.2 (11.1–13.4)	477	3289	14.5 (13.2–15.9)	0.84 (0.74–0.96)
Wheezing	95	4934	1.9 (1.6–2.4)	102	4657	2.2 (1.8–2.7)	0.88 (0.66–1.16)
Pleural effusion	241	4773	5.1 (4.4–5.7)	323	4130	7.8 (7.0–8.7)	0.65 (0.55–0.76)
Acute respiratory	32	5074	0.6 (0.4–0.9)	31	4764	0.7 (0.4–0.9)	0.97 (0.59–1.59)
Pulmonary edema	109	4990	2.2 (1.8–2.6)	169	4535	3.7 (3.2–4.3)	0.59 (0.46–0.75)
Dyspnea exertional	703	2426	29.0 (26.9–31.2)	787	1993	39.5 (36.8–42.4)	0.73 (0.66–0.81)
Pneumonitis	--	--	--	--	--	--	--
Hypoxia	117	4963	2.4 (2.0–2.8)	186	4542	4.1 (3.5–4.7)	0.58 (0.46–0.73)

Table S72. Event Rates of Respiratory Adverse Events in Patients Without Congestive Heart Failure (Stages I, II and III)

	**Dronedarone (N=9,207)**			**Amiodarone (N=9,207)**			**Dronedarone vs Amiodarone**
	**N**	**Patient-time at risk (years)**	**Rate (Per 100 patient-years) (95% CI)**	**N**	**Patient-time at risk (years)**	**Rate (Per 100 patient-years) (95% CI)**	**RR (95% CI)**
Any Respiratory event	3788	2236	169.5 (164.1–174.9)	4376	1906	229.6 (222.9–236.5)	0.74 (0.71–0.77)
Dyspnea	1700	3314	51.3 (48.9–53.8)	1967	2897	67.9 (64.9–71.0)	0.75 (0.71–0.80)
Interstitial lung	121	5063	2.4 (2.0–2.9)	221	4786	4.6 (4.0–5.3)	0.52 (0.41–0.65)
Cough	933	4280	21.8 (20.4–23.2)	845	3982	21.2 (19.8–22.7)	1.03 (0.94–1.13)
Pulmonary fibrosis	75	5121	1.5 (1.2–1.8)	133	4825	2.8 (2.3–3.3)	0.53 (0.40–0.70)
Pulmonary toxicity	68	5075	1.3 (1.0–1.7)	122	4808	2.5 (2.1–3.0)	0.53 (0.39–0.71)
Respiratory failure	957	5023	19.1 (17.9–20.3)	1482	4550	32.6 (30.9–34.3)	0.58 (0.54–0.63)
Lung disorder	1368	3911	35.0 (33.2–36.9)	1561	3722	41.9 (39.9–44.1)	0.83 (0.77–0.90)
Wheezing	128	5052	2.5 (2.1–3.0)	131	4749	2.8 (2.3–3.3)	0.92 (0.72–1.17)
Pleural effusion	837	4930	17.0 (15.9–18.2)	1120	4406	25.4 (24.0–27.0)	0.67 (0.61–0.73)
Acute respiratory	81	5103	1.6 (1.3–2.0)	57	4804	1.2 (0.9–1.5)	1.34 (0.95–1.88)
Pulmonary edema	177	5068	3.5 (3.0–4.1)	365	4687	7.8 (7.0–8.6)	0.45 (0.37–0.54)
Dyspnea exertional	1832	3042	60.2 (57.5–63.1)	2082	2672	77.9 (74.6–81.4)	0.77 (0.72–0.82)
Pneumonitis	--	--	--	--	--	--	--
Hypoxia	651	5059	12.9 (11.9–13.9)	1008	4697	21.5 (20.2–22.8)	0.60 (0.54–0.66)

Table S73. Incidence Rates of Cardiac and Vascular Adverse Events in Patients Without Congestive Heart Failure (Stages I, II and III)

	**Dronedarone (N=9,207)**			**Amiodarone (N=9,207)**			**Dronedarone vs Amiodarone**
	**N**	**Patient-time at risk (years)**	**Rate (Per 100 patient-years) (95% CI)**	**N**	**Patient-time at risk (years)**	**Rate (Per 100 patient-years) (95% CI)**	**IRR (95% CI)**
Any Cardiac and Vascular event	534	614	86.9 (79.7–94.6)	641	611	104.9 (96.9–113.4)	0.83 (0.74–0.93)
Bradycardia	633	3718	17.0 (15.7–18.4)	703	3500	20.1 (18.6–21.6)	0.85 (0.76–0.94)
Dizziness	406	3756	10.8 (9.8–11.9)	475	3645	13.0 (11.9–14.3)	0.83 (0.73–0.95)
Tachycardia	418	3385	12.4 (11.2–13.6)	374	3430	10.9 (9.8–12.1)	1.13 (0.99–1.30)
Hypotension	249	4580	5.4 (4.8–6.2)	239	4218	5.7 (5.0–6.4)	0.96 (0.80–1.15)
Qt prolonged	58	5060	1.2 (0.9–1.5)	70	4747	1.5 (1.2–1.9)	0.78 (0.55–1.10)
Ventricular tachycardia	351	4166	8.4 (7.6–9.4)	260	4000	6.5 (5.7–7.3)	1.30 (1.10–1.52)
Cardiac failure	273	4776	5.7 (5.1–6.4)	406	4294	9.5 (8.6–10.4)	0.60 (0.52–0.71)
Torsade de pointes	--	--	--	--	--	--	--
Cardiac arrest	21	5108	0.4 (0.3–0.6)	33	4790	0.7 (0.5–1.0)	0.60 (0.35–1.03)
Chest pain	561	2395	23.4 (21.5–25.5)	580	2293	25.3 (23.3–27.4)	0.93 (0.82–1.04)
Ventricular fibrillation	16	5073	0.3 (0.2–0.5)	33	4756	0.7 (0.5–1.0)	0.45 (0.25–0.83)
Peripheral edema	434	4153	10.5 (9.5–11.5)	534	3807	14.0 (12.9–15.3)	0.75 (0.66–0.85)
Arrhythmia	738	2816	26.2 (24.4–28.2)	713	2840	25.1 (23.3–27.0)	1.04 (0.94–1.16)
Cardiogenic shock	12	5125	0.2 (0.1–0.4)	20	4816	0.4 (0.3–0.6)	0.56 (0.28–1.15)
Cardiorespiratory arrest	22	5104	0.4 (0.3–0.7)	36	4780	0.8 (0.5–1.0)	0.57 (0.34–0.97)
Gastrointestinal hemorrhage	195	4742	4.1 (3.6–4.7)	165	4537	3.6 (3.1–4.2)	1.13 (0.92–1.39)

Table S74. Event Rates of Cardiac and Vascular Adverse Events in Patients Without Congestive Heart Failure (Stages I, II and III)

	Dronedarone (N=9,207)			Amiodarone (N=9,207)			Dronedarone vs Amiodarone
	**N**	**Patient-time at risk (years)**	**Rate (Per 100 patient-years) (95% CI)**	**N**	**Patient-time at risk (years)**	**Rate (Per 100 patient-years) (95% CI)**	**RR (95% CI)**
Any Cardiac and Vascular event	2976	1150	258.7 (249.5–268.2)	3518	1193	294.9 (285.2–304.8)	0.88 (0.84–0.92)
Bradycardia	1348	4301	31.3 (29.7–33.1)	1593	4108	38.8 (36.9–40.7)	0.81 (0.75–0.87)
Dizziness	962	4147	23.2 (21.8–24.7)	1012	4057	25.0 (23.4–26.5)	0.93 (0.85–1.01)
Tachycardia	841	3746	22.5 (21.0–24.0)	919	3737	24.6 (23.0–26.2)	0.91 (0.83–1.00)
Hypotension	594	4818	12.3 (11.4–13.4)	731	4406	16.6 (15.4–17.8)	0.74 (0.67–0.83)
Qt prolonged	96	5124	1.9 (1.5–2.3)	102	4821	2.1 (1.7–2.6)	0.88 (0.67–1.17)
Ventricular tachycardia	812	4473	18.2 (16.9–19.5)	687	4212	16.3 (15.1–17.6)	1.11 (1.00–1.23)
Cardiac failure	1496	4995	30.0 (28.5–31.5)	2324	4641	50.1 (48.1–52.2)	0.60 (0.56–0.64)
Torsade de pointes	--	--	--	--	--	--	--
Cardiac arrest	34	5129	0.7 (0.5–0.9)	135	4820	2.8 (2.4–3.3)	0.24 (0.16–0.34)
Chest pain	1135	2917	38.9 (36.7–41.2)	1254	2785	45.0 (42.6–47.6)	0.86 (0.80–0.94)
Ventricular fibrillation	36	5096	0.7 (0.5–1.0)	89	4798	1.9 (1.5–2.3)	0.38 (0.26–0.56)
Peripheral edema	1181	4590	25.7 (24.3–27.2)	1466	4271	34.3 (32.6–36.1)	0.75 (0.69–0.81)
Arrhythmia	1482	3506	42.3 (40.1–44.5)	1445	3419	42.3 (40.1–44.5)	1.00 (0.93–1.07)
Cardiogenic shock	32	5141	0.6 (0.4–0.9)	91	4842	1.9 (1.5–2.3)	0.33 (0.22–0.49)
Cardiorespiratory arrest	48	5126	0.9 (0.7–1.2)	138	4812	2.9 (2.4–3.4)	0.33 (0.23–0.45)
Gastrointestinal hemorrhage	765	4945	15.5 (14.4–16.6)	592	4689	12.6 (11.6–13.7)	1.22 (1.10–1.36)

Table S75. Incidence Rates of General AEs Among Dronedarone and Amiodarone Patients Without CHF (Stages I, II and III)

	**Dronedarone (N=9,207)**			**Amiodarone (N=9,207)**			**Dronedarone vs Amiodarone**
	**N**	**Patient-time at risk (years)**	**Rate (Per 100 patient-years) (95% CI)**	**N**	**Patient-time at risk (years)**	**Rate (Per 100 patient-years) (95% CI)**	**IRR (95% CI)**
Any general event	779	2558	30.5 (28.4–32.7)	910	2312	39.4 (36.8–42.0)	0.77 (0.70–0.85)
Asthenia	479	3564	13.4 (12.3–14.7)	563	3380	16.7 (15.3–18.1)	0.81 (0.71–0.91)
Fatigue	588	3302	17.8 (16.4–19.3)	661	3172	20.8 (19.3–22.5)	0.85 (0.76–0.96)
Weight decreased	110	4932	2.2 (1.8–2.7)	131	4629	2.8 (2.4–3.4)	0.79 (0.61–1.02)
Weight increased	43	5046	0.9 (0.6–1.2)	57	4747	1.2 (0.9–1.6)	0.71 (0.48–1.05)
Malaise	410	3756	10.9 (9.9–12.0)	457	3585	12.8 (11.6–14.0)	0.86 (0.75–0.98)
Edema	437	4149	10.5 (9.6–11.6)	533	3799	14.0 (12.9–15.3)	0.75 (0.66–0.85)

Table S76. Event Rates of General Adverse Events in Patients Without Congestive Heart Failure (Stages I, II and III)

	**Dronedarone (N=9,207)**			**Amiodarone (N=9,207)**			**Dronedarone vs Amiodarone**
	**N**	**Patient-time at risk (years)**	**Rate (Per 100 patient-years) (95% CI)**	**N**	**Patient-time at risk (years)**	**Rate (Per 100 patient-years) (95% CI)**	**RR (95% CI)**
Any general event	3115	3290	94.7 (91.4–98.1)	4162	3076	135.3 (131.2–139.5)	0.70 (0.67–0.73)
Asthenia	1006	4006	25.1 (23.6–26.7)	1537	3846	40.0 (38.0–42.0)	0.63 (0.58–0.68)
Fatigue	1146	3868	29.6 (27.9–31.4)	1341	3729	36.0 (34.1–37.9)	0.82 (0.76–0.89)
Weight decreased	224	5030	4.5 (3.9–5.1)	355	4748	7.5 (6.7–8.3)	0.60 (0.50–0.70)
Weight increased	57	5085	1.1 (0.9–1.5)	79	4804	1.6 (1.3–2.1)	0.68 (0.48–0.96)
Malaise	932	4129	22.6 (21.1–24.1)	1176	3974	29.6 (27.9–31.3)	0.76 (0.70–0.83)
Edema	1197	4588	26.1 (24.6–27.6)	1481	4262	34.8 (33.0–36.6)	0.75 (0.69–0.81)

Table S77. Incidence Rates of Endocrine and Metabolic Adverse Events in Patients Without Congestive Heart Failure (Stages I, II and III)

	**Dronedarone (N=9,207)**			**Amiodarone (N=9,207)**			**Dronedarone vs Amiodarone**
	**N**	**Patient-time at risk (years)**	**Rate (Per 100 patient-years) (95% CI)**	**N**	**Patient-time at risk (years)**	**Rate (Per 100 patient-years) (95% CI)**	**IRR (95% CI)**
Any endocrine and metabolic event	315	3493	9.0 (8.1–10.1)	511	3374	15.1 (13.9–16.5)	0.60 (0.52–0.69)
Hyperthyroidism	54	4989	1.1 (0.8–1.4)	78	4715	1.7 (1.3–2.1)	0.65 (0.46–0.93)
Hypothyroidism	225	3760	6.0 (5.2–6.8)	426	3584	11.9 (10.8–13.1)	0.50 (0.43–0.59)
Thyroid disorder	181	4660	3.9 (3.3–4.5)	163	4521	3.6 (3.1–4.2)	1.08 (0.87–1.33)
Decreased appetite	47	5068	0.9 (0.7–1.2)	55	4770	1.2 (0.9–1.5)	0.80 (0.55–1.19)

Table S78. Event Rates of Endocrine and Metabolic Adverse Events in Patients Without Congestive Heart Failure (Stages I, II and III)

	Dronedarone (N=9,207)			Amiodarone (N=9,207)			Dronedarone vs Amiodarone
	**N**	**Patient-time at risk (years)**	**Rate (Per 100 patient-years) (95% CI)**	**N**	**Patient-time at risk (years)**	**Rate (Per 100 patient-years) (95% CI)**	**RR (95% CI)**
Any endocrine and metabolic event	995	3786	26.3 (24.7–28.0)	2050	3825	53.6 (51.3–56.0)	0.49 (0.45–0.53)
Hyperthyroidism	144	5040	2.9 (2.4–3.4)	230	4782	4.8 (4.2–5.5)	0.59 (0.48–0.73)
Hypothyroidism	639	3970	16.1 (14.9–17.4)	1654	3988	41.5 (39.5–43.5)	0.39 (0.35–0.42)
Thyroid disorder	525	4839	10.9 (9.9–11.8)	398	4650	8.6 (7.7–9.4)	1.27 (1.11–1.44)
Decreased appetite	73	5108	1.4 (1.1–1.8)	91	4823	1.9 (1.5–2.3)	0.76 (0.56–1.03)

Table S79. Incidence Rates of Gastrointestinal and Hepatobiliary Adverse Events in Patients Without Congestive Heart Failure (Stages I, II and III)

	Dronedarone (N=9,207)			Amiodarone (N=9,207)			Dronedarone vs Amiodarone
	**N**	**Patient-time at risk (years)**	**Rate (Per 100 patient-years) (95% CI)**	**N**	**Patient-time at risk (years)**	**Rate (Per 100 patient-years) (95% CI)**	**IRR (95% CI)**
Any gastrointestinal and hepatobiliary event	650	3335	19.5 (18.0–21.1)	620	3139	19.8 (18.2–21.4)	0.99 (0.88–1.10)
Nausea	273	4475	6.1 (5.4–6.9)	331	4116	8.0 (7.2–9.0)	0.76 (0.65–0.89)
Vomiting	168	4747	3.5 (3.0–4.1)	198	4392	4.5 (3.9–5.2)	0.79 (0.64–0.96)
Diarrhea	260	4589	5.7 (5.0–6.4)	189	4379	4.3 (3.7–5.0)	1.31 (1.09–1.58)
Abdominal pain	523	3817	13.7 (12.6–14.9)	486	3596	13.5 (12.3–14.8)	1.01 (0.90–1.15)
Liver injury	<5	5123	0.1 (0.0–0.2)	<5	4832	0.0 (0.0–0.2)	1.42 (0.24–8.47)
Hepatic failure	12	5109	0.2 (0.1–0.4)	34	4800	0.7 (0.5–1.0)	0.33 (0.17–0.64)

Table S80. Event Rates of Gastrointestinal and Hepatobiliary Adverse Events in Patients Without Congestive Heart Failure (Stages I, II and III)

	Dronedarone (N=9,207)			Amiodarone (N=9,207)			Dronedarone vs Amiodarone
	**N**	**Patient-time at risk (years)**	**Rate (Per 100 patient-years) (95% CI)**	**N**	**Patient-time at risk (years)**	**Rate (Per 100 patient-years) (95% CI)**	**RR (95% CI)**
Any gastrointestinal and hepatobiliary event	2095	3931	53.3 (51.0–55.6)	2039	3641	56.0 (0.0–53.6)	58.48 (0.95–0.89)
Nausea	593	4746	12.5 (11.5–13.5)	750	4402	17.0 (0.0–15.8)	18.30 (0.73–0.66)
Vomiting	385	4924	7.8 (7.1–8.6)	474	4556	10.4 (0.0–9.5)	11.38 (0.75–0.66)
Diarrhea	580	4861	11.9 (11.0–12.9)	469	4528	10.4 (0.0–9.4)	11.34 (1.15–1.02)
Abdominal pain	1189	4279	27.8 (26.2–29.4)	1017	3973	25.6 (0.0–24.1)	27.22 (1.08–1.00)
Liver injury	13	5135	0.3 (0.1–0.4)	<5	4849	0.0 (0.0–0.0)	0.15 (6.13–1.38)
Hepatic failure	50	5128	1.0 (0.7–1.3)	194	4833	4.0 (0.0–3.5)	4.62 (0.24–0.18)

Table S81. Incidence Rates of neurological Adverse Events in Patients Without Congestive Heart Failure (Stages I, II and III)

	**Dronedarone (N=9,207)**			**Amiodarone (N=9,207)**			**Dronedarone vs Amiodarone**
	**N**	**Patient-time at risk (years)**	**Rate (Per 100 patient-years) (95% CI)**	**N**	**Patient-time at risk (years)**	**Rate (Per 100 patient-years) (95% CI)**	**IRR (95% CI)**
Any neurological event	494	3417	14.5 (13.2–15.8)	529	3243	16.3 (15.0–17.8)	0.89 (0.78–1.00)
Syncope	252	4345	5.8 (5.1–6.6)	238	4179	5.7 (5.0–6.5)	1.02 (0.85–1.22)
Confusional state	70	4977	1.4 (1.1–1.8)	99	4624	2.1 (1.7–2.6)	0.66 (0.48–0.89)
Insomnia	209	4612	4.5 (3.9–5.2)	236	4391	5.4 (4.7–6.1)	0.84 (0.70–1.02)
Depression	243	4422	5.5 (4.8–6.2)	262	4106	6.4 (5.6–7.2)	0.86 (0.72–1.03)

Table S82. Event Rates of Neurological Adverse Events in Patients Without Congestive Heart Failure (Stages I, II and III)

	Dronedarone (N=9,207)			Amiodarone (N=9,207)			Dronedarone vs Amiodarone
	**N**	**Patient-time at risk (years)**	**Rate (Per 100 patient-years) (95% CI)**	**N**	**Patient-time at risk (years)**	**Rate (Per 100 patient-years) (95% CI)**	**RR (95% CI)**
Any neurological event	1416	3889	36.4 (34.5–38.4)	1637	3666	44.7 (42.5–46.9)	0.81 (0.76–0.87)
Syncope	671	4552	14.7 (13.7–15.9)	723	4378	16.5 (15.3–17.8)	0.89 (0.80–0.99)
Confusional state	157	5039	3.1 (2.7–3.6)	265	4710	5.6 (5.0–6.4)	0.55 (0.45–0.67)
Insomnia	387	4838	8.0 (7.2–8.8)	474	4600	10.3 (9.4–11.3)	0.78 (0.68–0.89)
Depression	745	4651	16.0 (14.9–17.2)	909	4306	21.1 (19.8–22.5)	0.76 (0.69–0.84)

Table S83. Incidence Rates of Musculoskeletal Adverse Events in Patients Without Congestive Heart Failure (Stages I, II and III)

	**Dronedarone (N=9,207)**			**Amiodarone (N=9,207)**			**Dronedarone vs Amiodarone**
	**N**	**Patient-time at risk (years)**	**Rate (Per 100 patient-years) (95% CI)**	**N**	**Patient-time at risk (years)**	**Rate (Per 100 patient-years) (95% CI)**	**IRR (95% CI)**
Any musculoskeletal event	345	4261	8.1 (7.3–9.0)	401	3994	10.0 (9.1–11.1)	0.81 (0.70–0.93)
Gait disturbance	229	4600	5.0 (4.4–5.7)	289	4278	6.8 (6.0–7.6)	0.74 (0.62–0.88)
Rhabdomyolysis	18	5089	0.4 (0.2–0.6)	23	4792	0.5 (0.3–0.7)	0.74 (0.40–1.37)
Myalgia	138	4778	2.9 (2.4–3.4)	147	4529	3.3 (2.7–3.8)	0.89 (0.71–1.12)

Table S84. Event Rates of Musculoskeletal Adverse Events in Patients Without Congestive Heart Failure (Stages I, II and III)

	**Dronedarone (N=9,207)**			**Amiodarone (N=9,207)**			**Dronedarone vs Amiodarone**
	**N**	**Patient-time at risk (years)**	**Rate (Per 100 patient-years) (95% CI)**	**N**	**Patient-time at risk (years)**	**Rate (Per 100 patient-years) (95% CI)**	**IRR (95% CI)**
Any musculoskeletal event	2166	4616	46.9 (45.0–48.9)	2284	4341	52.6 (50.5–54.8)	0.89 (0.84–0.94)
Gait disturbance	1524	4842	31.5 (29.9–33.1)	2002	4534	44.2 (42.2–46.1)	0.71 (0.67–0.76)
Rhabdomyolysis	26	5114	0.5 (0.3–0.7)	64	4822	1.3 (1.0–1.7)	0.38 (0.24–0.60)
Myalgia	680	4930	13.8 (12.8–14.9)	358	4662	7.7 (6.9–8.5)	1.79 (1.58–2.04)

Table S85. Incidence Rates of Other Adverse Events in Patients Without Congestive Heart Failure (Stages I, II and III)

	Dronedarone (N=9,207)			Amiodarone (N=9,207)			Dronedarone vs Amiodarone
	**N**	**Patient-time at risk (years)**	**Rate (Per 100 patient-years) (95% CI)**	**N**	**Patient-time at risk (years)**	**Rate (Per 100 patient-years) (95% CI)**	**IRR (95% CI)**
Any Renal event	282	4329	6.5 (5.8–7.3)	342	3993	8.6 (7.7–9.5)	0.76 (0.65–0.89)
Acute kidney	210	4682	4.5 (3.9–5.1)	272	4273	6.4 (5.6–7.2)	0.70 (0.59–0.84)
Renal failure	158	4607	3.4 (2.9–4.0)	188	4354	4.3 (3.7–5.0)	0.79 (0.64–0.98)
Blood Disorders							
Any blood related event	264	4497	5.9 (5.2–6.6)	356	4106	8.7 (7.8–9.6)	0.68 (0.58–0.79)
Anemia	222	4650	4.8 (4.2–5.5)	281	4283	6.6 (5.8–7.4)	0.73 (0.61–0.87)
Thrombocytopenia	82	4935	1.7 (1.3–2.1)	125	4613	2.7 (2.3–3.2)	0.61 (0.46–0.81)
Eye disorders			0.0 (0.0–0.0)				
Any ocular event	167	4726	3.5 (3.0–4.1)	200	4429	4.5 (3.9–5.2)	0.78 (0.64–0.96)
Blindness	24	5082	0.5 (0.3–0.7)	34	4791	0.7 (0.5–1.0)	0.67 (0.39–1.12)
Visual impairment	30	5062	0.6 (0.4–0.9)	54	4706	1.2 (0.9–1.5)	0.52 (0.33–0.81)
Vision blurred	138	4816	2.9 (2.4–3.4)	143	4580	3.1 (2.6–3.7)	0.92 (0.73–1.16)
Skin and subcutaneous tissue disorders<br>Any dermatological event	207	4699	4.4 (3.8–5.1)	178	4483	4.0 (3.4–4.6)	1.11 (0.91–1.36)
Rash	170	4795	3.6 (3.0–4.1)	150	4551	3.3 (2.8–3.9)	1.08 (0.86–1.34)
Pruritus	56	5006	1.1 (0.8–1.5)	41	4753	0.9 (0.6–1.2)	1.30 (0.87–1.94)
Overall							
Any adverse event	197	138	142.3 (123.1–163.6)	222	133	166.44 (145.27–189.84)	0.86 (0.71–1.04)

Table S86. Event Rates of Other Adverse Events in Patients Without Congestive Heart Failure (Stages I, II and III)

	Dronedarone (N=9,207)			Amiodarone (N=9,207)			Dronedarone vs Amiodarone
	N	Patient-time at risk (years)	Rate (Per 100 patient-years) (95% CI)	N	Patient-time at risk (years)	Rate (Per 100 patient-years) (95% CI)	RR (95% CI)
Any Renal event	1461	4633	31.5 (29.9–33.2)	2165	4292	50.44 (48.34–52.62)	0.62 (0.58–0.67)
Acute kidney	994	4863	20.4 (19.2–21.8)	1584	4484	35.33 (33.61–37.11)	0.58 (0.53–0.63)
Renal failure	614	4817	12.8 (11.8–13.8)	870	4561	19.07 (17.83–20.39)	0.67 (0.60–0.74)
Blood Disorders							
Any blood related event	1949	4739	41.1 (39.3–43.0)	1682	4423	38.03 (36.23–39.89)	1.08 (1.01–1.15)
Anemia	1816	4850	37.5 (35.7–39.2)	1555	4535	34.29 (32.61–36.04)	1.09 (1.02–1.17)
Thrombocytopenia	192	5013	3.8 (3.3–4.4)	369	4732	7.80 (7.02–8.64)	0.49 (0.41–0.58)
Eye disorders							
Any ocular event	318	4900	6.5 (5.8–7.2)	445	4610	9.65 (8.78–10.59)	0.67 (0.58–0.78)
Blindness	56	5118	1.1 (0.8–1.4)	86	4834	1.78 (1.42–2.20)	0.61 (0.44–0.86)
Visual impairment	71	5095	1.4 (1.1–1.8)	125	4766	2.62 (2.18–3.12)	0.53 (0.40–0.71)
Vision blurred	217	4969	4.4 (3.8–5.0)	272	4710	5.77 (5.11–6.50)	0.76 (0.63–0.90)
Skin and subcutaneous tissue disorders							
Any dermatological event	324	4899	6.6 (5.9–7.4)	261	4641	5.62 (4.96–6.35)	1.17 (1.00–1.38)
Rash	255	4969	5.1 (4.5–5.8)	225	4690	4.80 (4.19–5.47)	1.07 (0.89–1.28)
Pruritus	78	5064	1.5 (1.2–1.9)	55	4796	1.15 (0.86–1.49)	1.34 (0.95–1.89)
Overall							
Any adverse event	1630	340	479.9 (456.9–503.7)	2492	347	718.09 (690.17–746.85)	0.67 (0.63–0.71)

Table S87. Incidence Rates of Respiratory Related Adverse Events in Patients Who Initiated Amiodarone or Dronedarone Within ≤90 Days Period from Initial AF Diagnosis Date

	**Dronedarone (N=3,632)**			**Amiodarone (N=3,632)**			**Dronedarone vs Amiodarone**
	**N**	**Patient-time at risk (years)**	**Rate (Per 100 patient-years) (95% CI)**	**N**	**Patient-time at risk (years)**	**Rate (Per 100 patient-years) (95% CI)**	**IRR (95% CI)**
Any respiratory event	388	831	46.7 (42.2–51.6)	574	776	74.01 (68.08–80.32)	0.63 (0.56–0.72)
Dyspnea	458	1548	29.6 (26.9–32.4)	671	1666	40.27 (37.28–43.44)	0.74 (0.65–0.83)
Interstitial lung	82	3159	2.6 (2.1–3.2)	105	3718	2.82 (2.31–3.42)	0.92 (0.69–1.23)
Cough	387	2370	16.3 (14.7–18.0)	506	2699	18.75 (17.15–20.46)	0.87 (0.76–0.99)
Pulmonary fibrosis	30	3301	0.9 (0.6–1.3)	36	3829	0.94 (0.66–1.30)	0.97 (0.60–1.57)
Pulmonary toxicity	62	3187	2.0 (1.5–2.5)	82	3762	2.18 (1.73–2.71)	0.89 (0.64–1.24)
Respiratory failure	222	3056	7.3 (6.3–8.3)	304	3280	9.27 (8.25–10.37)	0.78 (0.66–0.93)
Lung disorder	322	1968	16.4 (14.6–18.3)	473	2234	21.18 (19.31–23.17)	0.77 (0.67–0.89)
Wheezing	83	3200	2.6 (2.1–3.2)	99	3736	2.65 (2.15–3.23)	0.98 (0.73–1.31)
Pleural effusion	285	2934	9.7 (8.6–10.9)	404	3016	13.39 (12.12–14.77)	0.73 (0.62–0.84)
Acute respiratory	38	3273	1.2 (0.8–1.6)	86	3759	2.29 (1.83–2.83)	0.51 (0.35–0.74)
Pulmonary edema	151	3160	4.8 (4.1–5.6)	239	3504	6.82 (5.98–7.74)	0.70 (0.57–0.86)
Dyspnea exertional	448	1370	32.7 (29.7–35.9)	680	1394	48.77 (45.17–52.57)	0.67 (0.60–0.76)
Pneumonitis	<5	3335	0.1 (0.0–0.3)	--	--	--	--
Hypoxia	101	3195	3.2 (2.6–3.8)	187	3600	5.19 (4.48–5.99)	0.61 (0.48–0.78)

Table S88. Event Rates of Respiratory Related Adverse Events in Patients Who Initiated Amiodarone or Dronedarone Within ≤90 Days Period from Initial AF Diagnosis Date

	**Dronedarone (N=3,632)**			**Amiodarone (N=3,632)**			**Dronedarone vs Amiodarone**
	**N**	**Patient-time at risk (years)**	**Rate (Per 100 patient-years) (95% CI)**	**N**	**Patient-time at risk (years)**	**Rate (Per 100 patient-years) (95% CI)**	**RR (95% CI)**
Any respiratory event	3041	1189	255.7 (246.7–264.9)	4829	1292	373.89 (363.42–384.59)	0.68 (0.65–0.72)
Dyspnea	1354	1955	69.3 (65.6–73.1)	1917	2216	86.51 (82.68–90.48)	0.80 (0.75–0.86)
Interstitial lung	243	3232	7.5 (6.6–8.5)	374	3796	9.85 (8.88–10.90)	0.76 (0.65–0.90)
Cough	695	2729	25.5 (23.6–27.4)	849	3113	27.27 (25.47–29.17)	0.93 (0.84–1.03)
Pulmonary fibrosis	134	3320	4.0 (3.4–4.8)	158	3864	4.09 (3.48–4.78)	0.99 (0.78–1.24)
Pulmonary toxicity	224	3241	6.9 (6.0–7.9)	256	3825	6.69 (5.90–7.56)	1.03 (0.86–1.23)
Respiratory failure	1502	3187	47.1 (44.8–49.6)	2773	3445	80.50 (77.53–83.55)	0.58 (0.55–0.62)
Lung disorder	1451	2235	64.9 (61.6–68.3)	2155	2656	81.13 (77.74–84.63)	0.80 (0.75–0.86)
Wheezing	106	3273	3.2 (2.7–3.9)	151	3814	3.96 (3.35–4.64)	0.82 (0.64–1.05)
Pleural effusion	1295	3102	41.8 (39.5–44.1)	1792	3301	54.28 (51.79–56.85)	0.77 (0.72–0.83)
Acute respiratory	61	3293	1.9 (1.4–2.4)	286	3816	7.49 (6.65–8.41)	0.25 (0.19–0.33)
Pulmonary edema	298	3252	9.2 (8.2–10.3)	457	3663	12.48 (11.36–13.67)	0.73 (0.63–0.85)
Dyspnea exertional	1427	1787	79.9 (75.8–84.1)	2176	1968	110.59 (105.99–115.33)	0.72 (0.68–0.77)
Pneumonitis	54	3337	1.6 (1.2–2.1)	--	--	--	--
Hypoxia	808	3252	24.8 (23.2–26.6)	1280	3724	34.38 (32.52–36.31)	0.72 (0.66–0.79)

Table S89. Incidence Rates of Cardiac and Vascular Related Adverse Events in Patients Who Initiated Amiodarone or Dronedarone Within ≤90 Days Period from Initial AF Diagnosis Date

	**Dronedarone (N=3,632)**			**Amiodarone (N=3,632)**			**Dronedarone vs Amiodarone**
	**N**	**Patient-time at risk (years)**	**Rate (Per 100 patient-years) (95% CI)**	**N**	**Patient-time at risk (years)**	**Rate (Per 100 patient-years) (95% CI)**	**IRR (95% CI)**
Any cardiac and vascular event	296	293	101.0 (89.9–113.2)	448	364	122.99 (111.86–134.92)	0.82 (0.71–0.95)
Bradycardia	444	2404	18.5 (16.8–20.3)	592	2733	21.66 (19.95–23.48)	0.85 (0.75–0.96)
Dizziness	336	2346	14.3 (12.8–15.9)	432	2859	15.11 (13.72–16.60)	0.95 (0.82–1.09)
Tachycardia	232	2316	10.0 (8.8–11.4)	286	2845	10.05 (8.92–11.29)	1.00 (0.84–1.19)
Hypotension	264	2865	9.2 (8.1–10.4)	375	3139	11.95 (10.77–13.22)	0.77 (0.66–0.90)
Qt prolonged	25	3291	0.8 (0.5–1.1)	43	3831	1.12 (0.81–1.51)	0.68 (0.41–1.11)
Ventricular tachycardia	222	2723	8.2 (7.1–9.3)	207	3238	6.39 (5.55–7.33)	1.28 (1.06–1.54)
Cardiac failure	206	3046	6.8 (5.9–7.8)	354	3404	10.40 (9.35–11.54)	0.65 (0.55–0.77)
Torsade de pointes	--	3336	--	--	--	--	--
Cardiac arrest	27	3318	0.8 (0.5–1.2)	57	3816	1.49 (1.13–1.94)	0.54 (0.34–0.86)
Chest pain	387	1378	28.1 (25.4–31.0)	508	1641	30.96 (28.33–33.77)	0.91 (0.80–1.04)
Ventricular fibrillation	16	3309	0.5 (0.3–0.8)	28	3812	0.73 (0.49–1.06)	0.66 (0.36–1.22)
Peripheral edema	401	2500	16.0 (14.5–17.7)	624	2738	22.79 (21.04–24.65)	0.70 (0.62–0.80)
Congestive heart failure	175	3147	5.6 (4.8–6.5)	276	3590	7.69 (6.81–8.65)	0.72 (0.60–0.87)
Arrhythmia	545	1768	30.8 (28.3–33.5)	621	2064	30.09 (27.77–32.55)	1.02 (0.91–1.15)
Cardiogenic shock	11	3327	0.3 (0.2–0.6)	24	3860	0.62 (0.40–0.93)	0.53 (0.26–1.09)
Cardiorespiratory arrest	31	3312	0.9 (0.6–1.3)	63	3785	1.66 (1.28–2.13)	0.56 (0.37–0.86)
Gastrointestinal hemorrhage	187	3007	6.2 (5.4–7.2)	210	3517	5.97 (5.19–6.84)	1.04 (0.86–1.27)

Table S90. Event Rates of Cardiac and Vascular Related Adverse Events in Patients Who Initiated Amiodarone or Dronedarone Within ≤90 Days Period from Initial AF Diagnosis Date

	**Dronedarone (N=3,632)**			**Amiodarone (N=3,632)**			**Dronedarone vs Amiodarone**
	**N**	**Patient-time at risk (years)**	**Rate (Per 100 patient-years) (95% CI)**	**N**	**Patient-time at risk (years)**	**Rate (Per 100 patient-years) (95% CI)**	**RR (95% CI)**
Any cardiac and vascular event	2364	594	398.0 (382.1–414.4)	4105	781	525.46 (509.50–541.78)	0.76 (0.72–0.80)
Bradycardia	1175	2788	42.2 (39.8–44.6)	1531	3256	47.03 (44.70–49.44)	0.90 (0.83–0.97)
Dizziness	838	2665	31.4 (29.4–33.7)	1013	3200	31.66 (29.74–33.67)	0.99 (0.91–1.09)
Tachycardia	438	2505	17.5 (15.9–19.2)	519	3054	16.99 (15.56–18.52)	1.03 (0.91–1.17)
Hypotension	827	3064	27.0 (25.2–28.9)	1683	3427	49.12 (46.80–51.52)	0.55 (0.51–0.60)
Qt prolonged	46	3316	1.4 (1.0–1.9)	75	3874	1.94 (1.52–2.43)	0.72 (0.50–1.03)
Ventricular tachycardia	577	2893	19.9 (18.4–21.6)	527	3424	15.39 (14.10–16.76)	1.30 (1.15–1.46)
Cardiac failure	1519	3207	47.4 (45.0–49.8)	3660	3691	99.17 (95.98–102.44)	0.48 (0.45–0.51)
Torsade de pointes	--	--	--	--	--	--	--
Cardiac arrest	46	3325	1.4 (1.0–1.9)	235	3841	6.12 (5.36–6.95)	0.23 (0.16–0.31)
Chest pain	757	1734	43.7 (40.6–46.9)	1290	2082	61.95 (58.62–65.43)	0.70 (0.64–0.77)
Ventricular fibrillation	33	3320	1.0 (0.7–1.4)	61	3848	1.59 (1.21–2.04)	0.63 (0.41–0.96)
Peripheral edema	1153	2853	40.4 (38.1–42.8)	1711	3247	52.69 (50.22–55.25)	0.77 (0.71–0.83)
Congestive heart failure	1637	3282	49.9 (47.5–52.4)	3181	3808	83.53 (80.65–86.48)	0.60 (0.56–0.63)
Arrhythmia	1296	2273	57.0 (53.9–60.2)	1508	2574	58.58 (55.66–61.62)	0.97 (0.90–1.05)
Cardiogenic shock	29	3334	0.9 (0.6–1.3)	92	3876	2.37 (1.91–2.91)	0.37 (0.24–0.56)
Cardiorespiratory arrest	50	3322	1.5 (1.1–2.0)	245	3820	6.41 (5.64–7.27)	0.23 (0.17–0.32)
Gastrointestinal hemorrhage	1167	3171	36.8 (34.7–39.0)	895	3665	24.42 (22.85–26.08)	1.51 (1.38–1.64)

Table S91. Incident Rates of General Adverse Events in Patients Who Initiated Amiodarone or Dronedarone Within ≤90 Days Period from Initial AF Diagnosis Date

	**Dronedarone (N=3,632)**			**Amiodarone (N=3,632)**			**Dronedarone vs Amiodarone**
	**N**	**Patient-time at risk (years)**	**Rate (Per 100 patient-years) (95% CI)**	**N**	**Patient-time at risk (years)**	**Rate (Per 100 patient-years) (95% CI)**	**IRR (95% CI)**
Any general event	616	1401	44.0 (40.6–47.6)	834	1467	56.87 (53.07–60.86)	0.77 (0.70–0.86)
Asthenia	494	2043	24.2 (22.1–26.4)	675	2311	29.20 (27.04–31.49)	0.83 (0.74–0.93)
Fatigue	524	1986	26.4 (24.2–28.8)	675	2289	29.49 (27.31–31.80)	0.90 (0.80–1.00)
Weight decreased	127	3115	4.1 (3.4–4.9)	176	3636	4.84 (4.15–5.61)	0.84 (0.67–1.06)
Weight increased	30	3248	0.9 (0.6–1.3)	52	3807	1.37 (1.02–1.79)	0.68 (0.43–1.06)
Malaise	445	2158	20.6 (18.8–22.6)	614	2410	25.48 (23.50–27.57)	0.81 (0.72–0.91)
Edema	403	2497	16.1 (14.6–17.8)	620	2726	22.74 (20.99–24.60)	0.71 (0.63–0.80)

Table S92. Event Rates of General Adverse Events in Patients Who Initiated Amiodarone or Dronedarone Within ≤90 Days Period from Initial AF Diagnosis Date

	**Dronedarone (N=12,210)**			**Amiodarone (N=12,210)**			**Dronedarone vs Amiodarone**
	**N**	**Patient-time at risk (years)**	**Rate (Per 100 patient-years) (95% CI)**	**N**	**Patient-time at risk (years)**	**Rate (Per 100 patient-years) (95% CI)**	**RR (95% CI)**
Any general event	3728	1952	191.0 (184.9–197.3)	5335	2163	246.63 (240.06–253.34)	0.77 (0.74–0.81)
Asthenia	1272	2436	52.2 (49.4–55.2)	2306	2832	81.43 (78.14–84.82)	0.64 (0.60–0.69)
Fatigue	1302	2432	53.5 (50.7–56.5)	1725	2825	61.06 (58.21–64.01)	0.88 (0.82–0.94)
Weight decreased	414	3228	12.8 (11.6–14.1)	462	3775	12.24 (11.15–13.41)	1.05 (0.92–1.20)
Weight increased	39	3272	1.2 (0.9–1.6)	74	3844	1.93 (1.51–2.42)	0.62 (0.42–0.91)
Malaise	1388	2524	55.0 (52.2–58.0)	2140	2901	73.77 (70.68–76.97)	0.75 (0.70–0.80)
Edema	1175	2852	41.2 (38.9–43.6)	1683	3228	52.14 (49.67–54.69)	0.79 (0.73–0.85)

Table S93. Incidence Rates of Endocrine Adverse Events in Patients Who Initiated Amiodarone or Dronedarone Within ≤90 Days Period from Initial AF Diagnosis Date

	**Dronedarone (N=3,632)**			**Amiodarone (N=3,632)**			**Dronedarone vs Amiodarone**
	**N**	**Patient-time at risk (years)**	**Rate (Per 100 patient-years) (95% CI)**	**N**	**Patient-time at risk (years)**	**Rate (Per 100 patient-years) (95% CI)**	**IRR (95% CI)**
Any endocrine event	232	2284	10.2 (8.9–11.6)	506	2573	19.66 (17.99–21.45)	0.52 (0.44–0.60)
Hyperthyroidism	38	3220	1.2 (0.8–1.6)	79	3772	2.09 (1.66–2.61)	0.56 (0.38–0.83)
Hypothyroidism	167	2470	6.8 (5.8–7.9)	422	2763	15.27 (13.85–16.80)	0.44 (0.37–0.53)
Thyroid disorder	95	3073	3.1 (2.5–3.8)	136	3671	3.70 (3.11–4.38)	0.83 (0.64–1.08)
Decreased appetite	60	3269	1.8 (1.4–2.4)	72	3793	1.90 (1.49–2.39)	0.97 (0.69–1.36)

Table S94. Event Rates of Endocrine Adverse Events in Patients Who Initiated Amiodarone or Dronedarone Within ≤90 Days Period from Initial AF Diagnosis Date

	**Dronedarone (N=12,210)**			**Amiodarone (N=12,210)**			**Dronedarone vs Amiodarone**
	**N**	**Patient-time at risk (years)**	**Rate (Per 100 patient-years) (95% CI)**	**N**	**Patient-time at risk (years)**	**Rate (Per 100 patient-years) (95% CI)**	**RR (95% CI)**
Any endocrine event	880	2494	35.3 (33.0–37.7)	2302	2983	77.18 (74.06–80.40)	0.46 (0.42–0.49)
Hyperthyroidism	89	3257	2.7 (2.2–3.4)	212	3834	5.53 (4.81–6.33)	0.49 (0.39–0.63)
Hypothyroidism	525	2626	20.0 (18.3–21.8)	1925	3119	61.72 (59.00–64.54)	0.32 (0.29–0.36)
Thyroid disorder	234	3166	7.4 (6.5–8.4)	390	3761	10.37 (9.37–11.45)	0.71 (0.61–0.84)
Decreased appetite	252	3307	7.6 (6.7–8.6)	147	3837	3.83 (3.24–4.50)	1.99 (1.62–2.44)

Table S95. Incidence Rates of Gastrointestinal and Hepatobiliary Adverse Events in Patients Who Initiated Amiodarone or Dronedarone Within ≤90 Days Period from Initial AF Diagnosis Date

	**Dronedarone (N=3,632)**			**Amiodarone (N=3,632)**			**Dronedarone vs Amiodarone**
	**N**	**Patient-time at risk (years)**	**Rate (Per 100 patient-years) (95% CI)**	**N**	**Patient-time at risk (years)**	**Rate (Per 100 patient-years) (95% CI)**	**IRR (95% CI)**
Any gastrointestinal and hepatobiliary event	506	2046	24.7 (22.6–27.0)	582	2207	26.37 (24.27–28.60)	0.94 (0.83–1.06)
Nausea	239	2794	8.6 (7.5–9.7)	414	3115	13.29 (12.04–14.63)	0.64 (0.55–0.75)
Vomiting	151	3011	5.0 (4.3–5.9)	327	3321	9.85 (8.81–10.97)	0.51 (0.42–0.62)
Diarrhea	227	2934	7.7 (6.8–8.8)	220	3403	6.46 (5.64–7.38)	1.20 (0.99–1.44)
Abdominal pain	405	2413	16.8 (15.2–18.5)	467	2633	17.74 (16.16–19.42)	0.95 (0.83–1.08)
Liver injury	<5	3335	--	<5	3890	--	--
Hepatic failure	15	3308	0.5 (0.3–0.8)	35	3856	0.91 (0.63–1.26)	0.50 (0.27–0.91)

Table S96. Event Rates of Gastrointestinal and Hepatobiliary Adverse Events in Patients Who Initiated Amiodarone or Dronedarone Within ≤90 Days Period from Initial AF Diagnosis Date

	**Dronedarone (N=3,632)**			**Amiodarone (N=3,632)**			**Dronedarone vs Amiodarone**
	**N**	**Patient-time at risk (years)**	**Rate (Per 100 patient-years) (95% CI)**	**N**	**Patient-time at risk (years)**	**Rate (Per 100 patient-years) (95% CI)**	**RR (95% CI)**
Any gastrointestinal and hepatobiliary event	2241	2458	91.2 (87.4–95.0)	2901	2660	109.05 (105.12–113.09)	0.84 (0.79–0.88)
Nausea	795	3016	26.4 (24.6–28.3)	1279	3418	37.42 (35.39–39.52)	0.70 (0.64–0.77)
Vomiting	505	3126	16.2 (14.8–17.6)	928	3542	26.20 (24.54–27.94)	0.62 (0.55–0.69)
Diarrhea	594	3139	18.9 (17.4–20.5)	741	3549	20.88 (19.40–22.44)	0.91 (0.81–1.01)
Abdominal pain	911	2717	33.5 (31.4–35.8)	1130	2999	37.68 (35.52–39.94)	0.89 (0.81–0.97)
Liver injury	<5	3337	--	<5	--	--	--
Hepatic failure	101	3317	3.0 (2.5–3.7)	104	3877	2.68 (2.19–3.25)	1.13 (0.86–1.49)

Table S97. Incidence Rates of Neurological Adverse Events in Patients Who Initiated Amiodarone or Dronedarone Within ≤90 Days Period from Initial AF Diagnosis Date

	**Dronedarone (N=3,632)**			**Amiodarone (N=3,632)**			**Dronedarone vs Amiodarone**
	**N**	**Patient-time at risk (years)**	**Rate (Per 100 patient-years) (95% CI)**	**N**	**Patient-time at risk (years)**	**Rate (Per 100 patient-years) (95% CI)**	**IRR (95% CI)**
Any neurological event	406	2106	19.3 (17.5–21.3)	562	2361	23.80 (21.87–25.85)	0.81 (0.71–0.92)
Syncope	222	2733	8.1 (7.1–9.3)	285	3185	8.95 (7.94–10.05)	0.91 (0.76–1.08)
Confusional state	126	3139	4.0 (3.3–4.8)	192	3550	5.41 (4.67–6.23)	0.74 (0.59–0.93)
Insomnia	164	3027	5.4 (4.6–6.3)	226	3508	6.44 (5.63–7.34)	0.84 (0.69–1.03)
Depression	188	2852	6.6 (5.7–7.6)	256	3226	7.94 (6.99–8.97)	0.83 (0.69–1.00)

Table S98. Event Rates of Neurological Adverse Events in Patients Who Initiated Amiodarone or Dronedarone Within ≤90 Days Period from Initial AF Diagnosis Date

	**Dronedarone (N=3,632)**			**Amiodarone (N=3,632)**			**Dronedarone vs Amiodarone**
	**N**	**Patient-time at risk (years)**	**Rate (Per 100 patient-years) (95% CI)**	**N**	**Patient-time at risk (years)**	**Rate (Per 100 patient-years) (95% CI)**	**RR (95% CI)**
Any neurological event	1506	2443	61.6 (58.6–64.8)	2614	2781	93.99 (90.42–97.67)	0.66 (0.62–0.70)
Syncope	690	2904	23.8 (22.0–25.6)	1230	3407	36.10 (34.11–38.18)	0.66 (0.60–0.72)
Confusional state	475	3226	14.7 (13.4–16.1)	752	3680	20.43 (19.00–21.95)	0.72 (0.64–0.81)
Insomnia	280	3178	8.8 (7.8–9.9)	554	3690	15.01 (13.79–16.32)	0.59 (0.51–0.68)
Depression	541	2995	18.1 (16.6–19.7)	1069	3426	31.20 (29.36–33.13)	0.58 (0.52–0.64)

Table S99. Incidence Rates of Musculoskeletal Adverse Events in Patients Who Initiated Amiodarone or Dronedarone Within ≤90 Days Period from Initial AF Diagnosis Date

	**Dronedarone (N=3,632)**			**Amiodarone (N=3,632)**			**Dronedarone vs Amiodarone**
	**N**	**Patient-time at risk (years)**	**Rate (Per 100 patient-years) (95% CI)**	**N**	**Patient-time at risk (years)**	**Rate (Per 100 patient-years) (95% CI)**	**IRR (95% CI)**
Any musculoskeletal event	314	2644	11.9 (10.6–13.3)	489	2808	17.41 (15.90–19.03)	0.68 (0.59–0.79)
Gait disturbance	239	2854	8.4 (7.4–9.5)	422	3035	13.91 (12.61–15.30)	0.60 (0.51–0.71)
Rhabdomyolysis	28	3289	0.9 (0.6–1.2)	36	3845	0.94 (0.66–1.30)	0.91 (0.55–1.49)
Myalgia	87	3113	2.8 (2.2–3.5)	124	3614	3.43 (2.85–4.09)	0.81 (0.62–1.07)

Table S100. Event Rates of Musculoskeletal Adverse Events in Patients Who Initiated Amiodarone or Dronedarone Within ≤90 Days Period from Initial AF Diagnosis Date

	**Dronedarone (N=3,632)**			**Amiodarone (N=3,632)**			**Dronedarone vs Amiodarone**
	**N**	**Patient-time at risk (years)**	**Rate (Per 100 patient-years) (95% CI)**	**N**	**Patient-time at risk (years)**	**Rate (Per 100 patient-years) (95% CI)**	**RR (95% CI)**
Any musculoskeletal event	2316	2926	79.2 (76.0–82.5)	4625	3224	143.46 (139.36–147.66)	0.55 (0.52–0.58)
Gait disturbance	1886	3082	61.2 (58.5–64.0)	4380	3388	129.30 (125.50–133.18)	0.47 (0.45–0.50)
Rhabdomyolysis	250	3308	7.6 (6.7–8.6)	98	3872	2.53 (2.05–3.08)	2.98 (2.36–3.77)
Myalgia	280	3190	8.8 (7.8–9.9)	295	3721	7.93 (7.05–8.89)	1.11 (0.94–1.30)

Table S101. Incidence Rates of Other Adverse Events in Patients Who Initiated Amiodarone or Dronedarone Within ≤90 Days Period from Initial AF Diagnosis Date

	**Dronedarone (N=3,632)**			**Amiodarone (N=3,632)**			**Dronedarone vs Amiodarone**
	**N**	**Patient-time at risk (years)**	**Rate (Per 100 patient-years) (95% CI)**	**N**	**Patient-time at risk (years)**	**Rate (Per 100 patient-years) (95% CI)**	**IRR (95% CI)**
Renal and Urinary disorders							
Any renal event	303	2533	12.0 (10.7–13.4)	473	2687	17.60 (16.05–19.26)	0.68 (0.59–0.79)
Acute kidney	238	2894	8.2 (7.2–9.3)	365	3087	11.82 (10.64–13.10)	0.70 (0.59–0.82)
Renal failure	196	2749	7.1 (6.2–8.2)	327	3077	10.63 (9.51–11.84)	0.67 (0.56–0.80)
Blood Disorders							
Any blood related event	269	2708	9.9 (8.8–11.2)	423	3029	13.96 (12.66–15.36)	0.71 (0.61–0.83)
Anemia	226	2822	8.0 (7.0–9.1)	379	3182	11.91 (10.74–13.17)	0.67 (0.57–0.79)
Thrombocytopenia	82	3165	2.6 (2.1–3.2)	117	3653	3.20 (2.65–3.84)	0.81 (0.61–1.07)
Eye disorders							
Any ocular event	159	2987	5.3 (4.5–6.2)	213	3453	6.17 (5.37–7.06)	0.86 (0.70–1.06)
Blindness	15	3297	0.5 (0.3–0.8)	37	3842	0.96 (0.68–1.33)	0.47 (0.26–0.86)
Visual impairment	47	3225	1.5 (1.1–1.9)	82	3754	2.18 (1.74–2.71)	0.67 (0.47–0.96)
Vision blurred	113	3108	3.6 (3.0–4.4)	126	3612	3.49 (2.91–4.15)	1.04 (0.81–1.34)
Skin and subcutaneous tissue disorders							
Any dermatological event	158	3057	5.2 (4.4–6.0)	174	3563	4.88 (4.18–5.67)	1.06 (0.85–1.31)
Rash	126	3103	4.1 (3.4–4.8)	147	3623	4.06 (3.43–4.77)	1.00 (0.79–1.27)
Pruritus	45	3266	1.4 (1.0–1.8)	45	3809	1.18 (0.86–1.58)	1.17 (0.77–1.76)
Overall							
Any adverse event	75	63	119.3 (93.8–149.5)	118	62	189.24 (156.64–226.63)	0.63 (0.47–0.84)

Table S102. Event Rates of Other Adverse Events in Patients Who Initiated Amiodarone or Dronedarone Within ≤90 Days Period from Initial AF Diagnosis Date

	**Dronedarone (N=3,632)**			**Amiodarone (N=3,632)**			**Dronedarone vs Amiodarone**
	**N**	**Patient-time at risk (years)**	**Rate (Per 100 patient-years) (95% CI)**	**N**	**Patient-time at risk (years)**	**Rate (Per 100 patient-years) (95% CI)**	**RR (95% CI)**
Renal and Urinary disorders			0.0 (0.0–0.0)				
Any renal event	1805	2794	64.6 (61.7–67.7)	4421	3055	144.73 (140.49–149.06)	0.45 (0.42–0.47)
Acute kidney	1177	3062	38.4 (36.3–40.7)	2534	3342	75.82 (72.89–78.83)	0.51 (0.47–0.54)
Renal failure	1019	2949	34.6 (32.5–36.7)	2670	3371	79.20 (76.22–82.26)	0.44 (0.41–0.47)
Blood Disorders							
Any blood related event	2296	2964	77.5 (74.3–80.7)	2492	3361	74.14 (71.26–77.11)	1.04 (0.99–1.11)
Anemia	2163	3040	71.2 (68.2–74.2)	2225	3497	63.62 (61.00–66.32)	1.12 (1.05–1.19)
Thrombocytopenia	177	3224	5.5 (4.7–6.4)	522	3737	13.97 (12.79–15.22)	0.39 (0.33–0.47)
Eye disorders							
Any ocular event	381	3141	12.1 (10.9–13.4)	956	3622	26.39 (24.75–28.12)	0.46 (0.41–0.52)
Blindness	27	3315	0.8 (0.5–1.2)	177	3870	4.57 (3.92–5.30)	0.18 (0.12–0.27)
Visual impairment	150	3260	4.6 (3.9–5.4)	522	3816	13.68 (12.53–14.90)	0.34 (0.28–0.40)
Vision blurred	214	3223	6.6 (5.8–7.6)	298	3721	8.01 (7.12–8.97)	0.83 (0.70–0.99)
Skin and subcutaneous tissue disorders							
Any dermatological event	253	3196	7.9 (7.0–9.0)	348	3700	9.41 (8.44–10.45)	0.84 (0.72–0.99)
Rash	190	3219	5.9 (5.1–6.8)	241	3739	6.45 (5.66–7.31)	0.91 (0.76–1.11)
Pruritus	66	3307	2.0 (1.5–2.5)	115	3845	2.99 (2.47–3.59)	0.67 (0.49–0.90)
Overall							
Any adverse event	1294	145	891.0 (843.1–940.9)	2227	172	1,292.40 (1,239.28–1,347.22)	0.69 (0.64–0.74)
